# The Genboree Microbiome Toolset and the analysis of 16S rRNA microbial sequences

**DOI:** 10.1186/1471-2105-13-S13-S11

**Published:** 2012-08-24

**Authors:** Kevin Riehle, Cristian Coarfa, Andrew Jackson, Jun Ma, Arpit Tandon, Sameer Paithankar, Sriram Raghuraman, Toni-Ann Mistretta, Delphine Saulnier, Sabeen Raza, Maria Alejandra Diaz, Robert Shulman, Kjersti Aagaard, James Versalovic, Aleksandar Milosavljevic

**Affiliations:** 1 Molecular & Human Genetics, Baylor College of Medicine, Houston, TX 77030, USA; 2 Obstetrics and Gynecology, Baylor College of Medicine, Houston, TX 77030, USA; 3 Pathology & Immunology, Baylor College of Medicine, Houston, TX 77030, USA; 4 Nizo Food Research, Ede, 6710 BA, The Netherlands; 5 Department of Pediatrics, Baylor College of Medicine, Houston, TX 77030, USA

## Abstract

**Background:**

Microbial metagenomic analyses rely on an increasing number of publicly available tools. Installation, integration, and maintenance of the tools poses significant burden on many researchers and creates a barrier to adoption of microbiome analysis, particularly in translational settings.

**Methods:**

To address this need we have integrated a rich collection of microbiome analysis tools into the Genboree Microbiome Toolset and exposed them to the scientific community using the Software-as-a-Service model via the Genboree Workbench. The Genboree Microbiome Toolset provides an interactive environment for users at all bioinformatic experience levels in which to conduct microbiome analysis. The Toolset drives hypothesis generation by providing a wide range of analyses including alpha diversity and beta diversity, phylogenetic profiling, supervised machine learning, and feature selection.

**Results:**

We validate the Toolset in two studies of the gut microbiota, one involving obese and lean twins, and the other involving children suffering from the irritable bowel syndrome.

**Conclusions:**

By lowering the barrier to performing a comprehensive set of microbiome analyses, the Toolset empowers investigators to translate high-volume sequencing data into valuable biomedical discoveries.

## Background

The Human Microbiome Project (HMP) aims to improve the understanding of the microbiome and the factors that influence the distribution and evolution of constituent microorganisms in a healthy human population cohort. A number of focused sub-projects within HMP aim to detect and interpret perturbations of microbiomes associated with human diseases [1]. These efforts are being been aided by accelerating technical and methodological advancements in sequencing and computational technologies. The 16S rRNA gene has proven to be a useful initial genomic target to identify and differentiate distinct microbial profiles, such as those in human fecal samples [2]. Determining the abundance (and inferred function) of each type of microbe (community profiling) is less expensive using 16S rRNA than bacterial genomic DNA because only one representative gene from each genome is examined [3]. As the focus widens from 16S rRNA to genomic sequencing, as the costs of sequencing decrease, and the amounts of publically available data increase, the technological and methodological bottleneck on the road to discoveries will shift from sequencing to bioinformatic analysis.

The new bioinformatic bottleneck will need to be addressed in an innovative way, particularly with regard to translational research. In the field of metagenomics, the productivity of translational research is increasingly determined by the amount of effort required to integrate large volumes of “omics” data with clinical metadata and analyze the integrated data sets using latest tools to generate biomedically relevant testable hypotheses. There are multiple mature, open source tools for 16S rRNA gene analyses, that are well maintained and widely used within the scientific community, such as QIIME (Quantitative Insights Into Microbial Ecology) [4] and mothur [5]. We have integrated those tools within the Genboree Microbiome Toolset and deployed them through the Genboree Workbench [6] using the Software-as-a-Service model. To enable researchers to gain insight into clinically relevant phenotypes, while accounting for the most significant confounding factors, we have designed the Genboree Microbiome Toolset to be “sample centric”. The Toolset enables users to associate metadata with samples for both supervised sample classification and unsupervised analyses. The toolset also enables analyses of alpha diversity and beta diversity, phylogenetic analysis, and feature selection. The Toolset assures reproducibility of results reported in journal publications and provides default settings at each step that can be customized by the user to reflect their preference or to follow a standard protocol.

The Toolset is deployed using the easy-to-use web-based GUI environment of the Genboree Workbench. The Genboree environment enables web-based collaboration while allowing access control to sensitive data. By virtue of integration through the Genboree Workbench, all the functionality that is accessible interactively through the Toolset is also accessible programmatically via a custom REST Application Programming Interface [7,8], the Genboree REST API , thus allowing programmatic extension, customization, and web-based integration.

## Methods

**Figure 1:**
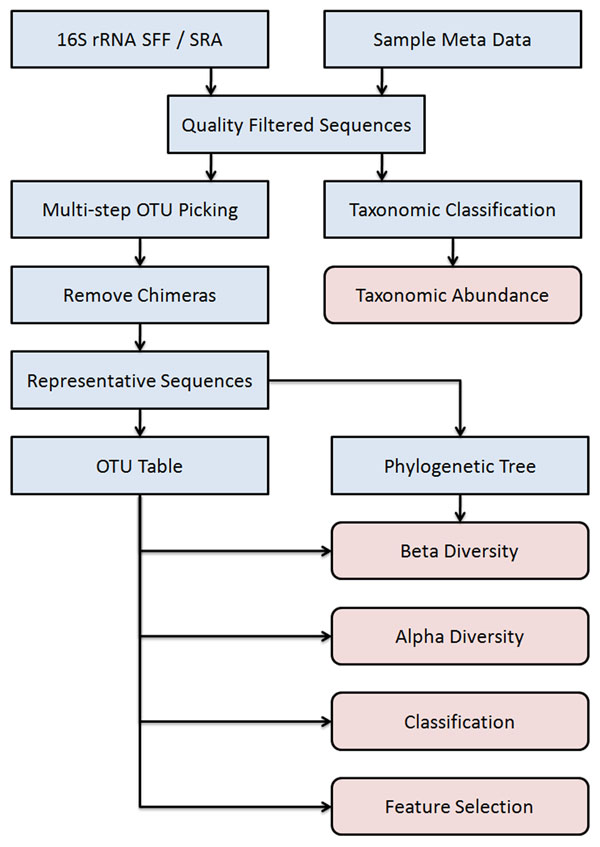
**Genboree Microbiome Toolset Dataflow**. The flow begins by producing quality filtered sequences from the 16S rRNA sequences and the sample metadata. These can be passed to the taxonomic classification pipeline for taxonomic abundance reports or to the multi-step OTU picking pipeline for alpha diversity, beta diversity, classification using supervised machine learning, and feature selection.

The initial step in the Toolset flow is the extraction of sequences for each sample from the input sequence files followed by a set of quality filters, as shown in Fig. 1. Operational Taxonomic Unit (OTU) generation is accomplished by a multi-step OTU picking algorithm that generates representative sequences from the complete sequence data set and produces an OTU table as a result. The OTU table provides users with a matrix of data necessary for downstream analyses, such as alpha diversity, beta diversity [9], classification by supervised machine learning, and feature selection.

### Linking quality filtered sequences to sample metadata

Massively parallel sequencing platforms such as 454 typically produce multiplexed sequence files that contain sequences from more than one sample. Samples and their associated metadata are linked to corresponding sequences using the MID (multiplex identifier), proximal primer, and distal primer. For the purpose of downstream analyses, samples may be analyzed individually or as sample sets.

### Taxonomic classification via the Ribosomal Database Project (RDP) Classifier

The Microbiome Toolset integrates the Ribosomal Database Project (RDP) Classifier [10], which performs taxonomic classification of individual 16S rRNA sequences based on a naive Bayesian classification. The output of RDP Classifier 2.1 (and newer) assigns each sequence to the most specific taxon level (from the Domain to the Genus levels). Sequence counts are then calculated for distinct taxa at each of the levels and combined to produce absolute and relative abundance profiles at each level.

### Creating the Operational Taxonomic Unit (OTU) table and representative sequences for phylogenetic tree reconstruction

The Microbiome Toolset integrates a range of analyses based on OTUs, groups of sequences distinguished by their mutual similarity. The QIIME package [4] performs multi-step chained OTU picking using multiple third party tools, including cd-hit [11], mothur [5], and uclust [12]. High speed is achieved by using a rough, fast method to collapse sequence groups that have a high level of similarity, followed by a more computationally demanding and rigorous OTU picking step. Chimeric sequences, which can be falsely detected as novel organisms, resulting in the artificial inflation of diversity are detected and removed using Chimera Slayer [13]. A set of sequences representing each OTU are used for phylogenetic tree reconstruction. Sequence counts per OTU and per sample are summarized in an output OTU table, which is a key input for downstream analyses.

### Phylogenetic analysis

The Toolset enables comparison and visualization of representative sequences in the context of a phylogenetic tree. For comparison, we use UniFrac to examine differences between microbiome communities by measuring the distance between sample-specific sets of taxa in a phylogenetic tree. The phylogenetic distances estimate the degree of evolutionary divergence between different representative sequences [14], not just the degree of their sequence-level differences.

Phylogenetic differences may be visualized using tools such as the interactive Tree Of Life (iTOL), which supports upload, display, and manipulation of phylogenetic trees [15]. The Microbiome Toolset automatically generates a multi-level circular phylogenetic tree based on metadata and taxonomic information via iTOL’s API. Sample-associated metadata can be used to visually detect phylogenetic distribution biases in specific samples or sample sets.

### Beta diversity analysis

Beta diversity analysis considers biodiversity between groups of samples, focusing on the elements that are either unique to or shared among groups of samples. The Toolset includes the QIIME pipeline, which currently includes 14 non-phylogenetic, 9 binary non-phylogenetic, and 6 phylogenetic metrics. Beta diversity plots are generated for all the metrics so that the user can explore the differences between various microbiomes. This approach was adopted to increase the likelihood of detecting biologically significant patterns visible only when using specific metrics. A case in point is the Canberra metric, an equal-weight metric that standardizes the input such that each OTU affects the distance value equally [16]. This equal-weight, non-phylogenetic method was successfully used to distinguish between two tundra communities which could not be distinguished using chord distance, an alternative non-phylogenetic method biased towards largely abundant taxa [17].

The phylogenetic-based UniFrac [14] algorithm enables the analysis of different microbiomes by providing both a quantitative measurement, using weighted UniFrac, and a qualitative measurement, using unweighted UniFrac. Quantitative measures of a phylogenetic method shows changes in OTU abundance such as those caused by nutrient shifts whereas qualitative measures detect differences in microbiomes based on presence or absence of species in specific environments such as high- or low-temperature [18].

For each beta diversity distance metric that is utilized, the results are displayed for the top three principal coordinates using Principal Coordinates Analysis (PCoA) for both normalized and non-normalized OTU tables. Normalizing the OTU tables on a sample-by-sample basis allows the researcher to account for potential variability in sequencing depth. PCoA plots in both 2D and 3D formats are provided in embedded HTML for further analysis. Beta diversity clustering has been utilized to show that three different individuals can be discriminated based on their distinct skin (fingertip) microbiomes obtained from their keyboards [19].

### Classification and selection of discriminating features

It is frequently of interest to identify a small and assayable set of OTUs that can distinguish between sets of samples with different phenotypes. To meet this goal, a supervised machine learning pipeline was developed and exposed within the Toolset. The pipeline determines the success rate of classifying groups of samples and selects the features that best discriminate groups of samples. The pipeline utilizes the R package randomForest [20] for supervised learning and Boruta [21] for feature selection. The input to the pipeline consists of the OTU table from QIIME pipeline and the sample metadata collected using the Sample Importer.

The algorithm randomForest employs an ensemble approach based on the Classification and Regression Trees (CART) method. It generates and evaluates many classification trees for discrete data or regression trees for continuous data. The classification error rate is measured by the out-of-bag (OOB) error estimation for each metadata category. Because randomForest does not inherently provide for feature selection [22], we employed the R package Boruta, a feature selection algorithm built around the randomForest algorithm. The Z score, computed by dividing the average loss of accuracy by its standard deviation, associates an importance measure with the randomForest results. In addition to the output files generated by the randomForest and Boruta packages, the Toolset provides a summary file that combines the OTU number, taxonomic labels generated by RDP, metrics of OTU distribution for each metadata group (min, max, and quartiles), Mann-Whitney [23] U and Z scores, box plot coordinates, and directional change (calculated by comparing Mann-Whitney U scores for each sample group).

The Toolset also includes an R script to visualize the top performing features in a box plot format that mimics the plot from Qin and Li’s study of the human gut microbial gene catalogue [24]. Such plots provide concise and informative visual summaries of directional change and relative abundance for the most discriminating features.

### Integration of the Microbiome Toolset within the Genboree Workbench

**Figure 2:**
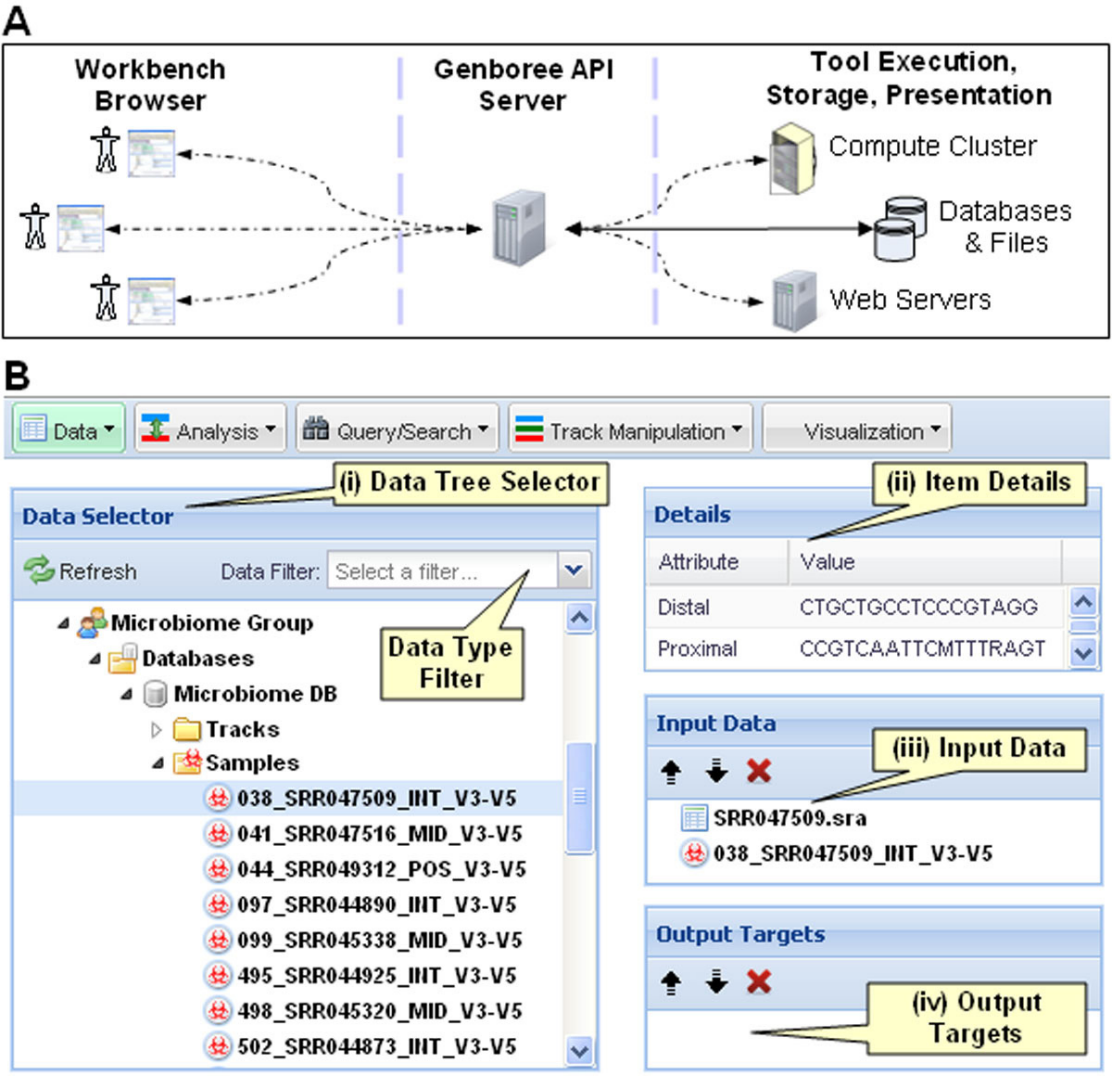
**Genboree Workbench interface.** (A) A summary of interactions between the researchers using the web-based Workbench UI, which both exposes accessible data and permits configuration of Toolset analyses to be run on a compute cluster. The dashed lines indicate communication via the Genboree API. (B) Within the Workbench UI, a folder system in the left pane (i) lists sequencing results, tool results, and all other data types organized by Groups at the top level, and by Databases and Project pages at the second level. The three right panes indicate: attributes of selected data objects (ii), tool input data (iii), and target destinations for tool outputs (iv).

The Microbiome Toolset is integrated within the Genboree Workbench, whose user interface (UI) is a JavaScript-driven web page displayed in the user’s web browser. The Toolset and data are hosted on a remote server which has access to scalable computing resources, removing any hardware or software maintenance burden for the user. The Workbench user interface, illustrated in Fig. 2B, allows users to perform analysis steps summarized in Fig. 1 using the tools from the Microbiome Toolset. The Workbench exclusively uses Genboree REST Application Programming Interfaces (REST APIs) to communicate with the Genboree server (Fig. 2A). Hence, all the functionality accessible via the Workbench, including the Microbiome Toolset, is also accessible programmatically. This design makes it possible to integrate Microbiome Toolset functionality into local pipelines, or to extend its functionality using custom analysis pipelines that run locally on the users’ computers or elsewhere on the Web.

The Genboree Workbench allows users to navigate through a plethora of data sources and match the data to available tools. The interface exposes various data sources to a user via a folder in the left pane (Fig. 2B-i). The first level is that of user Groups corresponding to a specific collaboration or more permanent groupings such as a specific research laboratory. The next level in the folder system contains Projects and Databases, which further encompass Annotation Objects, Annotation Tracks, Samples, Queries, and unstructured Files. Additional information about a selected object appears in the details panel (Fig. 2B-ii). A user can also use that area to download data items onto a local computer.

The tool interface is drag-and-drop: tool inputs are dragged from the folder system into the Input Data Panel (Fig. 2B-iii) and the target databases are dragged from the folder system into the Output Targets panel (Fig. 2B-iv). Each tool has specific input and output requirements. If these requirements are met, the tool is highlighted in green in the menu and can be invoked by selecting the tool from the menu. If the tool is not highlighted in green, a click on the tool in the menu displays a help dialog which will list the input and output requirements and other useful infomation.

The Microbiome Toolset is founded on a sample-centric data and analysis model. Prior to performing analysis steps using the Toolset, as illustrated in Fig. 1., the users must establish links between input sequences and the samples from which the sequences were derived. The Microbiome Toolset and all other Genboree Workbench tools are invoked with customizable default settings. The analysis steps illustrated in Fig. 1 are seamlessly integrated in the Microbiome Toolset pipeline and require no file format conversions.

The inputs and outputs of specific analysis steps are stored in Genboree Databases and Project pages. Genboree Project pages are automatically generated by the tools but may also be manually edited. Serving a role similar to the role of pages in a paper-based lab-book, Genboree Project pages include links and summaries of previously run data sets, which include links to full results, such as 2D and 3D beta diversity plots, groups of alpha diversity plots, classification rates or relevant OTUs for classification.

## Results

We present two representative studies carried out using the Genboree Microbiome Toolset. The two studies in combination exercise all the steps outlined in Fig. 1. The first study describes how a Microbiome Toolset user may reproduce a previously reported analysis of alpha diversity in obese and lean twins [25] by carrying out analysis on publicly available data. The second study describes how the Toolset was used in a recently published study of the gut microbiota of children suffering from irritable bowel syndrome [26].

### Alpha diversity analysis using publicly available data

The Microbiome Toolset makes it easier to reproduce published results from publicly available data and to make new discoveries by performing meta-analyses of integrated data sets. As a proof of this capability, we set out to analyze data from a recently reported study of core gut microbiomes in obese and lean twins [25].

Our search of the Sequence Read Archive at NCBI using the query phrase ‘core gut microbiome in obese and lean twins’ yielded data associated with the twin study project SRP000319. We downloaded the experimental data for the V6 16S rRNA primer region (SRX001445), which contains 4 runs, 1.6M spots, and 205.6M bases. We were unable to obtain the MIDs from the SRA experiment XML. We were therefore precluded from de-multiplexing the original SFF files by the regular method, but we were able to find a work-around to solve this problem using de-multiplexed sequence data available on a supplemental data page from the Gordon Lab [27]. The metadata obtained from this exercise was compiled for use on the Microbiome Toolset.

**Figure 3:**
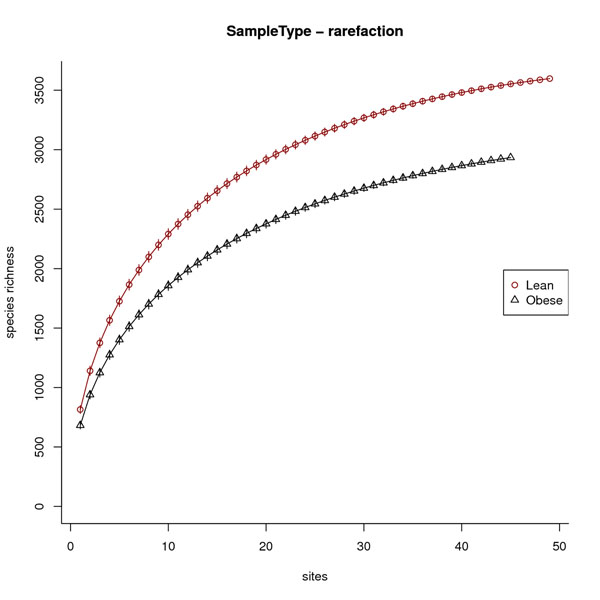
**Species richness analysis for lean twin and obese twin samples.** Species richness comparison between lean twin (n=49) and obese twin (n=45) samples. The lean twin cohort contains a higher degree of species richness as compared to the obese twin cohort.

**Figure 4:**
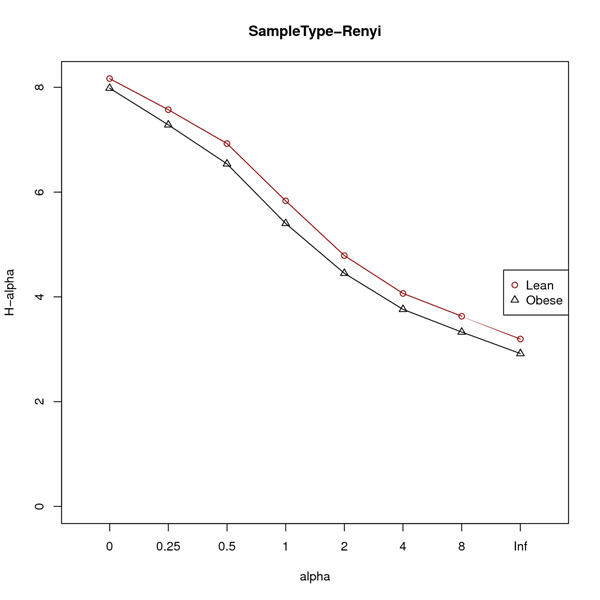
**Renyi diversity profiles for lean twin and obese twin samples**. Renyi diversity profiles for lean twin (n=50) and obese twin (n=50) samples. A non-intersecting line indicates that the obese twin cohort has a lower diversity as compared to the lean twin cohort.

Alpha diversity analysis started with sequences for 10 obese and 10 lean twin individuals (a total of 100 samples). 5 samples were removed from the obese cohort and 1 sample was removed from the lean cohort because they had less than 1,000 sequences per sample, leaving us with a total of 94 samples to evaluate alpha diversity. The analysis revealed that lean samples have higher V6 16S rRNA gene primer region alpha diversity both in terms of species richness (Fig. 3) and Renyi profile (Fig. 4), consistent with the original report by Turnbaugh, et al. [25].

### A study of microbiota in children with irritable bowel syndrome

Prior to this study [26], perturbations of the intestinal microbiota between healthy children and children with IBS were not well defined. The study therefore aimed to examine if any such perturbations could be detected. The gastrointestinal microbiota was analyzed in 22 children with IBS (69 samples) and 22 healthy children (71 samples) ages 7-12 for a total of 140 samples. The samples were analyzed for taxonomic abundance, beta diversity, phylogenetic analysis, and classification by supervised machine learning, as summarized in Fig. 1 and described in previous sections.

### Taxonomic abundance using the RDP pipeline

**Figure 5:**
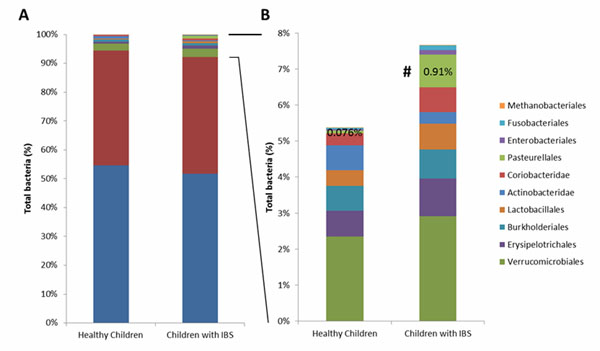
**Taxonomic abundance comparison between children with IBS and healthy children.** The pediatric gut microbiomes of children with IBS are characterized by greater abundance of Pasteurellales. Taxonomic classification was made using RDP classifier (Order) with 454 sequencing data. A) Percentage of all bacterial Orders represented. B) Percentage of bacterial taxa found in lower abundance (< 8% of total bacteria). Healthy children include 29 samples from 22 subjects, IBS patients include 42 samples from 22 patients (V1-V3 region). #: Significantly different between IBS and healthy children (P <.05).

Stacked bar charts for the taxonomically binned abundance data from the RDP pipeline (Fig. 5A) showed a high amount of similarities between the IBS and healthy gut microbiomes at the Order level. The majority of the sequences (> 90%) from both pooled samples were classified as Bacteroidales and Clostridiales. The average Bacteroides-to-Firmicutes ratio for the pooled IBS and healthy pediatric stool samples (data not shown) was similar to BMI averaged across individuals in a separate gut microbiome study [28]. Upon removal of Bacteroidales and Clostridiales from consideration, over-abundance of Pasterurellales in the IBS dataset became apparent (Fig. 5B).

### Phylogenetic analysis

**Figure 6:**
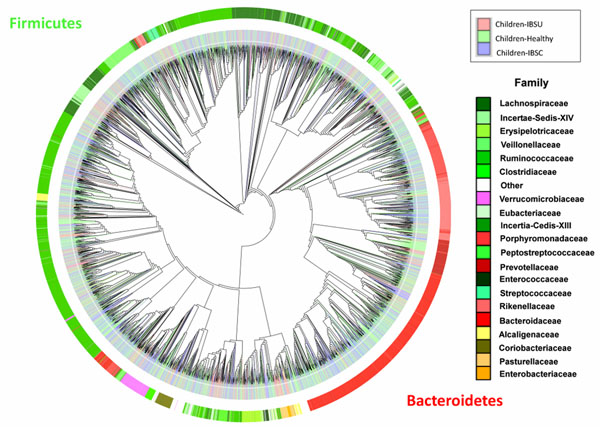
**Global phylogenetic tree comparing the intestinal microbiomes of healthy children, children with IBS-C, and children with IBS-U.** The phylogenetic tree was generated using QIIME and drawn with iTOL. Data comprised of the V1-V3 region for 22 healthy children (69 samples), 9 children with IBS-U (23 samples), and 13 children with IBS-C (42 samples). The map is colored by Phyla (exterior text), Patient Status (IBS-U - Light Red; IBS-C - Purple; Healthy - Light Green) and Family.

A phylogenetic tree (in Newick format) was produced by invoking the QIIME pipeline tool in the Microbiome Toolset, as described in previous sections. A visualization of the phylogenetic tree, along with the sample metadata input (Fig. 6) was produced using the Interactive Tree of Life (iTOL) [15] API from input generated by the Microbiome Toolset.

The phylogenetic tree (Fig. 6) does not visually reveal differences in phylogeny by the grouping of colors based on health within the inner ring, but it does shed some light on the phylogenetic relationship of the combined pediatric stool microbiome in terms of taxonomic membership (i.e. ratio of Bacteroidetes to Firmicutes) on the outer ring.

### Beta diversity analysis

**Figure 7:**
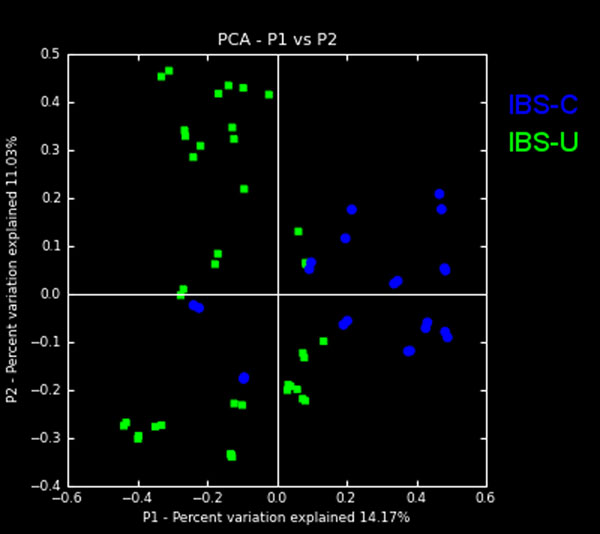
**Beta diversity analysis results for intestinal microbiomes of children with IBS-C and IBS-U.** The Principal Coordinates Analysis plot, using beta diversity analysis results and utilizing the Hellinger distance metric, shows that distal gut microbiomes of children segregate the IBS with constipation (IBS-C (blue), n=41 samples) and unsubtyped IBS (IBS-U (green), n=22 samples).

Beta diversity analysis was based on 454 pyrosequencing (V1-V3 region only, 2 replicates per samples). The Hellinger distance[29] was used to generate a matrix of pairwise sample dissimilarities between communities; a scatter plot (Fig. 7) was then generated from the matrix of distances using Principal Coordinates Analysis. The analysis yielded clustering corresponding to the IBS constipation (IBS-C) and IBS unsubtyped (IBS-U) cohorts when using the Hellinger distance metric. Although beta diversity plots are useful in qualitatively evaluating community similarities and differences, to gain further insight by detecting the features most likely to have caused the separation, we employed classification using supervised machine learning and feature selection, as discussed next.

### Classification using supervised machine learning and feature selection

RandomForest classification of the IBS-C and IBS-U subtypes was achieved with up to 98.5% success rate. The IBS-U group was distinguished by the presence and relative abundance of 70 OTUs, whereas the IBS-C group was identified by the presence and relative abundance of 54 OTUs (data not shown). Most of the OTUs that facilitated the classifications of these two IBS subtypes belong to the genera or groups such as *Bacteroides*, *Ruminococcus*, *Lachnospiraceae Incertae Sedis*, *Veillonella*, and *Erysipelotrichaceae*. We did not observe any extreme changes of relative abundances from these species groups, suggesting that the aggregate collections of species or strains (not individual species or strains) are likely the source of the high degree of classification.

**Figure 8:**
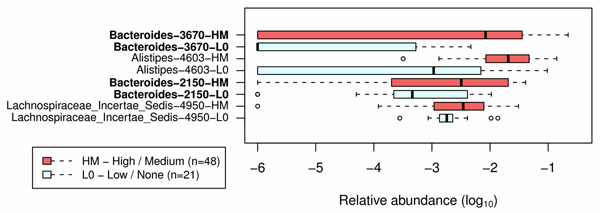
**Differential distribution of bacterial taxa that discriminate maximum pain levels in patients with recurrent abdominal pain.** The distribution of bacterial taxa in patients with recurrent abdominal pain was used to classify the subjects based on the maximum levels of abdominal pain. Bacterial taxa (specified in leftmost column) were analyzed using randomForest and confirmed by feature selection using Boruta. The list is sorted first by Mann-Whitney U score followed by the largest disparity in medians for each group. Taxa represent the lowest taxonomic depth (Genus) that is labeled by RDP Classifier (at ≥ 80% bootstrap cut off). The degree of abdominal pain was differentiated by the maximum level of pain recorded during a 14-day period. Red rectangles display the HM (high- medium level) maximum abdominal pain phenotype. Light blue rectangles display the L0 (low-zero level) maximum abdominal pain phenotype. Boxes represent the first quartile, median, and third quartile of the distribution of OTUs for each pain group. Empty circles represent outliers that are 1.5x greater than the respective interquartile ranges. Shown are OTUs with increased levels of maximum pain in children with HM versus L0 maximum abdominal pain phenotypes.

Maximum abdominal pain levels were analyzed in children with IBS and were classified by the maximum pain levels during a 14-day period. High and medium (HM) pain groups were classified by a maximum pain level of 4 or more, whereas low and zero (L0) pain groups represented a maximum pain level of 3 or less. Abundance of taxa representing the lowest taxonomic depth (Genus) that is labeled by RDP Classifier (at ≥ 80% bootstrap cut off) was used for classification of the two groups. As illustrated in Fig. 8, children within the HM pain phenotype contained 4 OTUs within the following Genera: *Bacteroides*, *Alistipes*, and *Lachonospiraceae Incertae Sedis*.

## Discussion

As demonstrated by the two key use case scenarios, the Genboree Microbiome Toolset provides valuable insights into microbiome diversity and identifies disease-associated operational taxonomic units. The Toolset empowers investigators (notably translational researchers) to carry out a wide range of analyses including alpha diversity and beta diversity, phylogenetic profile analysis, classification by supervised machine learning, and feature selection. By lowering the barrier to performing a comprehensive set of microbiome analyses, the Toolset enables characterization of microbiomes and the discovery of disease-associated perturbations.

The Toolset is exposed using the Software-as-a-Service model via the Genboree Workbench. In addition to interactive use, all the functionality of the Workbench is also available programmatically using the Genboree REST Application Programming Interfaces (REST APIs) for web-based integration into project-specific pipelines. The Microbiome Toolset therefore provides a web-based programming environment for bioinformaticians in which to conduct more advanced or custom microbiome analyses.

We foresee the Microbiome Integrated Toolset evolving in multiple directions. First, based on user feedback and progress in the field, we plan to extend and add new pipelines for 16S rRNA genic analysis. Second, the toolset will be extended to enable analyses based on whole-metagenome sequencing. To achieve this aim and accommodate rapidly increasing sequencing volumes, the Genboree Workbench is designed to seamlessly access cloud computing resources across the web.

## Competing interests

AM founded and owns shares in IP Genesis, Inc., a corporation which owns an exclusive license from Baylor College of Medicine for commercial use of the Genboree trademark.

## Software availability and requirements

The Genboree Microbiome Toolset is part of the Genboree Workbench and can be accessed at the address http://genboree.org/java-bin/workbench.jsp. Supported browsers are Internet Explorer versions 8 and above, Mozilla Firefox versions 7 and above. A tutorial for the Genboree Microbiome Toolset is available as Additional File 1. Additional information can be found at the address http://genboree.org/microbiome.

## Authors' contributions

KR, CC, ARJ, JV, KA, and AM conceived the Genboree Microbiome Toolset and co-wrote the manuscript. KR, JM, and CC deployed and evaluated microbiome analysis tools. AM and ARJ conceived the Genboree Workbench and Genboree REST APIs. ARJ, SP, AT, and SR implemented the Genboree Workbench and integrated the Genboree Microbiome Toolset via the Genboree REST APIs. TAM, DS, SR, MAD, RS, KA, and JV performed microbial data collection and analysis. AM supervised the Genboree project.

## Supplementary Material

Additional file 1 **Tutorial for the Genboree Microbiome Toolset** The attached file contains a tutorial for the Genboree Microbiome Toolset. Click here for file
