# Supplementary material for: The Genboree Microbiome Toolset and the analysis of 16S rRNA microbial sequences
Source: BMC Bioinformatics. 2012 Aug 24;13(Suppl 13):S11. doi: 10.1186/1471-2105-13-S13-S11 (PMC3426808; doi:10.1186/1471-2105-13-S13-S11)
Supplement: Additional file 1 — Tutorial for the Genboree Microbiome Toolset The attached file contains a tutorial for the Genboree Microbiome Toolset. [file 1471-2105-13-S13-S11-S1.pdf]

Genboree Microbiome Toolset - Tutorial

Previous Tutorials

- September_2011_GMT-Tutorial_Single-Samples

We will be going through a tutorial on the Genboree Microbiome Toolset with publicly available data:

- Sample Meta Data
  - tutorial_meta_data.tsv
    - tutorial_meta_data.tsv
- Sequence Files
  - tutorial_sequence_files.sff.gz
    - tutorial_sequence_files.sff.gz

Create Sample Meta Data

The first step towards completing work on the Genboree Microbiome Workbench is to produce the sample meta data. The sample meta data reflects the attributes of each sample (i.e. health, body site, BMI, etc.) as well as the necessary information required to extract the sequence data from the original SFF or SRA sequence file.

Requirements:

- Tab-delimited
- The first line of the file contains the column headers, as a comment-line. It must start with a '#'.
- One of the fields MUST be 'name' which should be unique for all Sample records.
- All records MUST have the same number of fields/columns.
- Fields:
  - name - [Required] Unique name associated with the Sample.
  - barcode - [Required] The Sample-specific sequence used to barcode the sequences in multiplex sequencing. Will be used to identify which sequence records go with which Samples.
  - region - [optional] The name of the 16S region amplified. Defaults to V3V5 if no 'region', 'proximal', or 'distal' primer is included. The proximal and distal primer pair should amplify the region mentioned here.
  - proximal - [optional] The upstream primer used to amplify the microbial 16S rRNA region. If not provided, then a standard primer pair will be looked up based on the 'region' column. For example, if the user does not know the proximal primer, they can list V3V5 in the 'region' column and the stored primer used to amplify the V3V5 region is assumed; the upstream primer in that case is CCGTCAATTCMTTTRAGT.
  - distal - [optional] The downstream primer used to amplify the microbial 16S rRNA region. If not provided, then a standard primer pair used looked up based on the 'region' column. For example, if the user does not know the distal primer, they can list V3V5 in the 'region' column and the stored primer used to amplify the V3V5 region is assumed; the upstream primer in that case is CTGCTGCCTCCCGTAGG.
- Also, please avoid any spaces or any other character other than a-zA-Z0-9-_

Sample meta data

- 10 samples
- 2 body sites
  - Stool
  - Throat
- 1 primer region
  - V3V5

#name	barcode	proximal	distal	region	body_site
S_700033665	CCGTTCCTC	CCGTCAATTCMTTTRAGT	CTGCTGCCTCCCGTAGG	V3V5	Stool
S_700035861	ACCGGCGTTC	CCGTCAATTCMTTTRAGT	CTGCTGCCTCCCGTAGG	V3V5	Stool
S_700095543	ACGAATTAAC	CCGTCAATTCMTTTRAGT	CTGCTGCCTCCCGTAGG	V3V5	Stool
S_700095850	AACCGGATAC	CCGTCAATTCMTTTRAGT	CTGCTGCCTCCCGTAGG	V3V5	Stool
S_700101600	AACGGAACGC	CCGTCAATTCMTTTRAGT	CTGCTGCCTCCCGTAGG	V3V5	Stool
T_700016994	AATAACCGTC	CCGTCAATTCMTTTRAGT	CTGCTGCCTCCCGTAGG	V3V5	Throat
T_700095565	TTAATGGAAC	CCGTCAATTCMTTTRAGT	CTGCTGCCTCCCGTAGG	V3V5	Throat

T_700095872	CGGACCGGAAC	CCGTCAATTCMTTTRAGT	CTGCTGCCTCCCGTAGG	V3V5	Throat
T_700101388	CCGAACGAC	CCGTCAATTCMTTTRAGT	CTGCTGCCTCCCGTAGG	V3V5	Throat
T_700101622	TTCGTTCTTC	CCGTCAATTCMTTTRAGT	CTGCTGCCTCCCGTAGG	V3V5	Throat

Create Group

- Login or create an account on http://www.genboree.org
- Click the *Groups* tab
- Click the *Create* tab
- Enter a Name for the Group (i.e. `GMT_Tutorial`)
- Optionally enter a description
- Click the 'Create' button

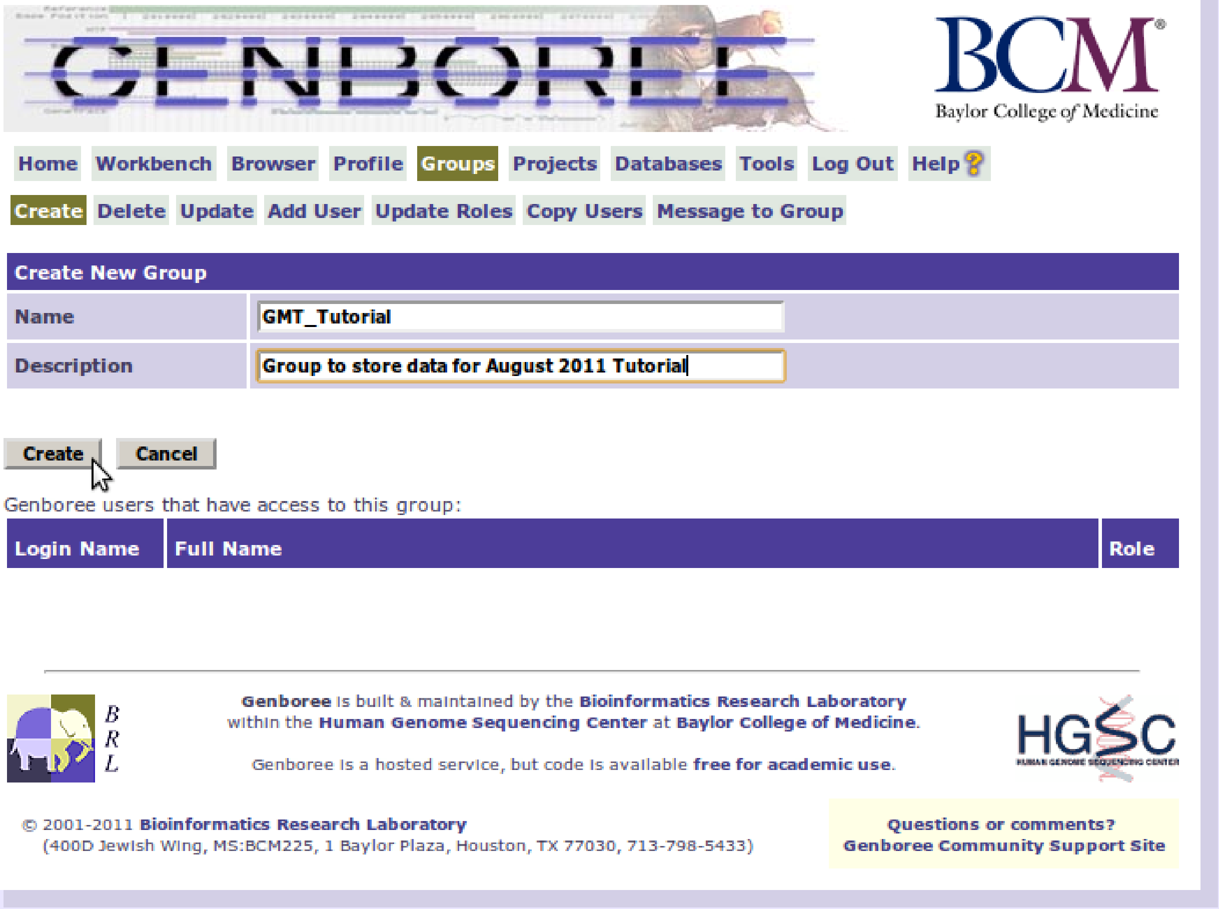

Create Database

- Click the *Databases* tab
- Select your newly created Group `GMT_Tutorial`
- Click *Create* tab
- Enter your Database Name (i.e. `gmtDB`)
- Click 'Create' button

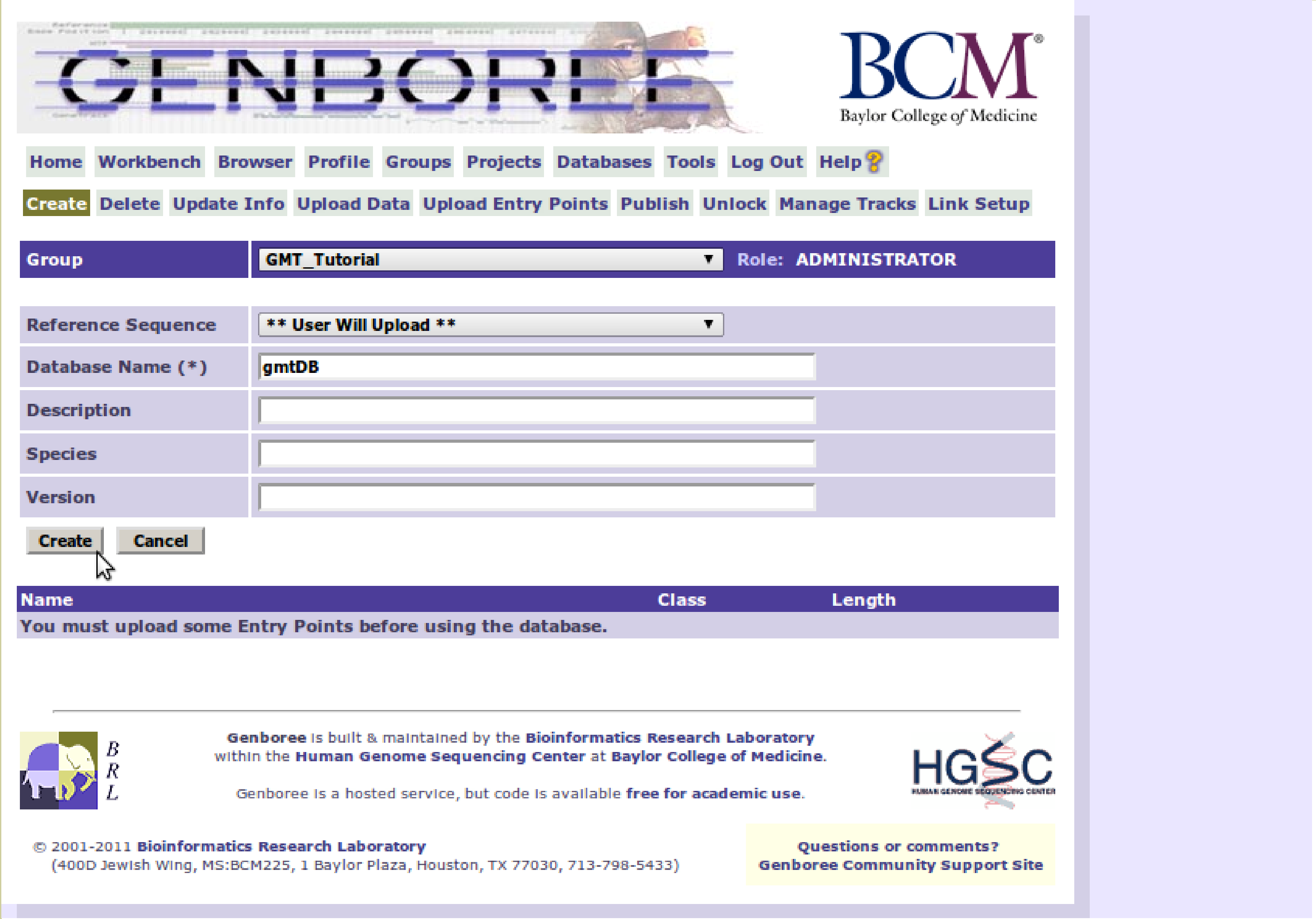

Create Project

- Click the *Projects* tab
- Select your newly created Group `GMT_Tutorial`
- Click the *Create* tab
- Enter your New Project Name (i.e. `gmtProject`)
- Click the 'Create' Button

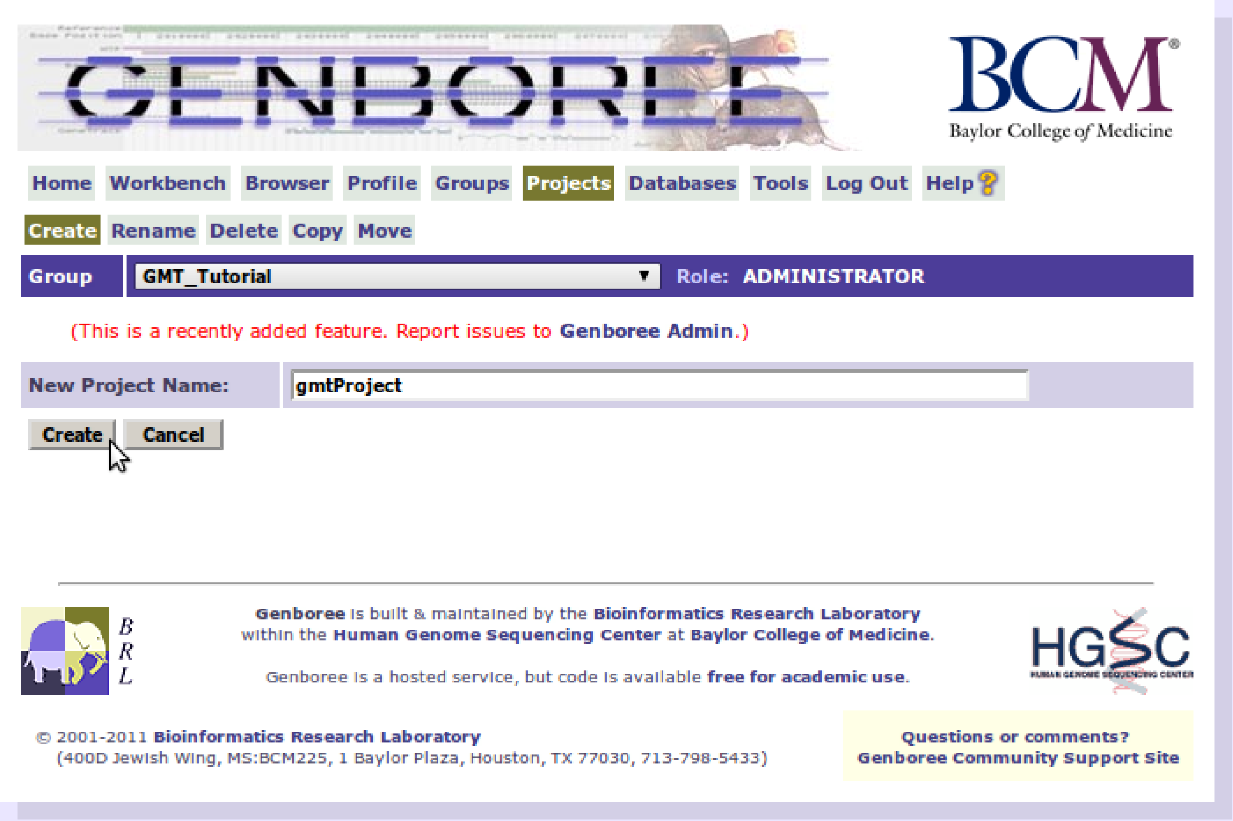

Upload Files

- Click the *Workbench* tab
- Within the *Data Selector* window expand the `Groups` -> `GMT_Tutorial` -> `Databases` -> `gmtDB`
- Drag the `gmtDB` database into the *Output Targets* window
- Click the *Data* tab, the *Files* tab, and then the *Transfer File* tab
- Browse to the location of `tutorial_meta_data.tsv`
- Click 'Submit'
- Click the *Data* tab, the *Files* tab, and then the *Transfer File* tab
- Browse to the location of `tutorial_sequence_files.tar.gz`
- Check 'Unpack Multi-File Archive'
- Click 'Submit'

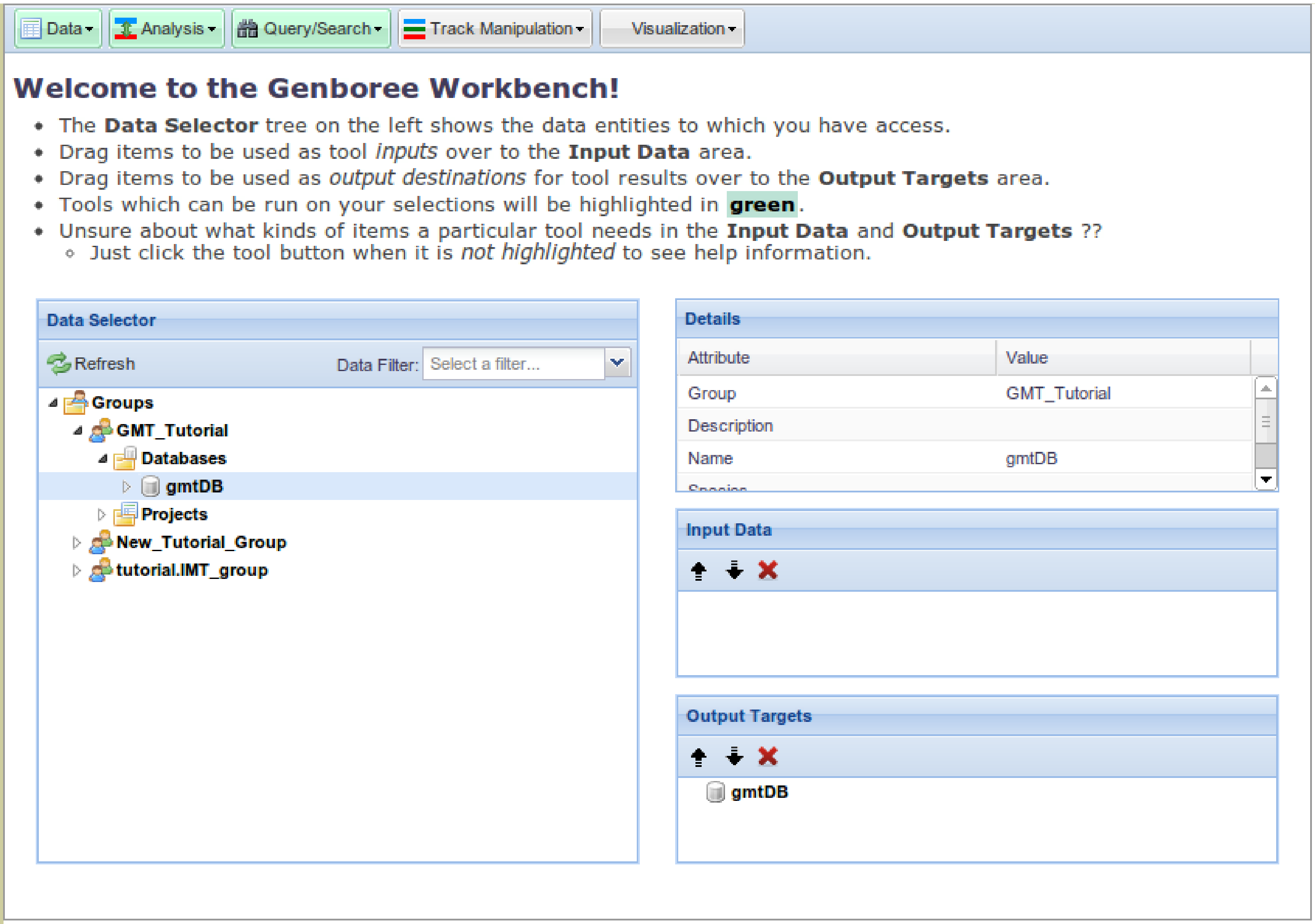
Data
Analysis
Query/Search
Track Manipulation
Visualization
Welcome to the Genboree Workbench!
The Data Selector tree on the left shows the data entities to which you have access.
Drag items to be used as tool inputs over to the Input Data area.
Drag items to be used as output destinations for tool results over to the Output Targets area.
Tools which can be run on your selections will be highlighted in green.
Unsure about what kinds of items a particular tool needs in the Input Data and Output Targets ??
Just click the tool button when it is not highlighted to see help information.
Data Selector
Refresh
Data Filter: Select a filter...
Groups
GMT_Tutorial
Databases
gmtDB
Projects
New_Tutorial_Group
tutorial.IMT_group
Details
Attribute
Value
Group
GMT_Tutorial
Description
Name
gmtDB
Input Data
Output Targets
gmtDB

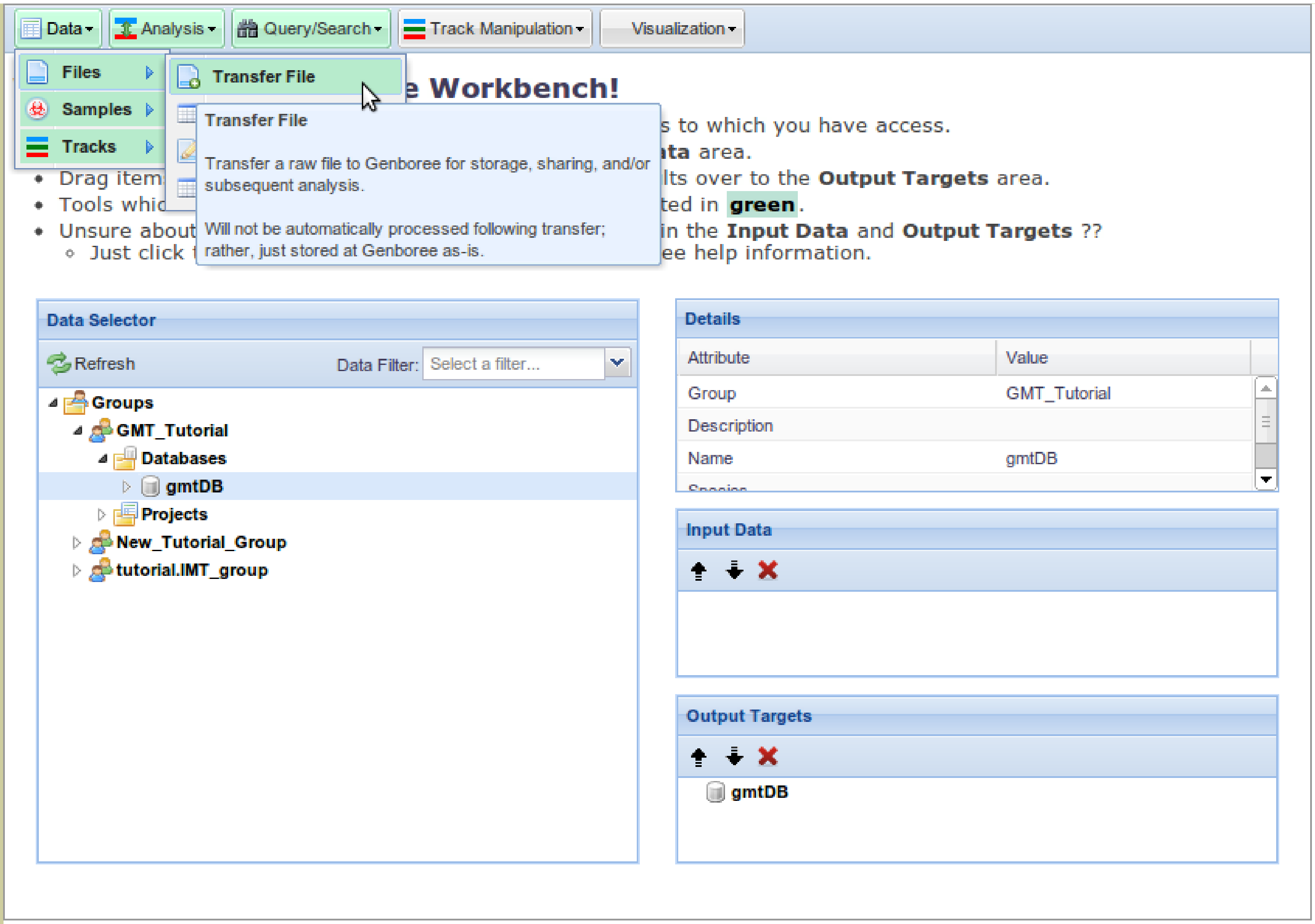
Data
Analysis
Query/Search
Track Manipulation
Visualization
Files
Samples
Tracks
Transfer File
e Workbench!
Transfer File
Transfer a raw file to Genboree for storage, sharing, and/or subsequent analysis.
Will not be automatically processed following transfer; rather, just stored at Genboree as-is.
s to which you have access.
ata area.
Drag item
lts over to the Output Targets area.
Tools whic
ted in green.
Unsure about
in the Input Data and Output Targets ??
Just click t
ee help information.
Data Selector
Refresh
Data Filter:
Select a filter...
Groups
GMT_Tutorial
Databases
gmtDB
Projects
New_Tutorial_Group
tutorial.IMT_group
Details
Attribute
Value
Group
GMT_Tutorial
Description
Name
gmtDB
Input Data
Output Targets
gmtDB

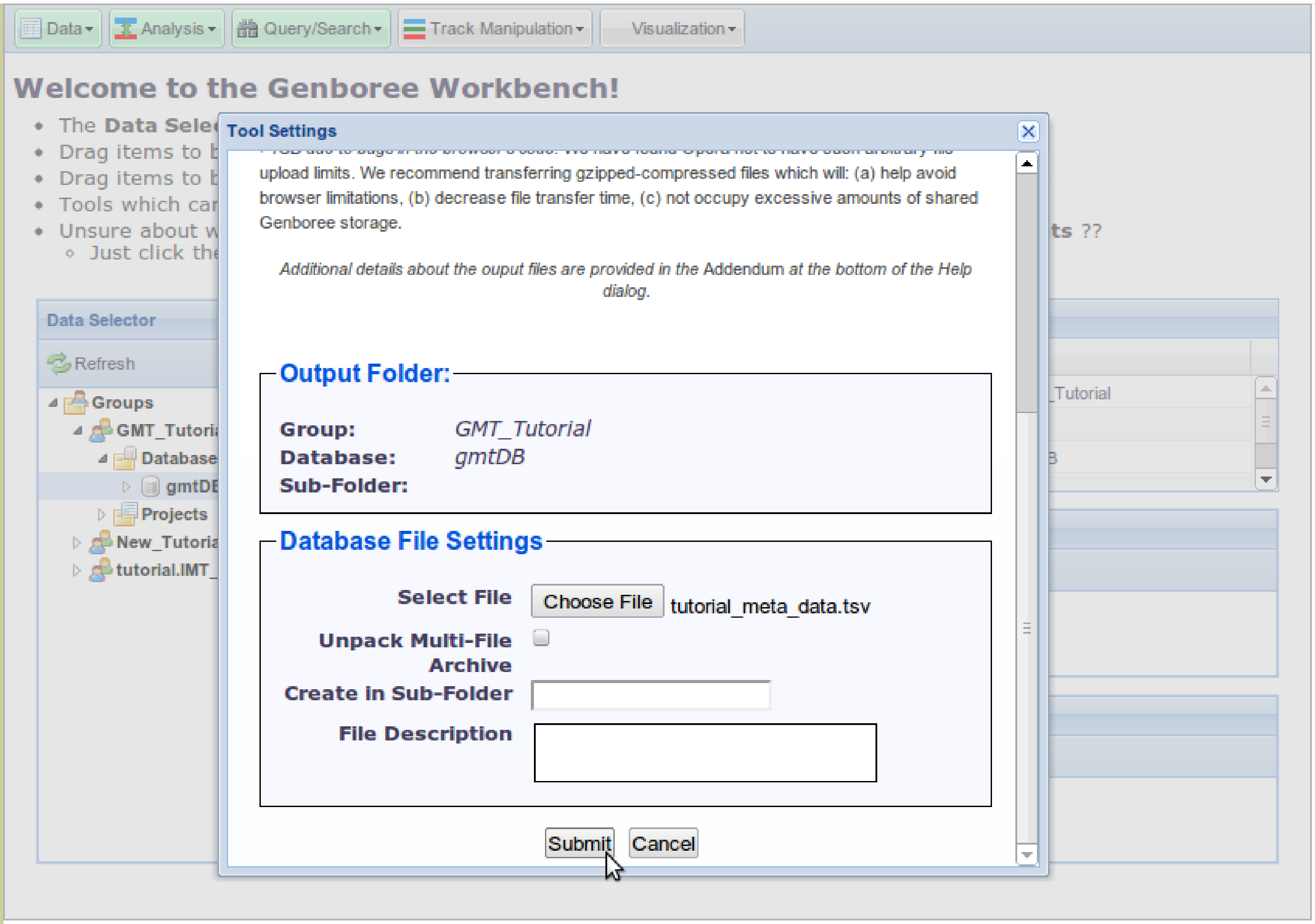
Data
Analysis
Query/Search
Track Manipulation
Visualization
Welcome to the Genboree Workbench!
Tool Settings
upload limits. We recommend transferring gzipped-compressed files which will: (a) help avoid browser limitations, (b) decrease file transfer time, (c) not occupy excessive amounts of shared Genboree storage.
Additional details about the ouput files are provided in the Addendum at the bottom of the Help dialog.
Output Folder:
Group: GMT_Tutorial
Database: gmtDB
Sub-Folder:
Database File Settings
Select File
Choose File
tutorial_meta_data.tsv
Unpack Multi-File Archive
Create in Sub-Folder
File Description
Submit
Cancel
Data Selector
Refresh
Groups
Projects

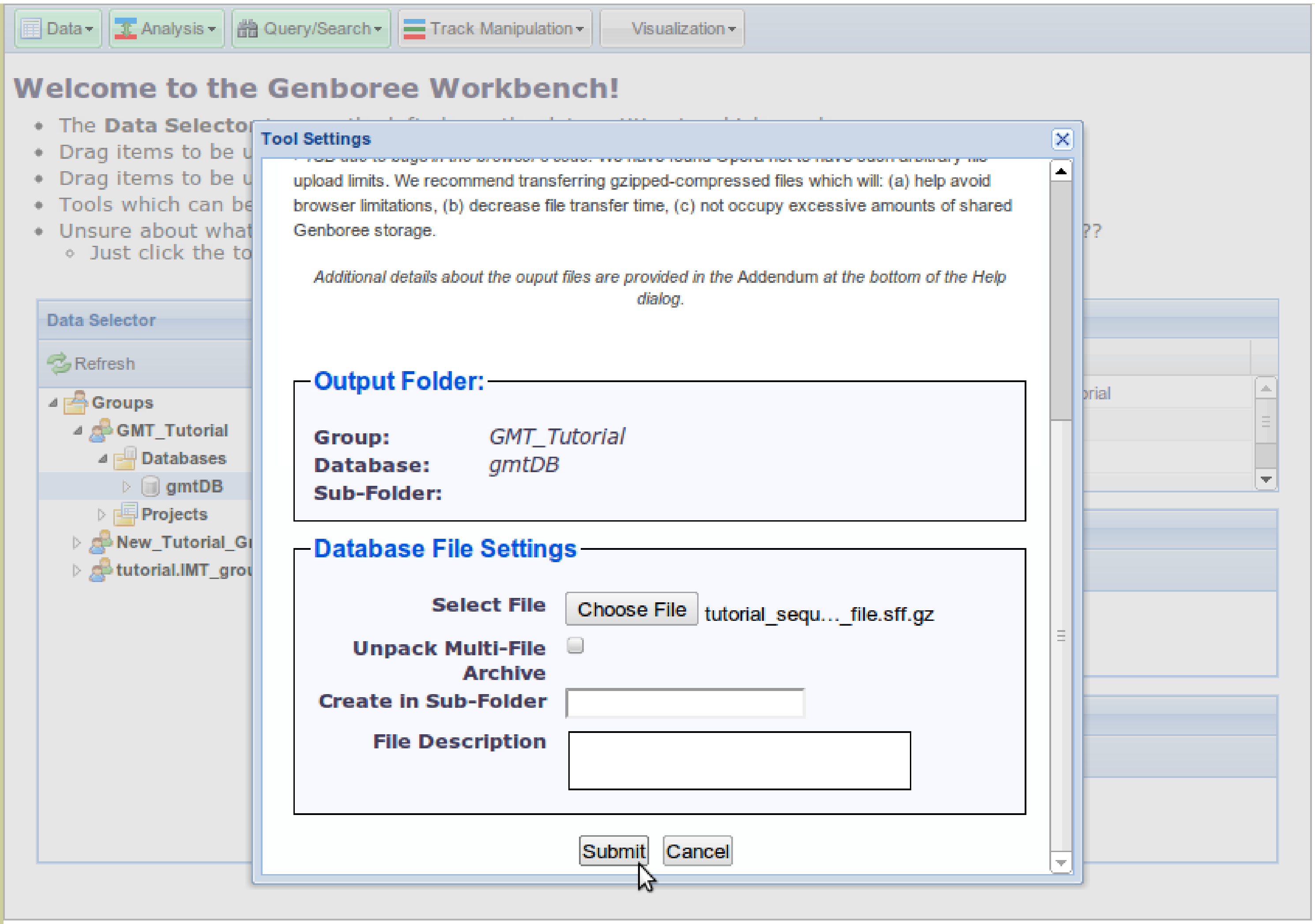
Data
Analysis
Query/Search
Track Manipulation
Visualization
Welcome to the Genboree Workbench!
Tool Settings
upload limits. We recommend transferring gzipped-compressed files which will: (a) help avoid browser limitations, (b) decrease file transfer time, (c) not occupy excessive amounts of shared Genboree storage.
Additional details about the ouput files are provided in the Addendum at the bottom of the Help dialog.
Output Folder:
Group: GMT_Tutorial
Database: gmtDB
Sub-Folder:
Database File Settings
Select File
Choose File
tutorial_sequ…_file.sff.gz
Unpack Multi-File Archive
Create in Sub-Folder
File Description
Submit
Cancel
Data Selector
Refresh
Groups
GMT_Tutorial
Databases
gmtDB
Projects

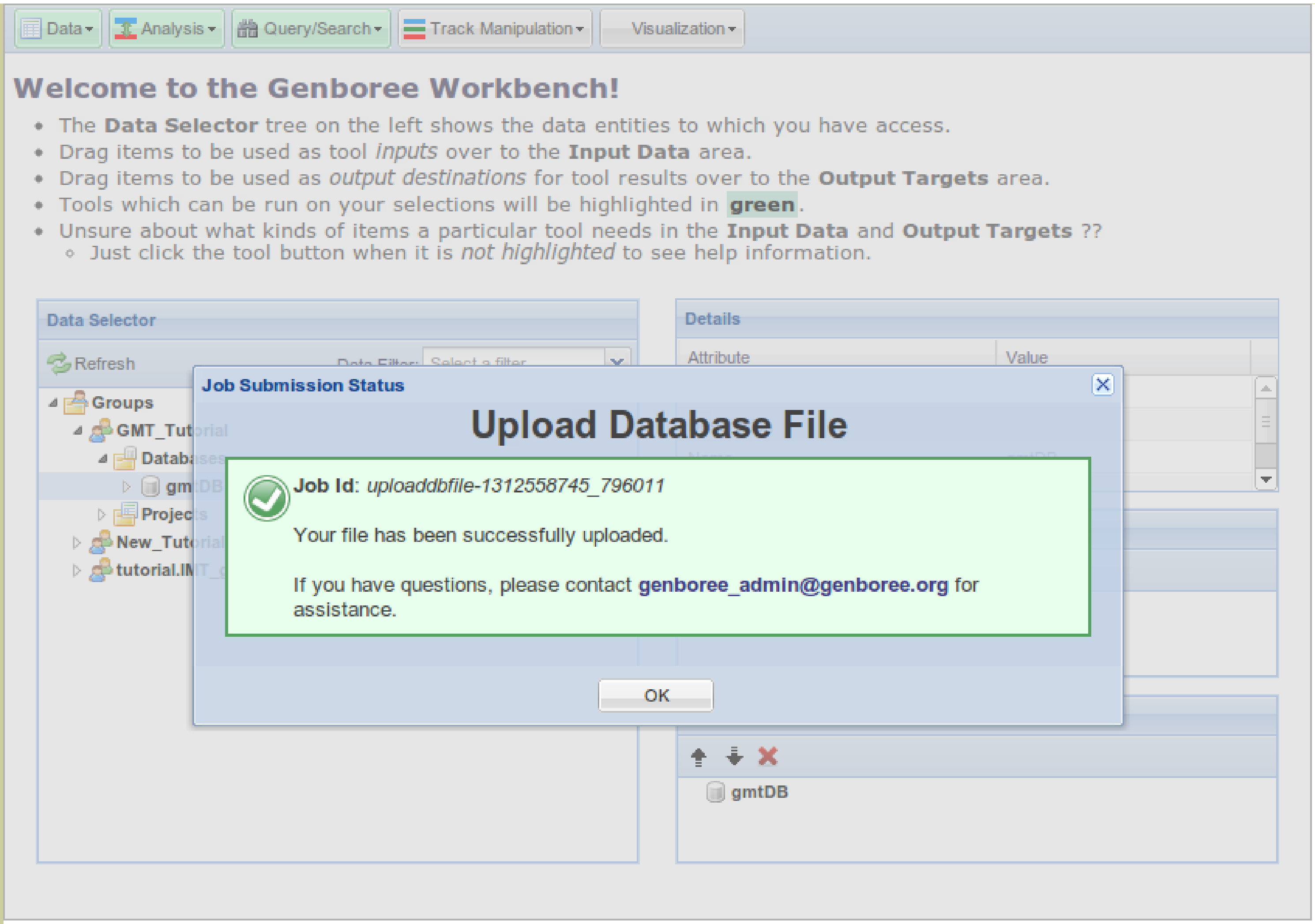

View Uploaded Files

- Click the ***Refresh*** button in the ***Data Selector*** window
- **Expand** `Groups` **->** `GMT_Tutorial` **->** `Databases` **->** `gmtDB` **->** `Files` **to see that your files have been uploaded and decompressed from the multi-file archive**

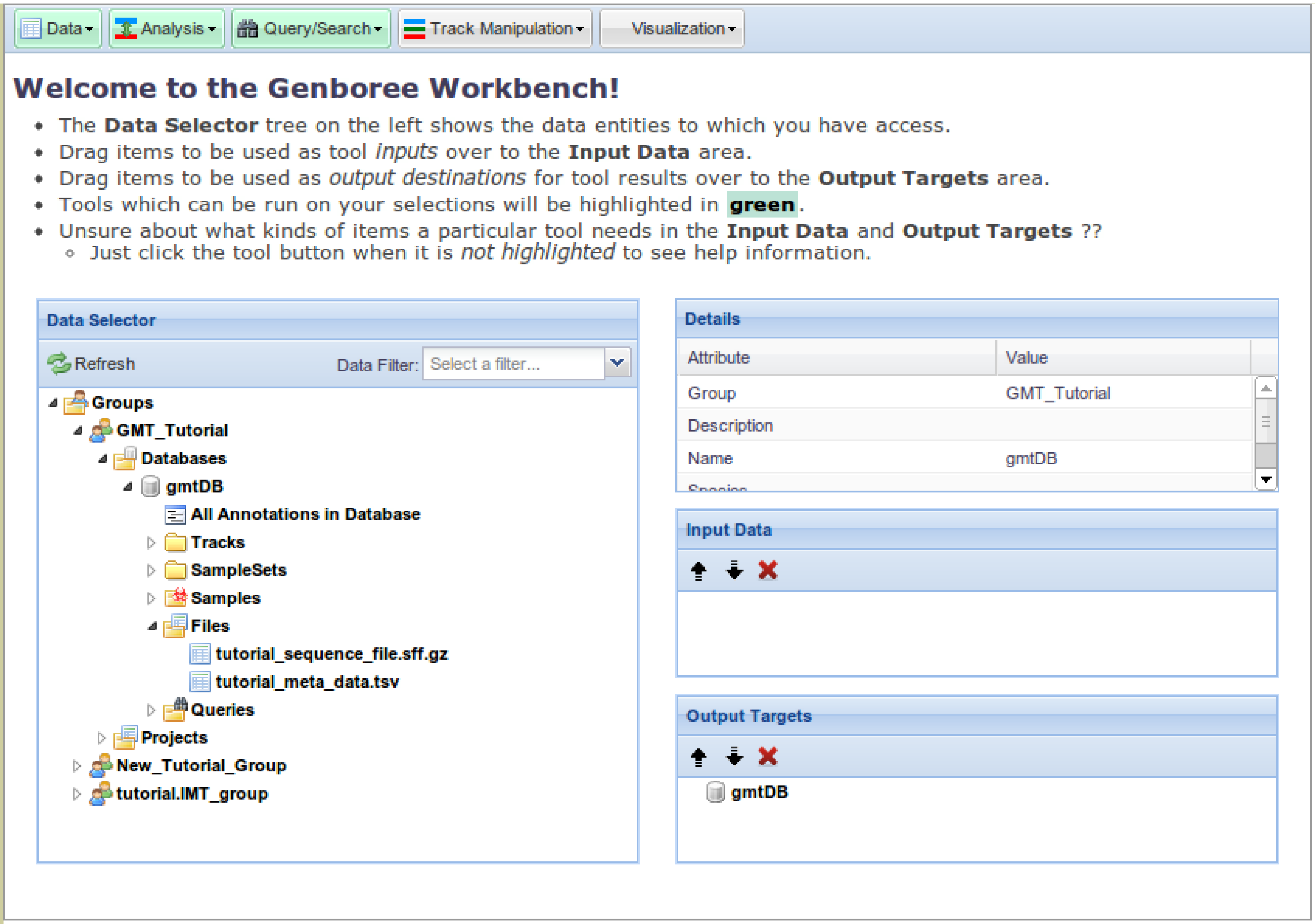

Import Samples

- Drag over the `tutorial_meta_data.tsv` file from the *Data Selector* window to the *Input Data* window
- Drag over the `gmtDB` database from the *Data Selector* window to the *Output Targets* window
- Click the *Data* tab, the *Samples* tab, and finally the *Import Samples* tab
- Create a new sample set by entering "tutorial_sample_set" into the 'Assign Samples to new Sample Set'
- Click the 'Submit' button
- Wait for confirmation email

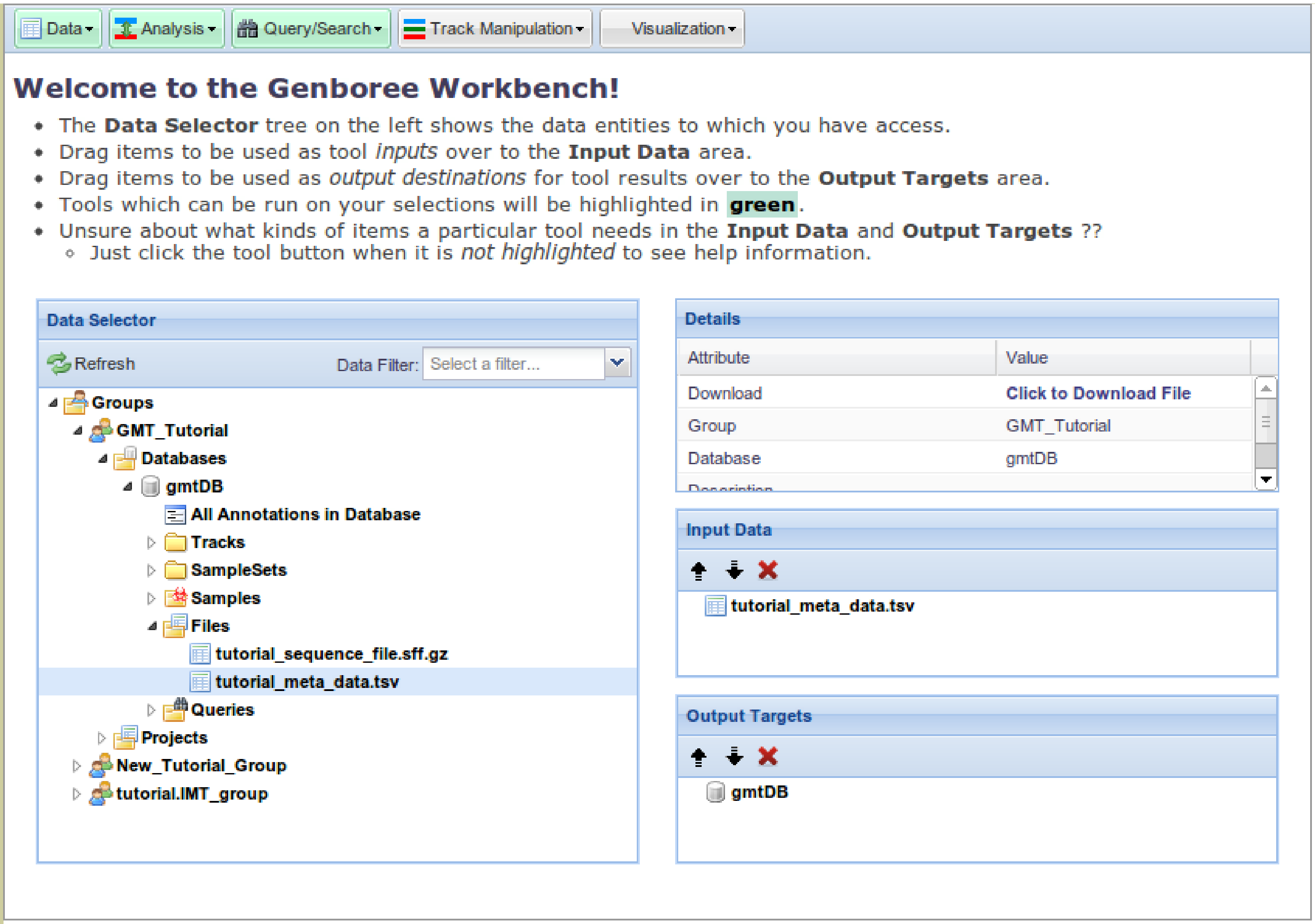

Data
Analysis
Query/Search
Track Manipulation
Visualization
Welcome to the Genboree Workbench!
The Data Selector tree on the left shows the data entities to which you have access.
Drag items to be used as tool inputs over to the Input Data area.
Drag items to be used as output destinations for tool results over to the Output Targets area.
Tools which can be run on your selections will be highlighted in green.
Unsure about what kinds of items a particular tool needs in the Input Data and Output Targets ??
Just click the tool button when it is not highlighted to see help information.
Data Selector
Refresh
Data Filter: Select a filter...
Groups
GMT_Tutorial
Databases
gmtDB
All Annotations in Database
Tracks
SampleSets
Samples
Files
tutorial_sequence_file.sff.gz
tutorial_meta_data.tsv
Queries
Projects
New_Tutorial_Group
tutorial.IMT_group
Details
Attribute
Value
Download
Click to Download File
Group
GMT_Tutorial
Database
gmtDB
Input Data
tutorial_meta_data.tsv
Output Targets
gmtDB

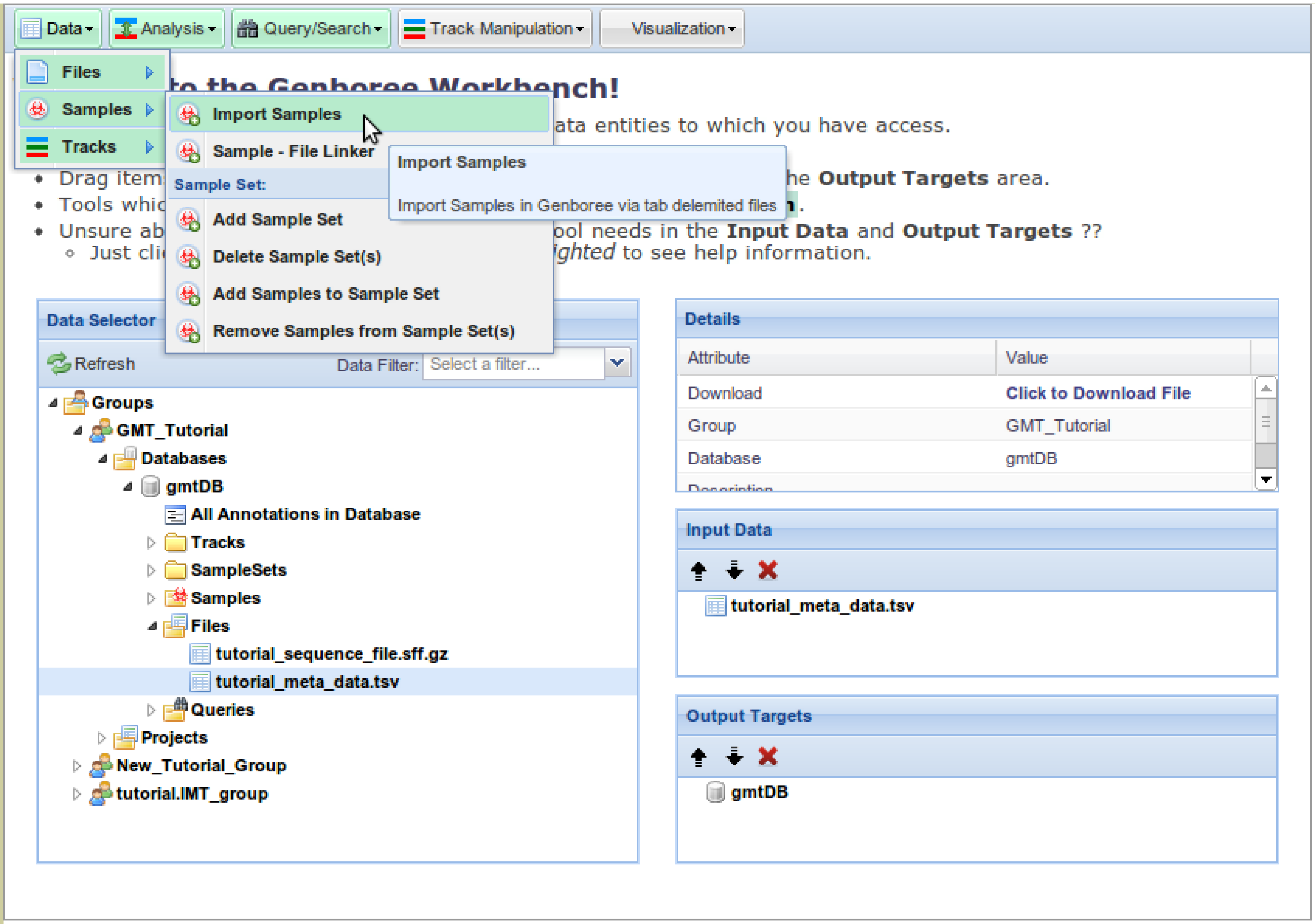

Data
Analysis
Query/Search
Track Manipulation
Visualization
Files
Samples
Tracks
Import Samples
Sample - File Linker
Sample Set:
Add Sample Set
Delete Sample Set(s)
Add Samples to Sample Set
Remove Samples from Sample Set(s)
Import Samples
Import Samples in Genboree via tab delemited files
Data Selector
Refresh
Data Filter:
Select a filter...
Groups
GMT_Tutorial
Databases
gmtDB
All Annotations in Database
Tracks
SampleSets
Samples
Files
tutorial_sequence_file.sff.gz
tutorial_meta_data.tsv
Queries
Projects
New_Tutorial_Group
tutorial.IMT_group
Details
Attribute
Value
Download
Click to Download File
Group
GMT_Tutorial
Database
gmtDB
Input Data
tutorial_meta_data.tsv
Output Targets
gmtDB

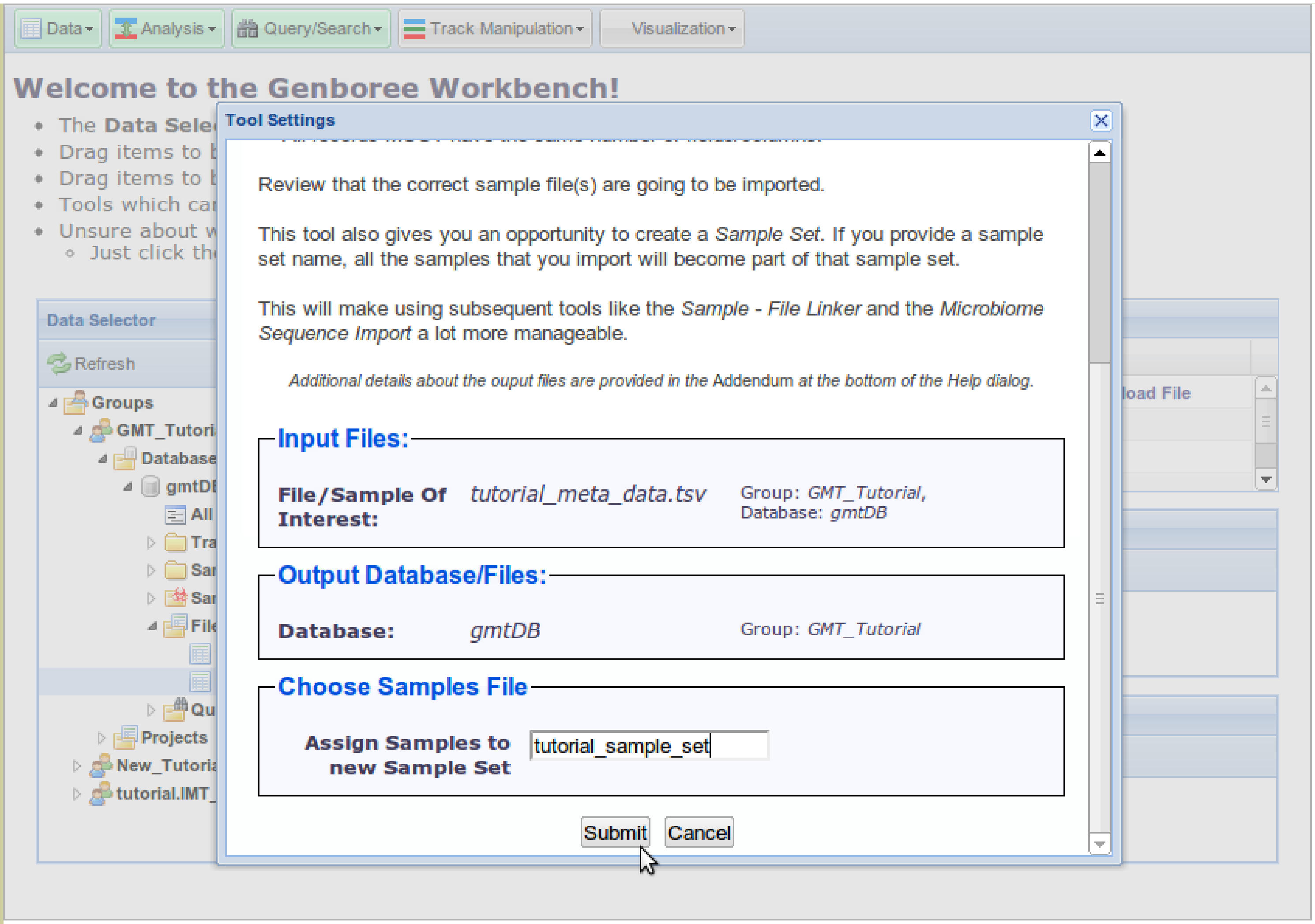


```
Hello Tutorial IMT,

Your Samples Importer job has completed successfully.

JOB SUMMARY:
 JobID          : wbJob-samplesimporter-1312569590_101768
 File Name      : tutorial_meta_data.tsv

The following file(s) has been uploaded as samples:
 tutorial_meta_data.tsv

The Genboree Team
```

View Imported Samples

- **Click the *Refresh* button in the *Data Selector* window**
- **Expand `Groups` -> `GMT_Tutorial` -> `Databases` -> `gmtDB` -> `Samples` to see that your samples have been uploaded**

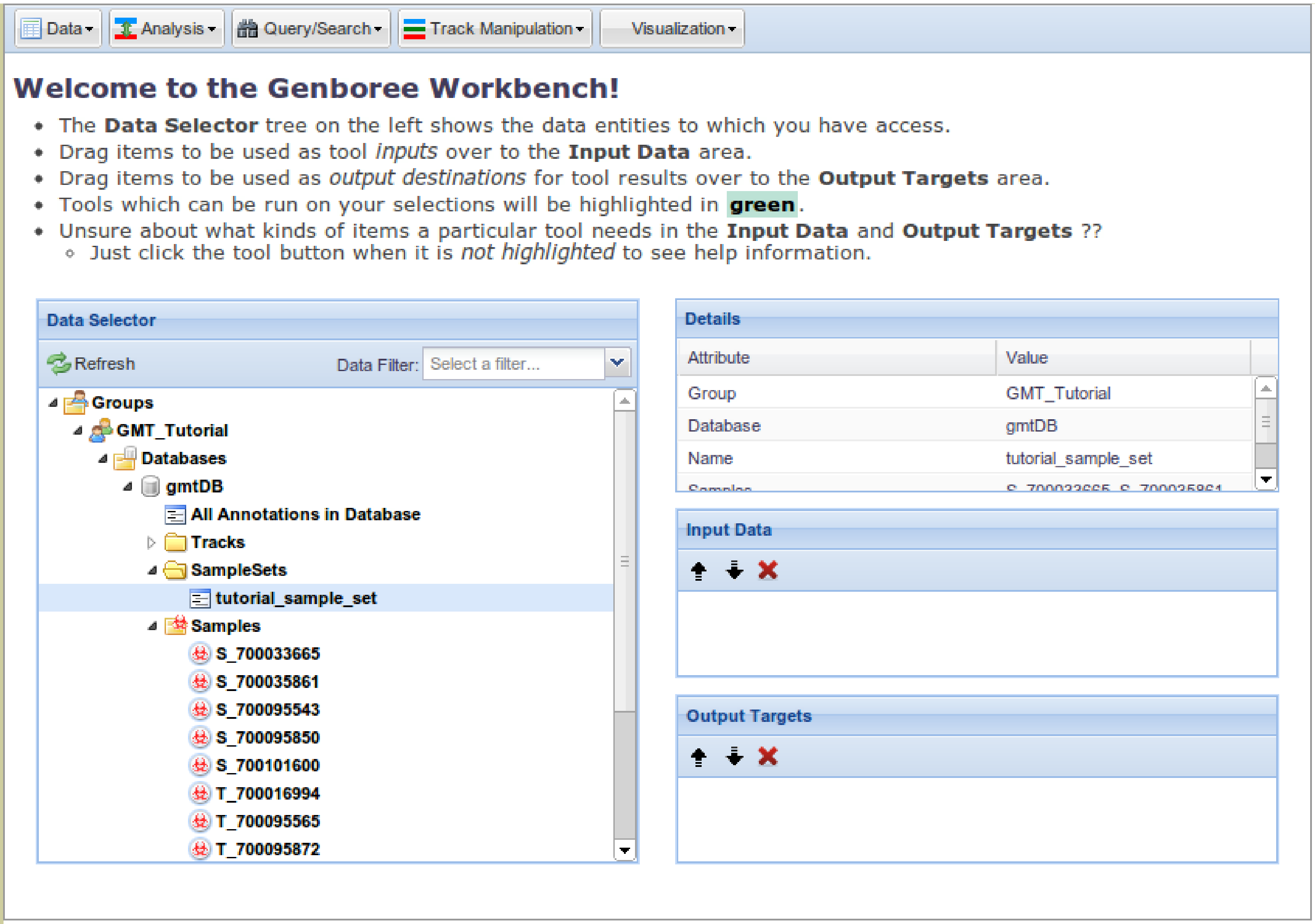

Link Samples To Sequence Files

- Remove any items from the *Input Data* window by selecting the items and clicking the red X
- Remove any items from the *Output Targets* window by selecting the items and clicking the red X
- Expand the `Groups -> GMT_Tutorial -> Databases -> gmtDB -> Files`
  - Drag the `tutorial_sequence_file.sff.gz` file from the *Data Selector* window to the *Input Data* window
- Expand the `Groups -> GMT_Tutorial -> Databases -> gmtDB -> SampleSets`
  - Drag the `tutorial_sample_set` from the *Data Selector* window to the *Input Data* window below the `tutorial_sequence_file.sff.gz` entry
    - Note: Make sure that the sequence file is always followed by the sample, sample set, or sample folder that is to be linked. You can do this for multiple data sets, just make sure it is always sequence file followed by sample data, sequence file followed by sample data, etc.

- Click the *Data* tab, the *Samples* tab, and finally the *Sample - File Linker* tab
- Verify that you have correctly ordered your SFF/SRA files followed by the appropriate Samples and click the 'Submit' button
- Wait for the confirmation email

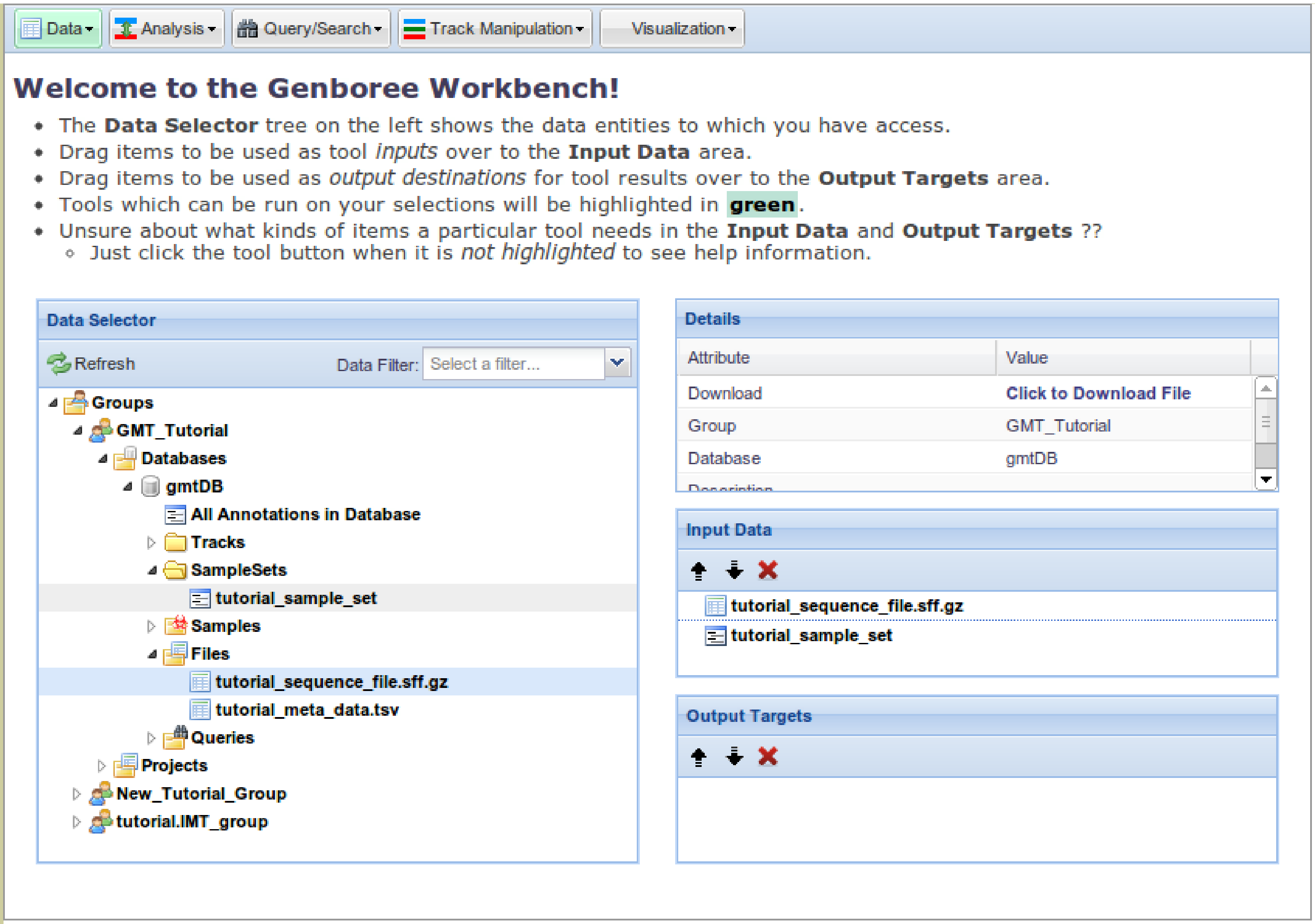
Data
Analysis
Query/Search
Track Manipulation
Visualization
Welcome to the Genboree Workbench!
The Data Selector tree on the left shows the data entities to which you have access.
Drag items to be used as tool inputs over to the Input Data area.
Drag items to be used as output destinations for tool results over to the Output Targets area.
Tools which can be run on your selections will be highlighted in green.
Unsure about what kinds of items a particular tool needs in the Input Data and Output Targets ??
Just click the tool button when it is not highlighted to see help information.
Data Selector
Refresh
Data Filter:
Select a filter...
Groups
GMT_Tutorial
Databases
gmtDB
All Annotations in Database
Tracks
SampleSets
tutorial_sample_set
Samples
Files
tutorial_sequence_file.sff.gz
tutorial_meta_data.tsv
Queries
Projects
New_Tutorial_Group
tutorial.IMT_group
Details
Attribute
Value
Download
Click to Download File
Group
GMT_Tutorial
Database
gmtDB
Input Data
tutorial_sequence_file.sff.gz
tutorial_sample_set
Output Targets

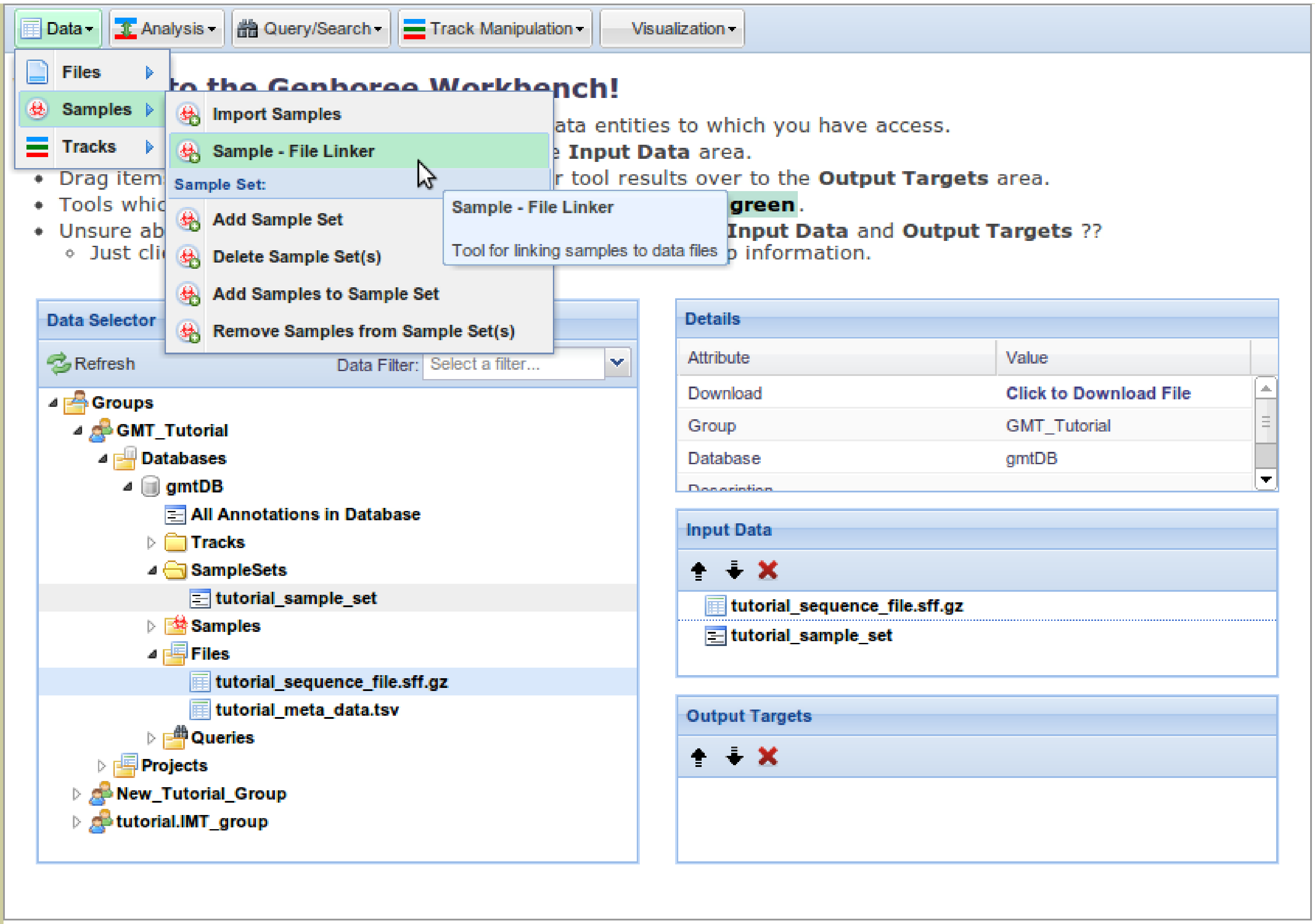

Data
Analysis
Query/Search
Track Manipulation
Visualization
Files
Samples
Tracks
Import Samples
Sample - File Linker
Sample Set:
Add Sample Set
Delete Sample Set(s)
Add Samples to Sample Set
Remove Samples from Sample Set(s)
Sample - File Linker
Tool for linking samples to data files
Data Selector
Refresh
Data Filter:
Select a filter...
Groups
GMT_Tutorial
Databases
gmtDB
All Annotations in Database
Tracks
SampleSets
tutorial_sample_set
Samples
Files
tutorial_sequence_file.sff.gz
tutorial_meta_data.tsv
Queries
Projects
New_Tutorial_Group
tutorial.IMT_group
Details
Attribute
Value
Download
Click to Download File
Group
GMT_Tutorial
Database
gmtDB
Input Data
tutorial_sequence_file.sff.gz
tutorial_sample_set
Output Targets

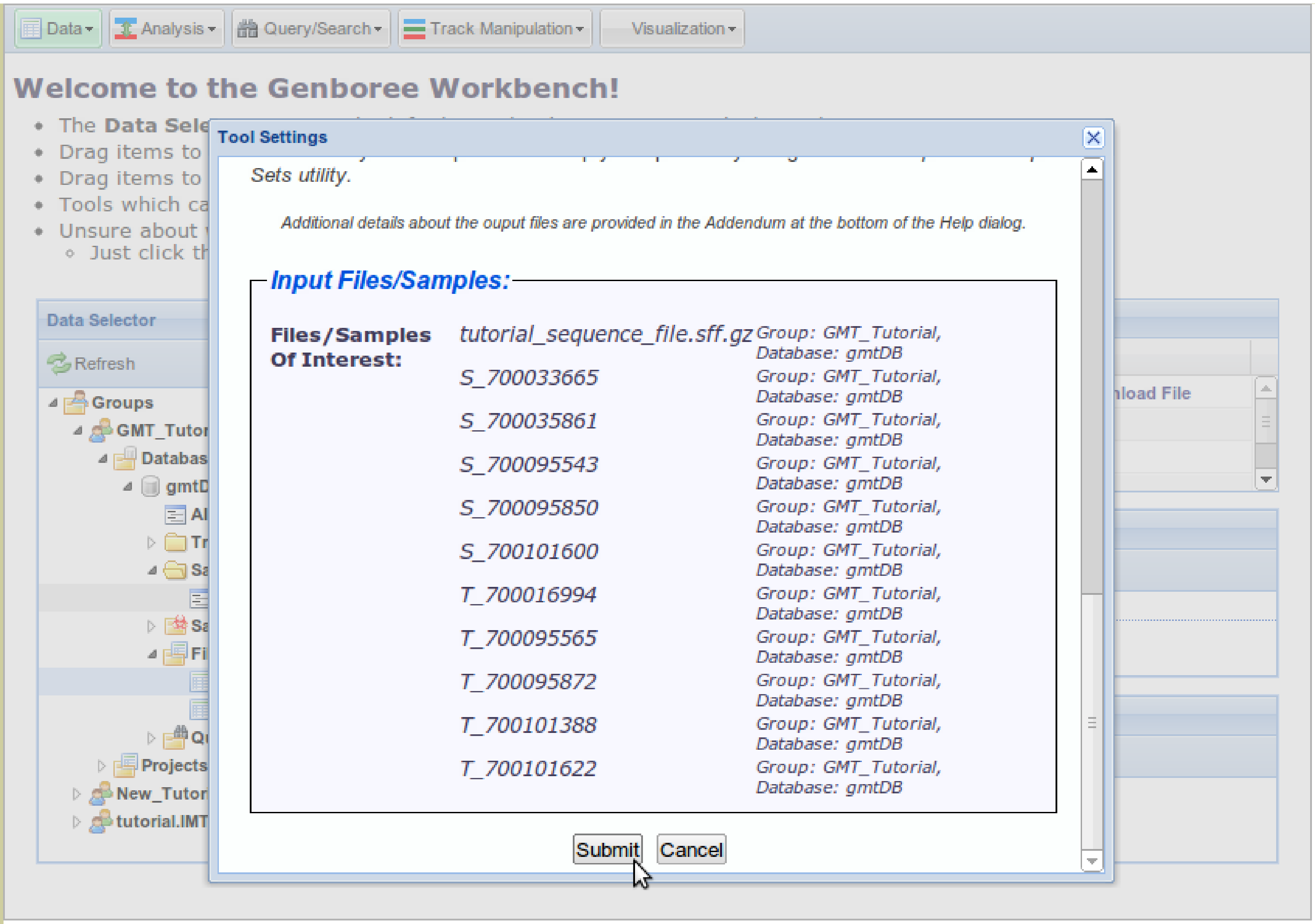


```
Hello Tutorial IMT,

Your Sample - File Linker job has completed successfully.

JOB SUMMARY:
 JobID           : wbJob-samplefilelinker-1312574026_369118

The following file(s) and samples(s) has been linked:
 tutorial_sequence_file.sff.gz(File) -> S_700033665(Sample)
 tutorial_sequence_file.sff.gz(File) -> S_700035861(Sample)
 tutorial_sequence_file.sff.gz(File) -> S_700095543(Sample)
 tutorial_sequence_file.sff.gz(File) -> S_700095850(Sample)
 tutorial_sequence_file.sff.gz(File) -> S_700101600(Sample)
 tutorial_sequence_file.sff.gz(File) -> T_700016994(Sample)
 tutorial_sequence_file.sff.gz(File) -> T_700095565(Sample)
 tutorial_sequence_file.sff.gz(File) -> T_700095872(Sample)
 tutorial_sequence_file.sff.gz(File) -> T_700101388(Sample)
 tutorial_sequence_file.sff.gz(File) -> T_700101622(Sample)

The Genboree Team
```

Import Sequences

h4.

- Drag over the SamplesSet `tutorial_sample_set` from the *Data Selector* window into the the *Input Data* window
  - Note: You can drag over multiple samples, SampleSets, or Sample folders (that have been properly linked) into the *Input Data* window. This allows users to combine interesting data sets without having to import samples, link samples with files, etc. multiple times.
- Drag over the `gmtDB` database from the *Data Selector* window to the *Output Targets* window

- **After you have your samples in the *Input Data* window and your database in the *Output Targets* window, proceed forward**
- **Click the *Analysis* tab, followed by the *Microbiome Workbench* tab, followed by the *Microbiome Sequence Import* tab**
- **Select your options for sequence import**
  - **At this time you can sub-select a set of sequences that you wish to import in the 'Select Samples' window. The default action is to select all samples**
  - **Set a custom 'Sample Set Name' or leave the default (which includes a time stamp)**

- Optionall choose to Trim At Distal Primer, Trim at N/n, Remove sequences which contain an N, set the minimum read length, set the minimum average quality, and set the minimum sequence count
- Click 'Submit'
- Wait for confirmation email

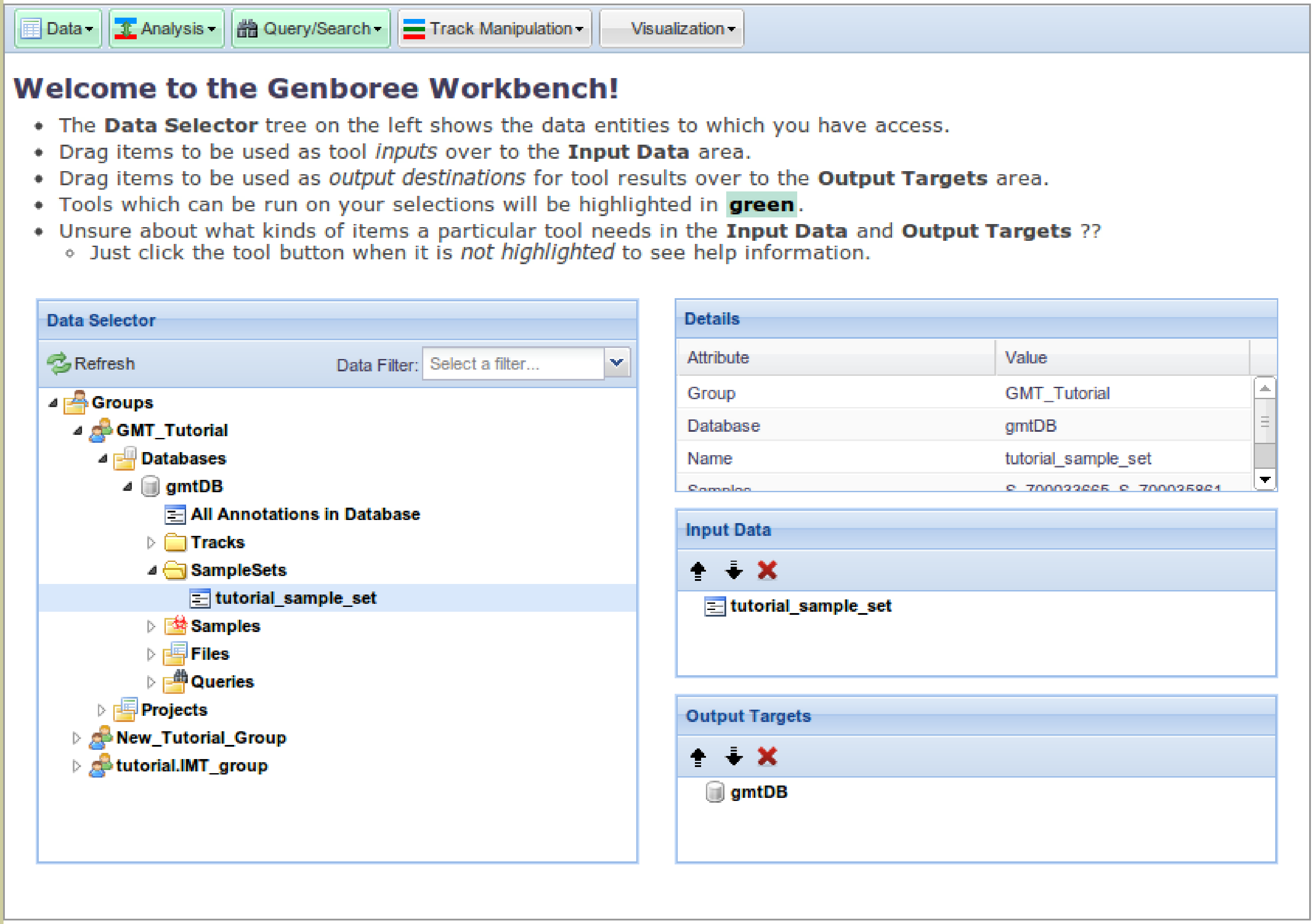

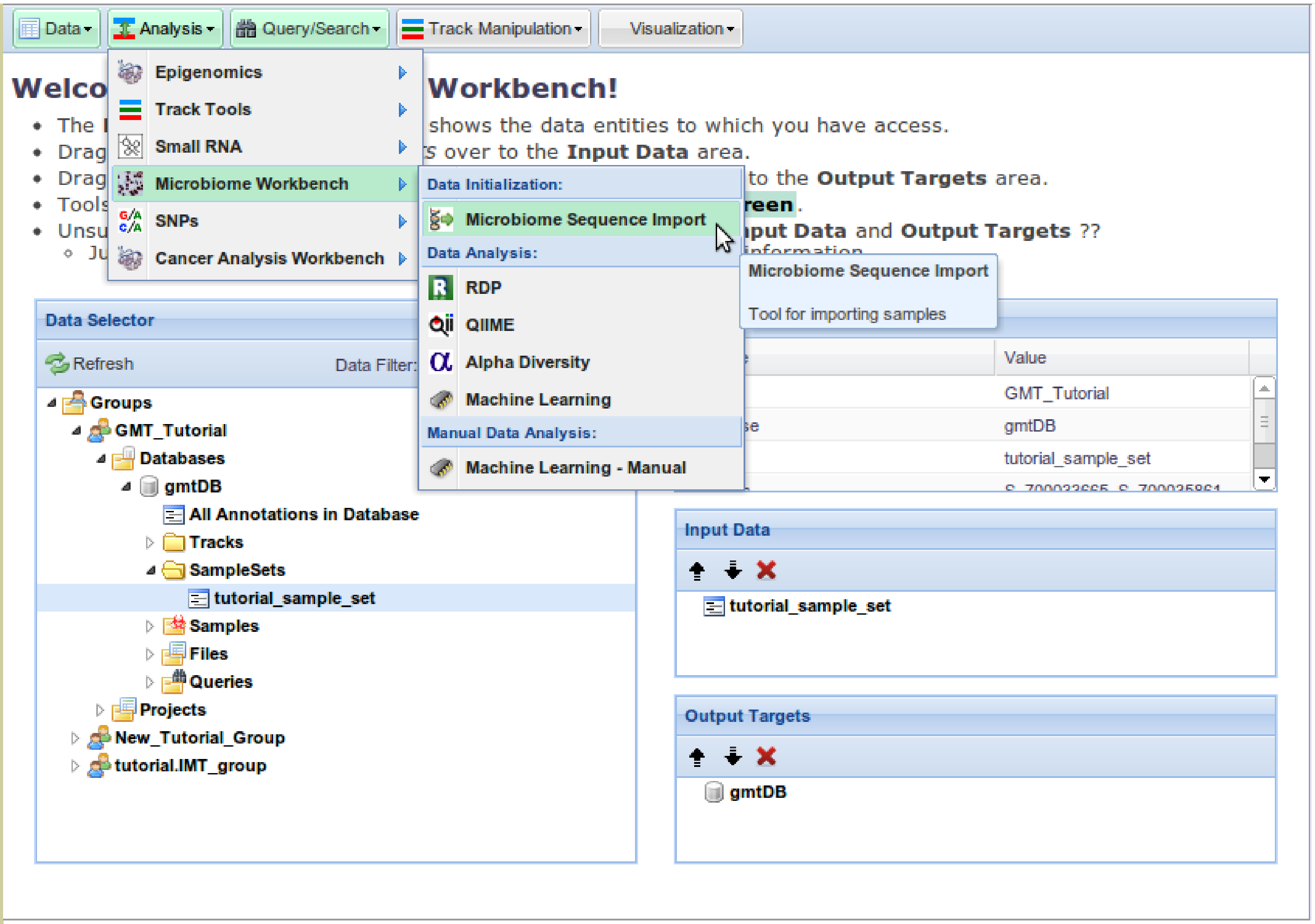

Data
Analysis
Query/Search
Track Manipulation
Visualization
Welco
Workbench!
The
shows the data entities to which you have access.
Drag
over to the Input Data area.
Drag
to the Output Targets area.
Tools
reen.
Unsu
put Data and Output Targets ??
Epigenomics
Track Tools
Small RNA
Microbiome Workbench
SNPs
Cancer Analysis Workbench
Data Initialization:
Microbiome Sequence Import
Data Analysis:
RDP
QIIME
Alpha Diversity
Machine Learning
Manual Data Analysis:
Machine Learning - Manual
Microbiome Sequence Import
Tool for importing samples
Data Selector
Refresh
Data Filter:
Groups
GMT_Tutorial
Databases
gmtDB
All Annotations in Database
Tracks
SampleSets
tutorial_sample_set
Samples
Files
Queries
Projects
New_Tutorial_Group
tutorial.IMT_group
Value
GMT_Tutorial
gmtDB
tutorial_sample_set
Input Data
tutorial_sample_set
Output Targets
gmtDB

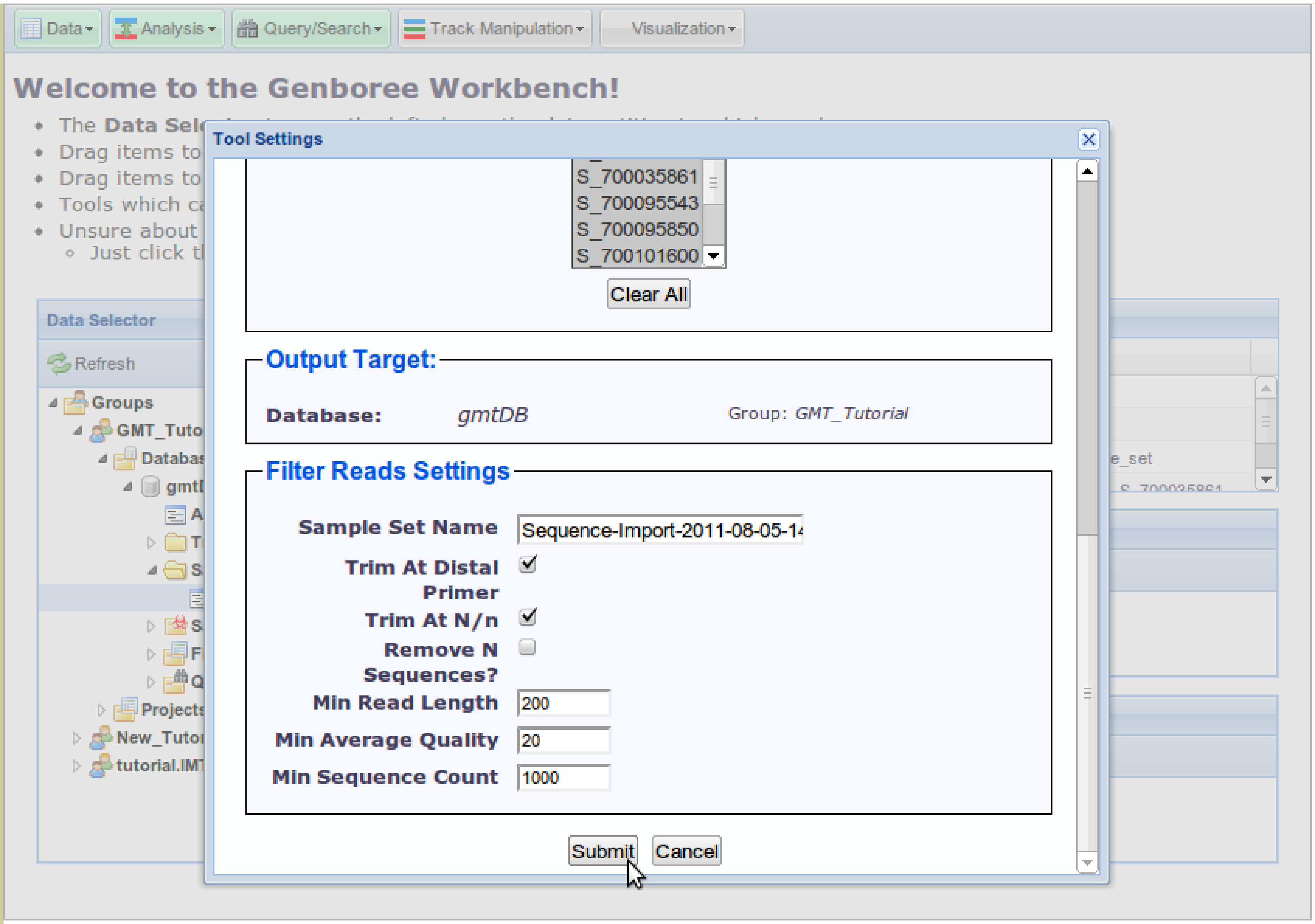


```
Hello Tutorial Imt

Your Microbiome Sequence Import job is complete successfully.

Job Summary:
  JobID                  : wbJob-seqimport-1312574238_616904
  Analysis Name          : Sequence-Import-2011-08-05-14:56:46

Settings:
  minAvgQuality           : 20
  minSeqCount             : 1000
  minSeqLength            : 200
  blastDistalPrimer       : true
  cutAtEnd                : true
  trimLowQualityRun       : false
  removeNSequences        : false

Result File Location in the Genboree Workbench:
  Group : GMT_Tutorial
  DataBase : gmtDB
  Path to File:
     Files
     * MicrobiomeData
        * Sequence-Import-2011-08-05-14:56:46

The Genboree Team
```

View Imported Sequences

- Click the *Refresh* button in the *Data Selector* window
- Expand `Groups -> GMT_Tutorial -> Databases -> gmtDB -> Files -> MicrobiomeData -> Sequence-Import-2011-08-05-14:56:46` to see that your sequences have been imported
  - fastq
    - fastq files for each uploaded SFF/SRA file
    - fastq is a file format that represents the combination of the fasta and quality score files
  - sample.metadata
    - Sample meta data file representing all samples used for analysis (appended with sequence import parameters, flags,

etc. that are used for the pipeline)
  - settings.json
    - Settings in json format for sequence import pipeline
  - fasta.result.tar.gz
    - fasta file for each uploaded SFF/SRA file
  - filtered_fasta.result.tar.gz
    - Final quality filtered fasta file for each sample
  - stats.result.tar.gz
    - Sequence metrics for each sample
  - jobFile.json
    - See settings.json
  - sequences_metrics_summary.xls
    - Sequence metrics broken down into individual samples, summary for all samples, and each meta data label.

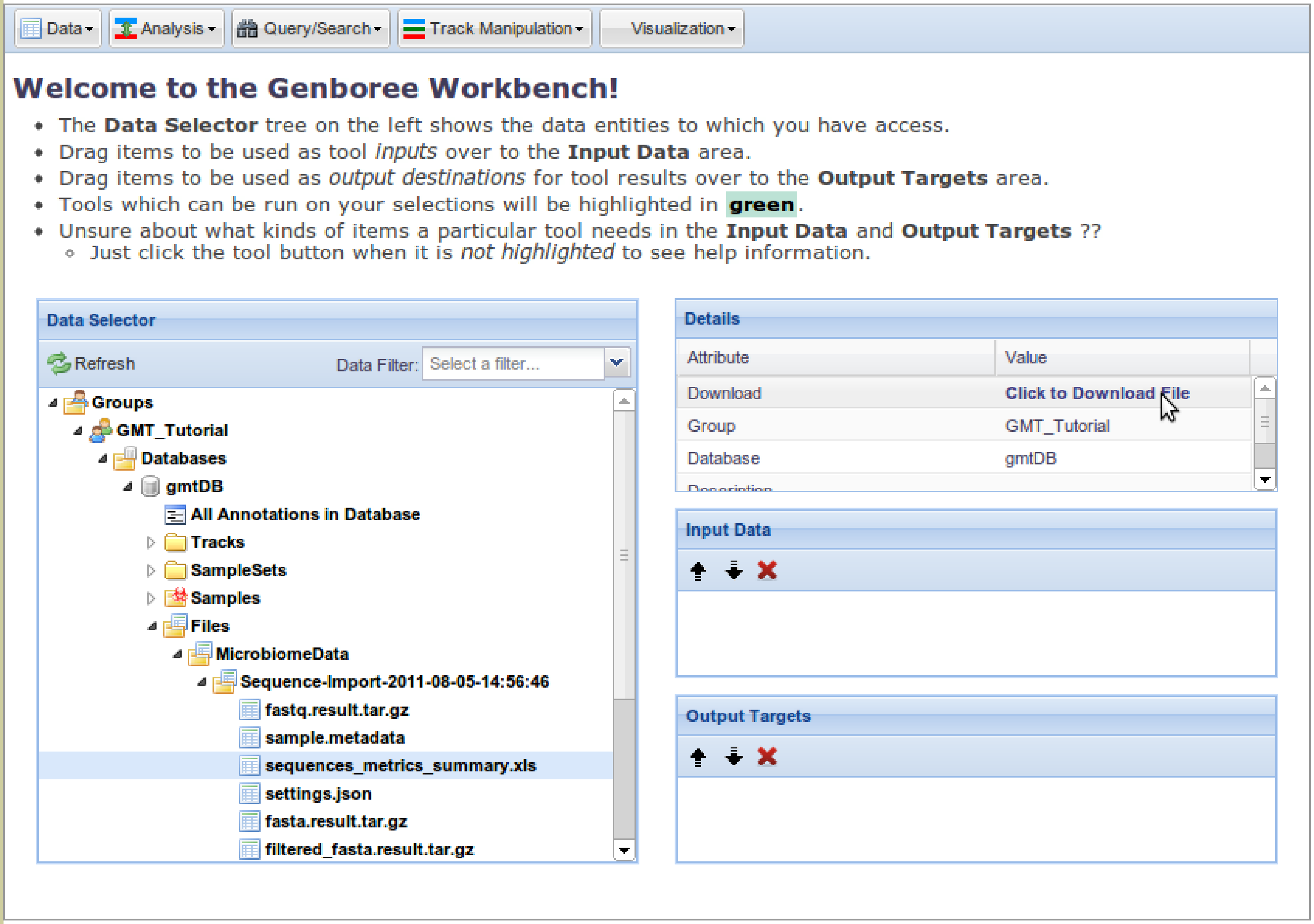

sampleName	Average_read_length	total_sequence_counts_after_filter	body_site
S_700033665	505	7008	Stool
S_700101600	506	6716	Stool
T_700101622	515	4658	Throat
T_700016994	512	6794	Throat
S_700035861	511	6819	Stool
S_700095850	500	5879	Stool
T_700095872	516	2543	Throat
S_700095543	503	6191	Stool
T_700101388	510	7527	Throat
T_700095565	516	6294	Throat

	Average Sequence Length	Total Sequences

508	60429

RDP - Taxonomic Abundance Pipeline

- Drag `Sequence-Import-2011-08-05-14:56:46` from the *Data Selector* window to the *Input Data* window
- Drag over the `gmtDB` into the *Output Targets* window
- Drag over the `gmtProject` into the *Output Targets* window
  - This project is visible if you expand `Groups` -> `GMT_Tutorial` -> `Projects` -> `gmtProject`
- Click the *Analysis* tab, followed by the *Microbiome Workbench* tab, followed by the *RDP* tab
- You can optionally fill in a 'Study Name' to organize your individual runs. We will use 'Tutorial_Study' here.
- Click 'Submit'
- Wait for confirmation email

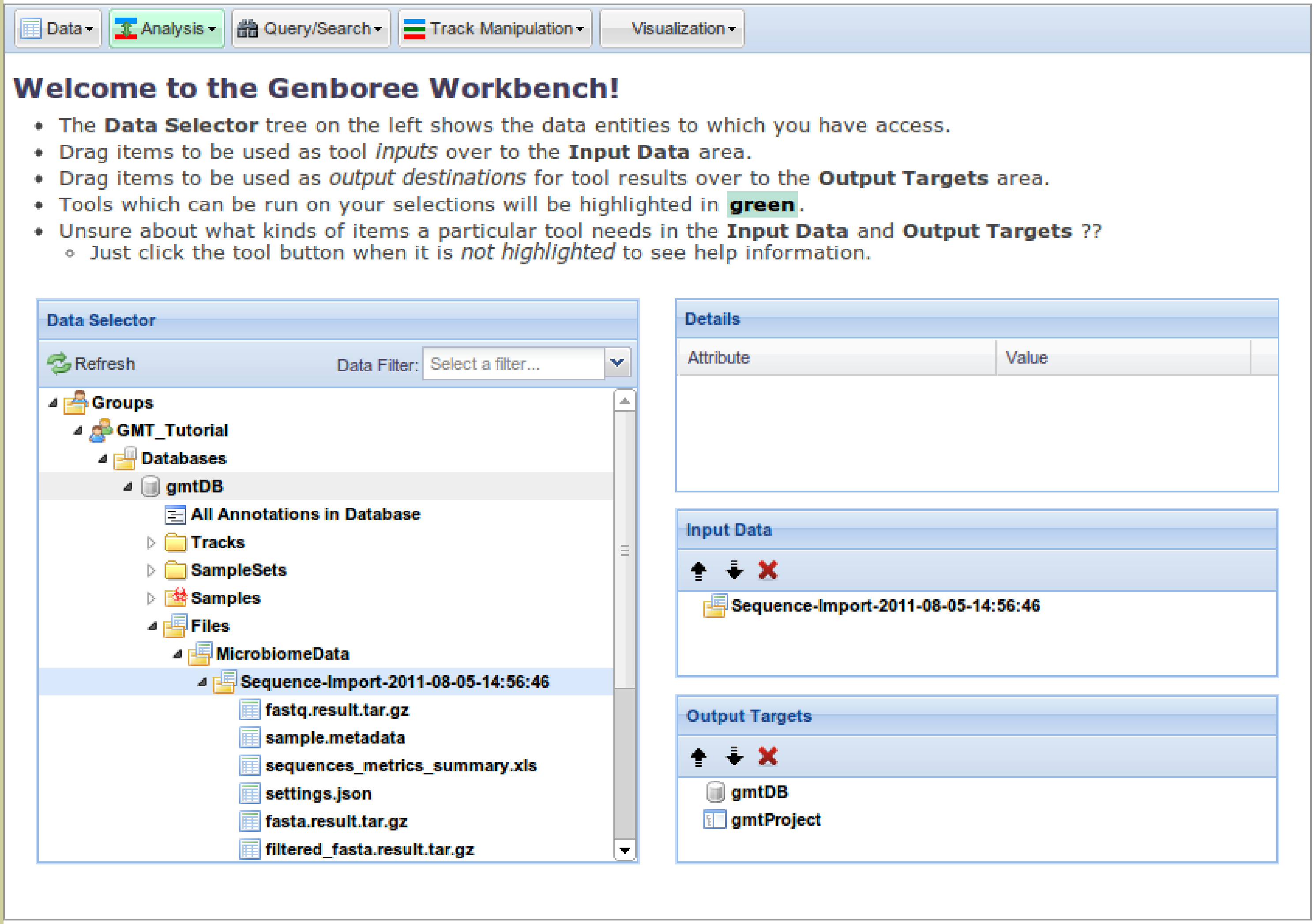

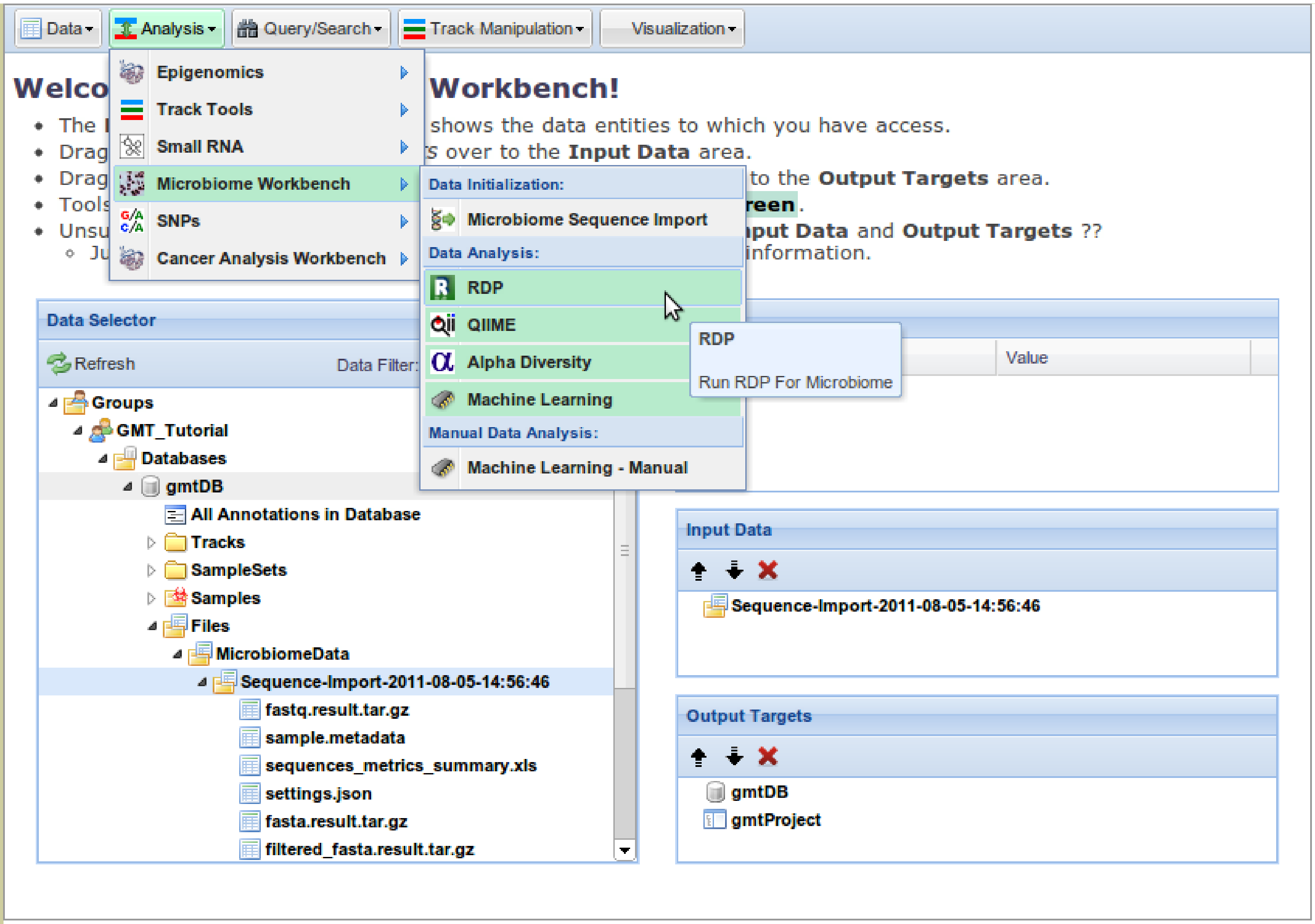

Data
Analysis
Query/Search
Track Manipulation
Visualization
Welco
Workbench!
The
shows the data entities to which you have access.
Drag
s over to the Input Data area.
Drag
to the Output Targets area.
Tools
reen.
Unsu
put Data and Output Targets ??
Ju
information.
Epigenomics
Track Tools
Small RNA
Microbiome Workbench
SNPs
Cancer Analysis Workbench
Data Initialization:
Microbiome Sequence Import
Data Analysis:
RDP
QIIME
Alpha Diversity
Machine Learning
Manual Data Analysis:
Machine Learning - Manual
RDP
Run RDP For Microbiome
Value
Data Selector
Refresh
Data Filter:
Groups
GMT_Tutorial
Databases
gmtDB
All Annotations in Database
Tracks
SampleSets
Samples
Files
MicrobiomeData
Sequence-Import-2011-08-05-14:56:46
fastq.result.tar.gz
sample.metadata
sequences_metrics_summary.xls
settings.json
fasta.result.tar.gz
filtered_fasta.result.tar.gz
Input Data
Sequence-Import-2011-08-05-14:56:46
Output Targets
gmtDB
gmtProject

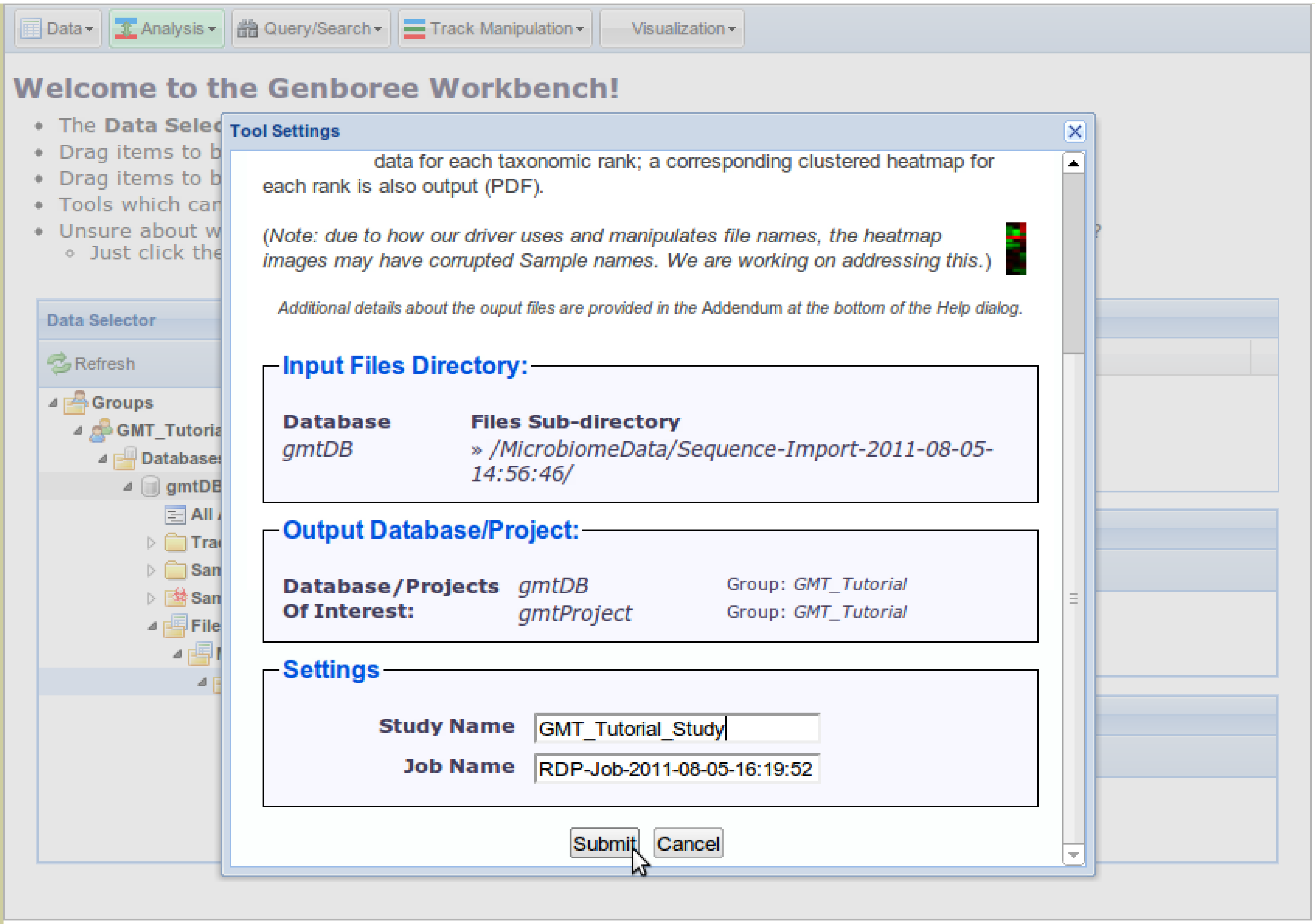


```
  Hello Tutorial Imt

   Your RDP job is complete successfully.

   Job Summary:

   JobID                    : wbJob-rdp-1312579221_728029
   Study Name               : GMT_Tutorial_Study
   Job Name                 : RDP-Job-2011-08-05-16:19:52

   Settings:
   rdpVersion: 2.2
   rdpBootstrapCutoff: 0.8

   Result File Location in the Genboree Workbench:
   Group : GMT_Tutorial
   DataBase : gmtDB
   Path to File:
   Files
   * MicrobiomeWorkBench
     * GMT_Tutorial_Study
       *RDP
         *RDP-Job-2011-08-05-16:19:52

Plots URL (click or paste in browser to access file):
   Prj: gmtProject
   URL:
http://genboree.org/java-bin/project.jsp?projectName=gmtProject

   The Genboree Team
```

RDP Results

- Click the *Refresh* button in the *Data Selector* window
- Expand `Groups` -> `GMT_Tutorial` -> `Databases` -> `gmtDB` -> `Files` -> `MicrobiomeWorkBench` -> `Tutorial_Study` -> `RDP` -> `RDP-Job-2011-08-05-16:19:52`
- Domain/Phyla/Class/Order/Family/Genus/Species.result.tar.gz
  - Individual samples separated into results based on separate taxonomic depth

- counts.xlsx
  - Raw counts of the appearance of each taxonomic depth (per sample) weighted by the RDP bootstrap classification score (i.e. 85% counts for 0.85 of an occurrence, 100% counts for 1.00 of an occurrence, etc.)
- normalized.xlsx
  - Normalized counts of the appearance of each taxonomic depth (per sample) that sums to approximately 1.00.
- Heatmaps of each taxonomic depth are accessible via the `Tutorial_Study` project page
  - http://www.genboree.org/java-bin/project.jsp?projectName=gmtProject

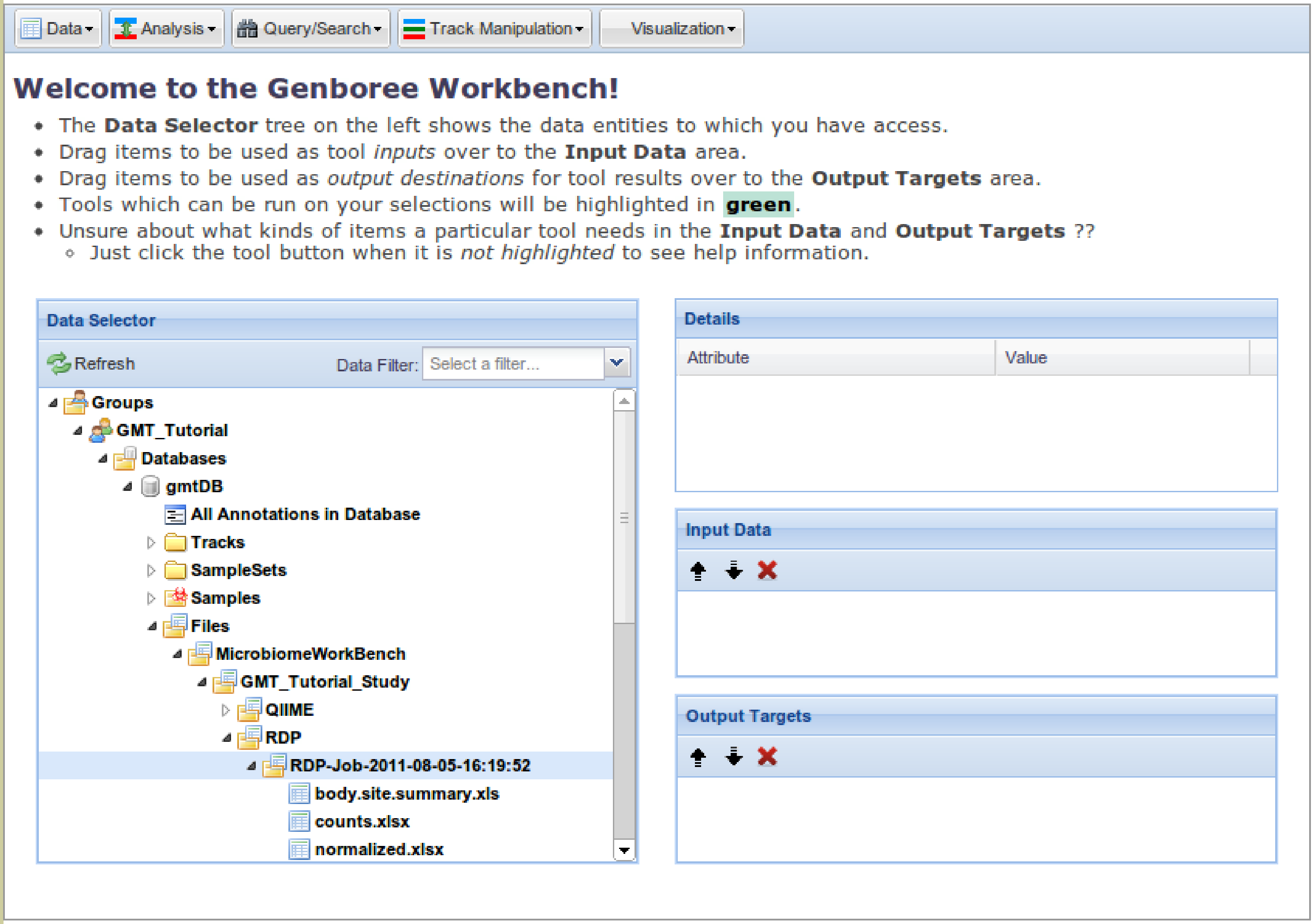

(This is a recently added feature. Report issues to **Genboree Admin**.)

Edit Mode

gmtProject

[[Put description for the project 'gmtProject' here]]

Project News:

2011/8/8: Tutorial lmt ran a Alpha Diversity job (AD-Job-2011-08-08-09:09:58) and the results are available at the link below.

- **Study Name**: GMT_Tutorial_Study
- **Job Name**: AD-Job-2011-08-08-09:09:58
- **Link to result plots**

2011/8/8: Tutorial lmt ran a Machine Learning job (ML-Job-2011-08-08-09:13:04) and the results are available at the link below.

- **Study Name**: GMT_Tutorial_Study
- **Job Name**: ML-Job-2011-08-08-09:13:04
- **Link to result plots**

2011/8/5: Tutorial lmt ran a QIIME job (Qiime-Job-2011-08-05-16:22:08) and the results are available at the links below.

- **Study Name**: GMT_Tutorial_Study
- **Job Name**: Qiime-Job-2011-08-05-16:22:08
- **Link to cdhit results**
- **Link to cdhit-normalized results**
- **Link to phylogenetic tree results**

2011/8/5: Tutorial lmt ran a RDP job (RDP-Job-2011-08-05-16:19:52) and the results are available at the link below.

- **Study Name**: GMT_Tutorial_Study
- **Job Name**: RDP-Job-2011-08-05-16:19:52
- **Link to result plots**

Table of Content: RDP Results

**Study Name:**	GMT_Tutorial_Study
**Job Name:**	RDP-Job-2011-08-05-16:19:52
**User:**	Tutorial lmt
**Date:**	2011/08/05 16:37 CDT

RDP Plots

Class-normalized
Order-normalized
Family-normalized
Genus-normalized
Family-normalized.meta.body_site
Genus-normalized.meta.body_site
Order-normalized.meta.body_site
Phylum-normalized.meta.body_site
Domain-normalized.meta.body_site
Class-normalized.meta.body_site

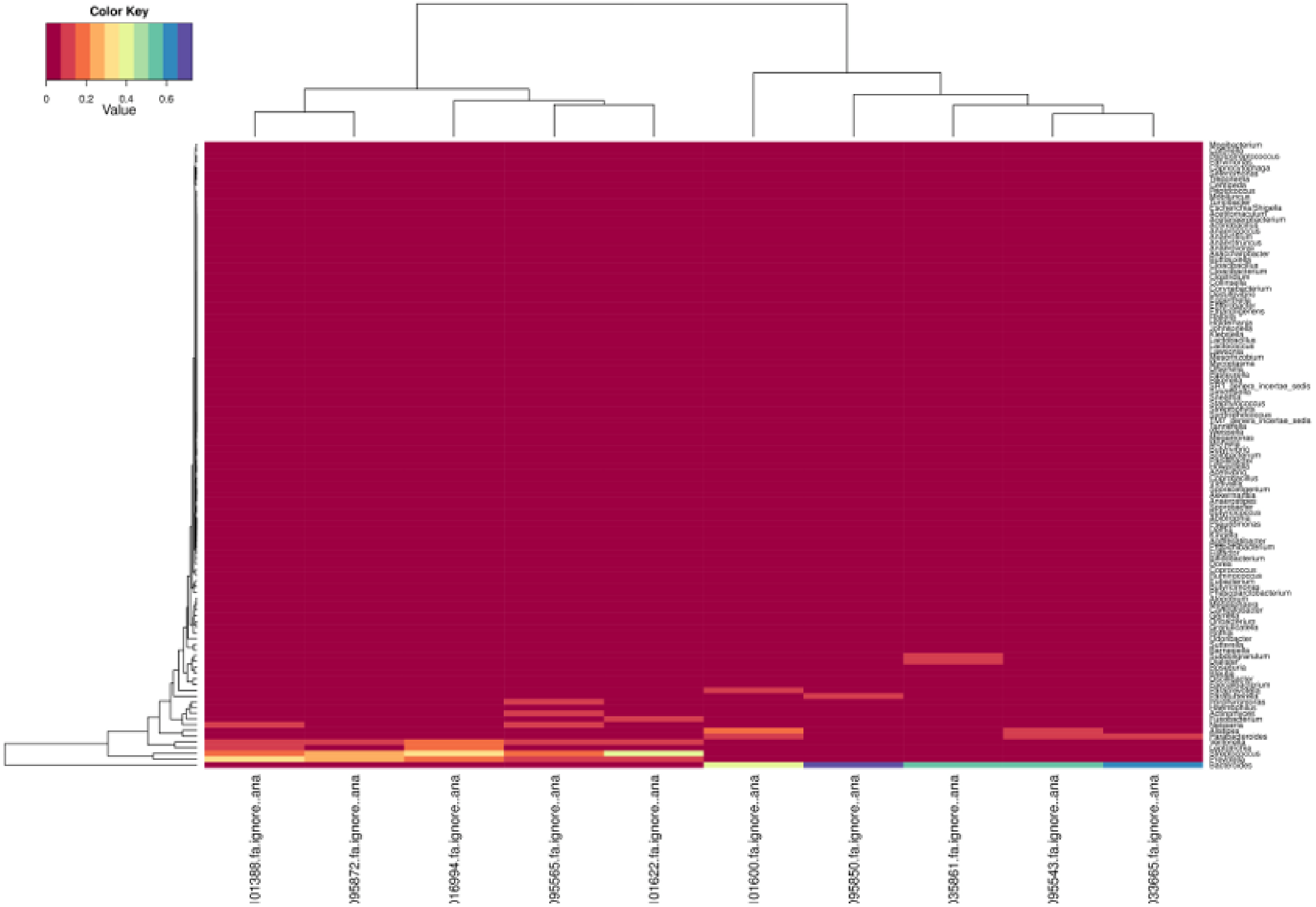

Color Key
0 0.2 0.4 0.6
Value
l01388.fa.ignore..ana
)95872.fa.ignore..ana
)16994.fa.ignore..ana
)95565.fa.ignore..ana
l01622.fa.ignore..ana
l01600.fa.ignore..ana
)95850.fa.ignore..ana
)35861.fa.ignore..ana
)95543.fa.ignore..ana
)33665.fa.ignore..ana

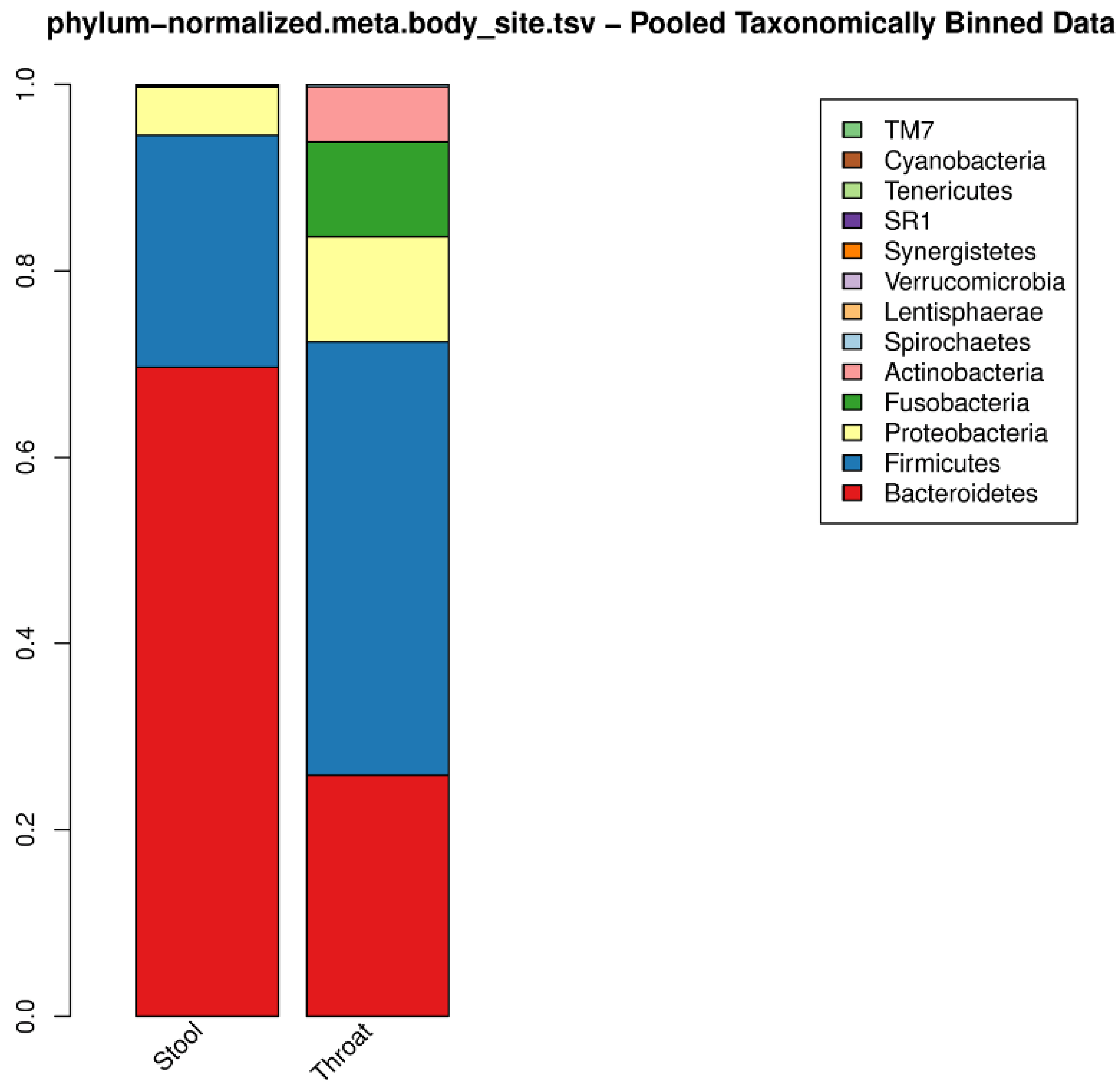

QIIME Pipeline - OTU Table, Phylogenetic Tree, and Beta Diversity

- Drag `Sequence-Import-2011-08-05-14:56:46` from the *Data Selector* window to the *Input Data* window
- Drag over the `gmtDB` into the *Output Targets* window
- Drag over the `gmtProject` into the *Output Targets* window
  - This project is visible if you expand `Groups` -> `GMT_Tutorial` -> `Projects` -> `gmtProject`
- Click the *Analysis* tab, followed by the *Microbiome Workbench* tab, followed by the *QIIME* tab
- You can optionally fill in a 'Study Name' to organize your individual runs. We will use 'Tutorial_Study' here.
- You can optionally choose to remove chimeras with Chimera Slayer
- Click 'Submit'
- Wait for confirmation email

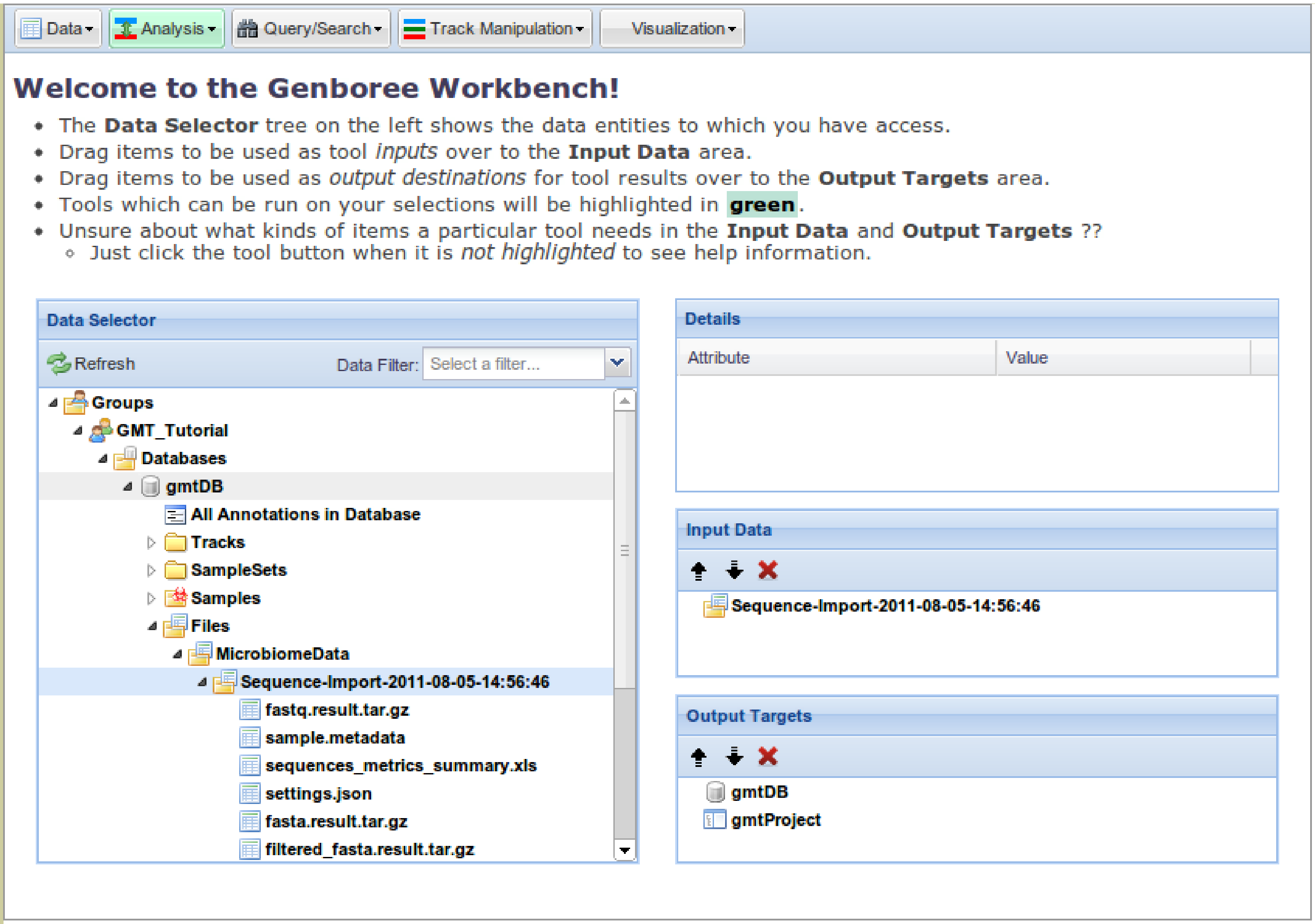
Data
Analysis
Query/Search
Track Manipulation
Visualization
Welcome to the Genboree Workbench!
The Data Selector tree on the left shows the data entities to which you have access.
Drag items to be used as tool inputs over to the Input Data area.
Drag items to be used as output destinations for tool results over to the Output Targets area.
Tools which can be run on your selections will be highlighted in green.
Unsure about what kinds of items a particular tool needs in the Input Data and Output Targets ??
Just click the tool button when it is not highlighted to see help information.
Data Selector
Refresh
Data Filter:
Select a filter...
Groups
GMT_Tutorial
Databases
gmtDB
All Annotations in Database
Tracks
SampleSets
Samples
Files
MicrobiomeData
Sequence-Import-2011-08-05-14:56:46
fastq.result.tar.gz
sample.metadata
sequences_metrics_summary.xls
settings.json
fasta.result.tar.gz
filtered_fasta.result.tar.gz
Details
Attribute
Value
Input Data
Sequence-Import-2011-08-05-14:56:46
Output Targets
gmtDB
gmtProject

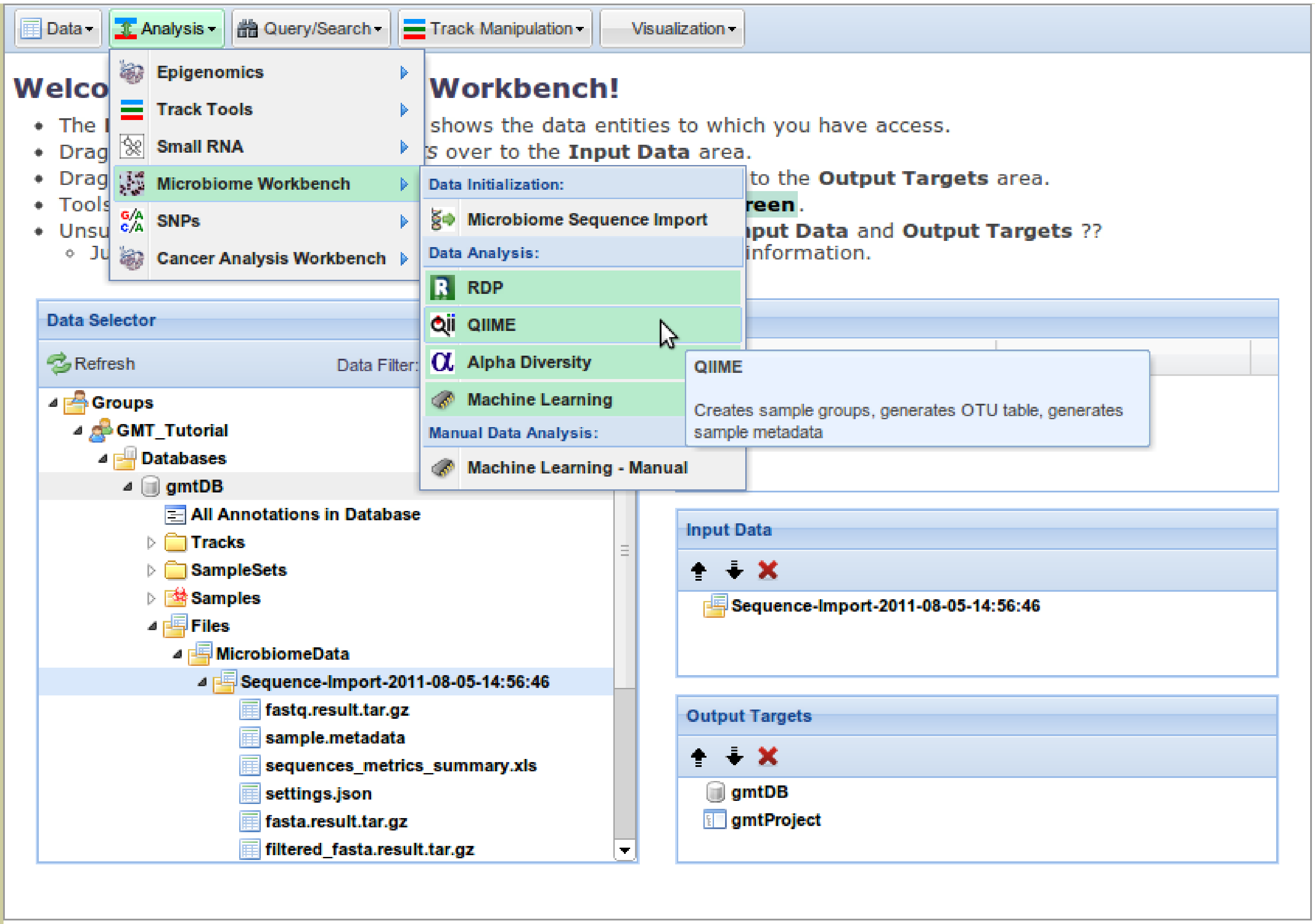
Data
Analysis
Query/Search
Track Manipulation
Visualization
Workbench!
shows the data entities to which you have access.
s over to the Input Data area.
to the Output Targets area.
reen.
put Data and Output Targets ??
information.
Epigenomics
Track Tools
Small RNA
Microbiome Workbench
SNPs
Cancer Analysis Workbench
Data Initialization:
Microbiome Sequence Import
Data Analysis:
RDP
QIIME
Alpha Diversity
Machine Learning
Manual Data Analysis:
Machine Learning - Manual
QIIME
Creates sample groups, generates OTU table, generates sample metadata
Data Selector
Refresh
Data Filter:
Groups
GMT_Tutorial
Databases
gmtDB
All Annotations in Database
Tracks
SampleSets
Samples
Files
MicrobiomeData
Sequence-Import-2011-08-05-14:56:46
fastq.result.tar.gz
sample.metadata
sequences_metrics_summary.xls
settings.json
fasta.result.tar.gz
filtered_fasta.result.tar.gz
Input Data
Sequence-Import-2011-08-05-14:56:46
Output Targets
gmtDB
gmtProject

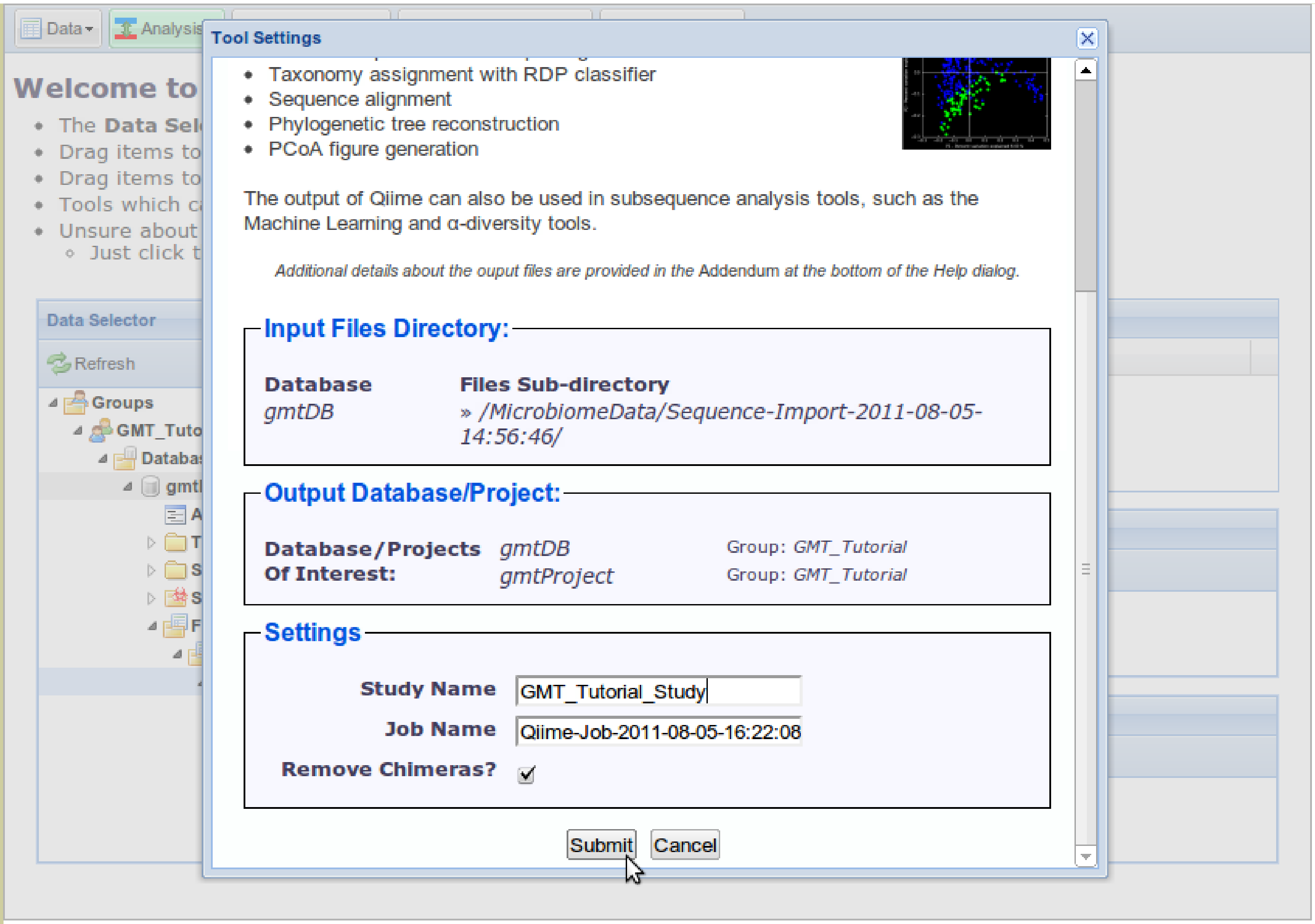


```
Hello Tutorial Imt

Your QIIME job is completed successfully.

Job Summary:
  JobID                    : wbJob-qiime-1312579361_942462
  Study Name               : GMT_Tutorial_Study
  Job Name                 : Qiime-Job-2011-08-05-16:22:08

Result File Location in the Genboree Workbench:
  Group : GMT_Tutorial
  DataBase : gmtDB
  Path to File:
     Files
     * MicrobiomeWorkBench
        * GMT_Tutorial_Study
           *QIIME
              *Qiime-Job-2011-08-05-16:22:08

Plots URL (click or paste in browser to access file):
   Prj: gmtProject
   URL:
http://genboree.org/java-bin/project.jsp?projectName=gmtProject

The Genboree Team
```

QIIME Results

- **Click the *Refresh* button in the *Data Selector* window**
- **Expand `Groups` -> `GMT_Tutorial` -> `Databases` -> `gmtDB` -> `Files` -> `MicrobiomeWorkBench` -> `Tutorial_Study` -> `QIIME` -> `Qiime-Job-2011-08-05-16:22:08`**
  - **mapping.txt**
    - **QIIME sample meta data mapping file**
  - **raw.results.tar.gz**
    - **Full compressed results from the pipeline**
  - **sample.metadata**
  - **settings.json**

- plots.result.tar.gz
    - 2D and 3D plots
- fasta.result.tar.gz
    - Representative sequences aligned files
- taxonomy.result.tar.gz
    - OTU tables separated by taxonomic depth
- otu.table
- phylogenetic.result.tar.gz
    - Representative sequence files: aligned, datafile, tree file, itol tree file, and tree file parsed
- jobFile.json

- 2D and 3D plots can be viewed at the project page
    - http://www.genboree.org/java-bin/project.jsp?projectName=gmtProject

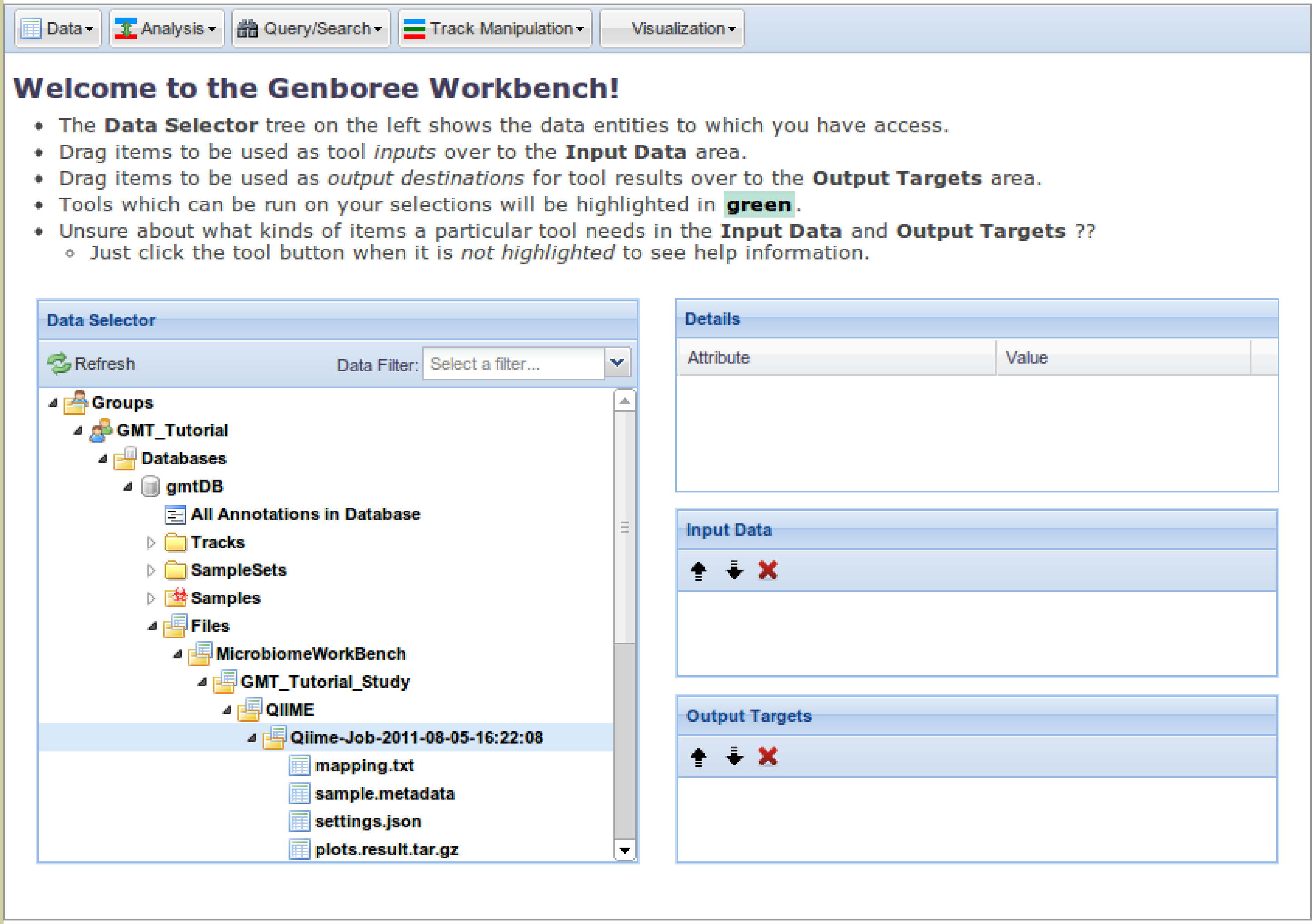

(This is a recently added feature. Report issues to **Genboree Admin**.)

Edit Mode

gmtProject

[[Put description for the project 'gmtProject' here]]

Project News:

2011/8/8: Tutorial Imt ran a Alpha Diversity job (AD-Job-2011-08-08-09:09:58) and the results are available at the link below.

- **Study Name**: GMT_Tutorial_Study
- **Job Name**: AD-Job-2011-08-08-09:09:58
- **Link to result plots**

2011/8/8: Tutorial Imt ran a Machine Learning job (ML-Job-2011-08-08-09:13:04) and the results are available at the link below.

- **Study Name**: GMT_Tutorial_Study
- **Job Name**: ML-Job-2011-08-08-09:13:04
- **Link to result plots**

2011/8/5: Tutorial Imt ran a QIIME job (Qiime-Job-2011-08-05-16:22:08) and the results are available at the links below.

- **Study Name**: GMT_Tutorial_Study
- **Job Name**: Qiime-Job-2011-08-05-16:22:08
- **Link to cdhit results**
- **Link to cdhit-normalized results**
- **Link to phylogenetic tree results**

2011/8/5: Tutorial Imt ran a RDP job (RDP-Job-2011-08-05-16:19:52) and the results are available at the link below.

- **Study Name**: GMT_Tutorial_Study
- **Job Name**: RDP-Job-2011-08-05-16:19:52
- **Link to result plots**

Table of Content: QIIME Results (cdhit-normalized)

Study Name: GMT_Tutorial_Study
Job Name: Qiime-Job-2011-08-05-16:22:08
User: Tutorial Imt
Date: 2011/08/05 17:16 CDT

Below are the beta diversity metric results for this QIIME job. Clicking on the links will open up metric plots/images on your browser. Pages with **2D Plots** will show a simple 2-dimensional image, while pages with **3D Plots** will use a Java Applet to view and manipulate the 3-dimensional plot (Download Java)
.

QIIME Plots

Binary Chord (3D) (2D)
Binary Euclidean (3D) (2D)
Binary Hamming (3D) (2D)
Binary Jaccard (3D) (2D)
Binary Lennon (3D) (2D)
Binary Ochiai (3D) (2D)
Binary Pearson (3D) (2D)
Binary Sorensen Dice (3D) (2D)
Bray Curtis (3D) (2D)
Canberra (3D) (2D)
Chi-squared (3D) (2D)
Chord (3D) (2D)
Euclidean (3D) (2D)
Gower (3D) (2D)

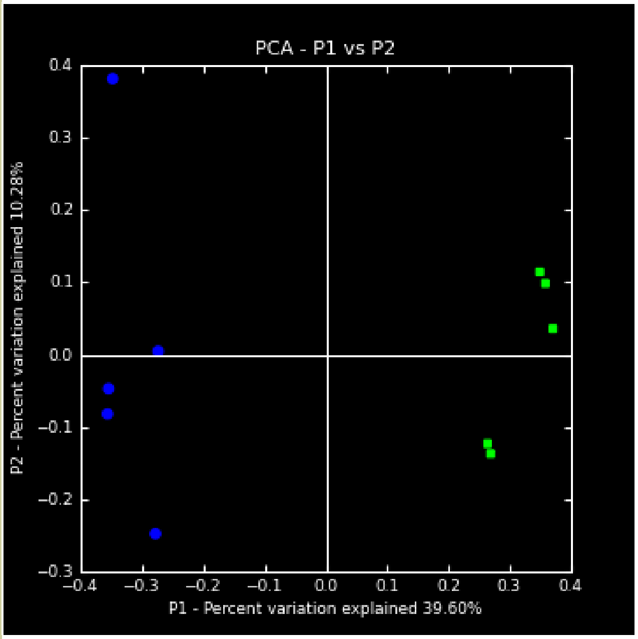

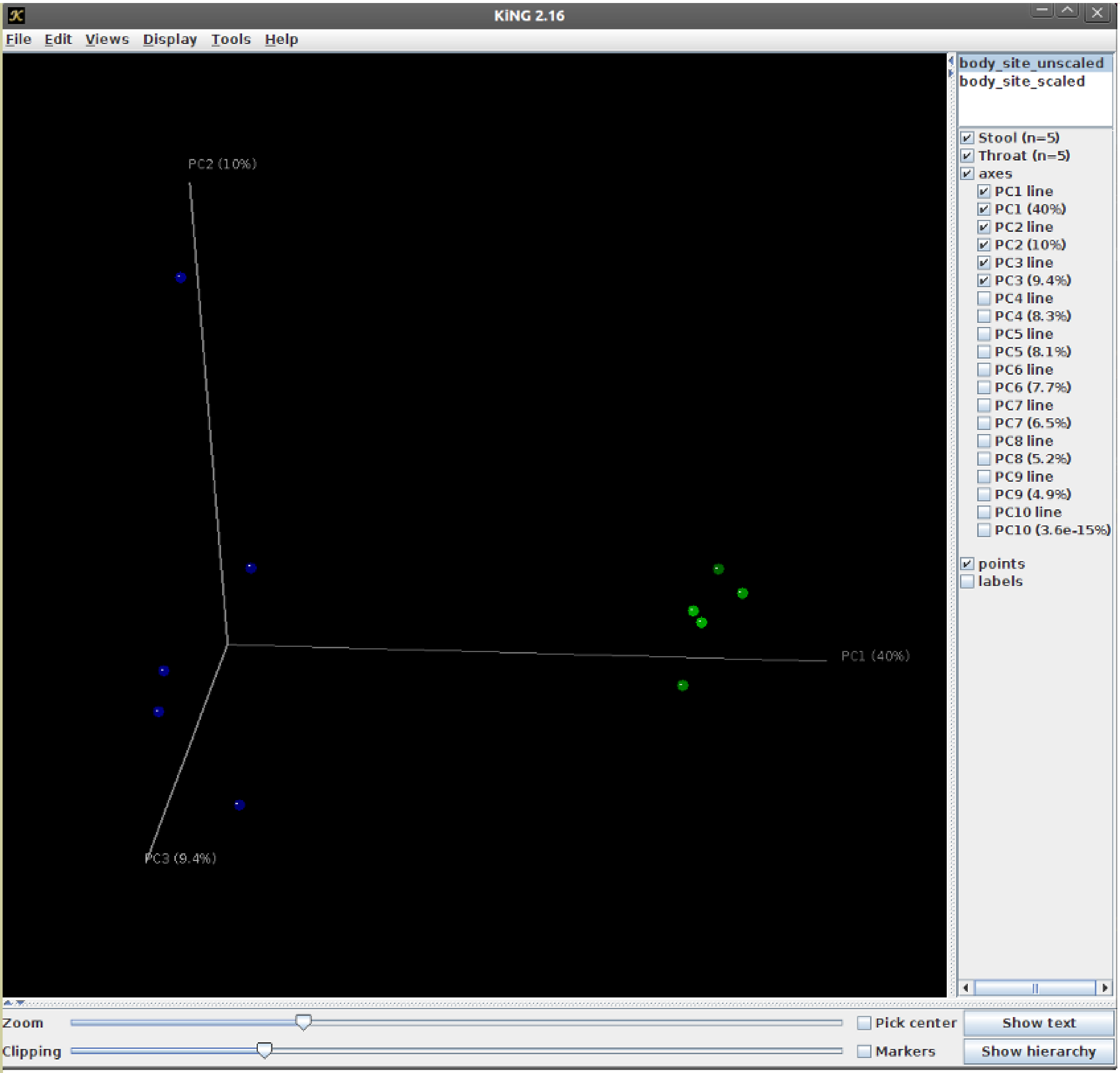

Alpha Diversity

- Drag Qiime-Job-2011-08-05-16:22:08 into the *Input Data* window
  - Accessible via `Groups -> GMT_Tutorial -> Databases -> gmtDB -> Files -> MicrobiomeWorkBench -> Tutorial_Study -> QIIME -> Qiime-Job-2011-08-05-16:22:08`
- Drag over the `gmtDB` into the *Output Targets* window
- Drag over the `gmtProject` into the *Output Targets* window
- Click the *Analysis* tab, followed by the *Microbiome Workbench* tab, followed by the *Alpha Diversity* tab
- Optionally fill in a 'Study Name', here we'll use 'Tutorial_Study'
- Select one or many feature lists, which was accessible via the user provided sample meta data
- Optionally remove singletons
  - Singletons are entries in the OTU tables that only exist once in all samples. These elements can falsely raise diversity and have been known to impact alpha diversity curves.
- Click 'Submit'
- Wait for confirmation email

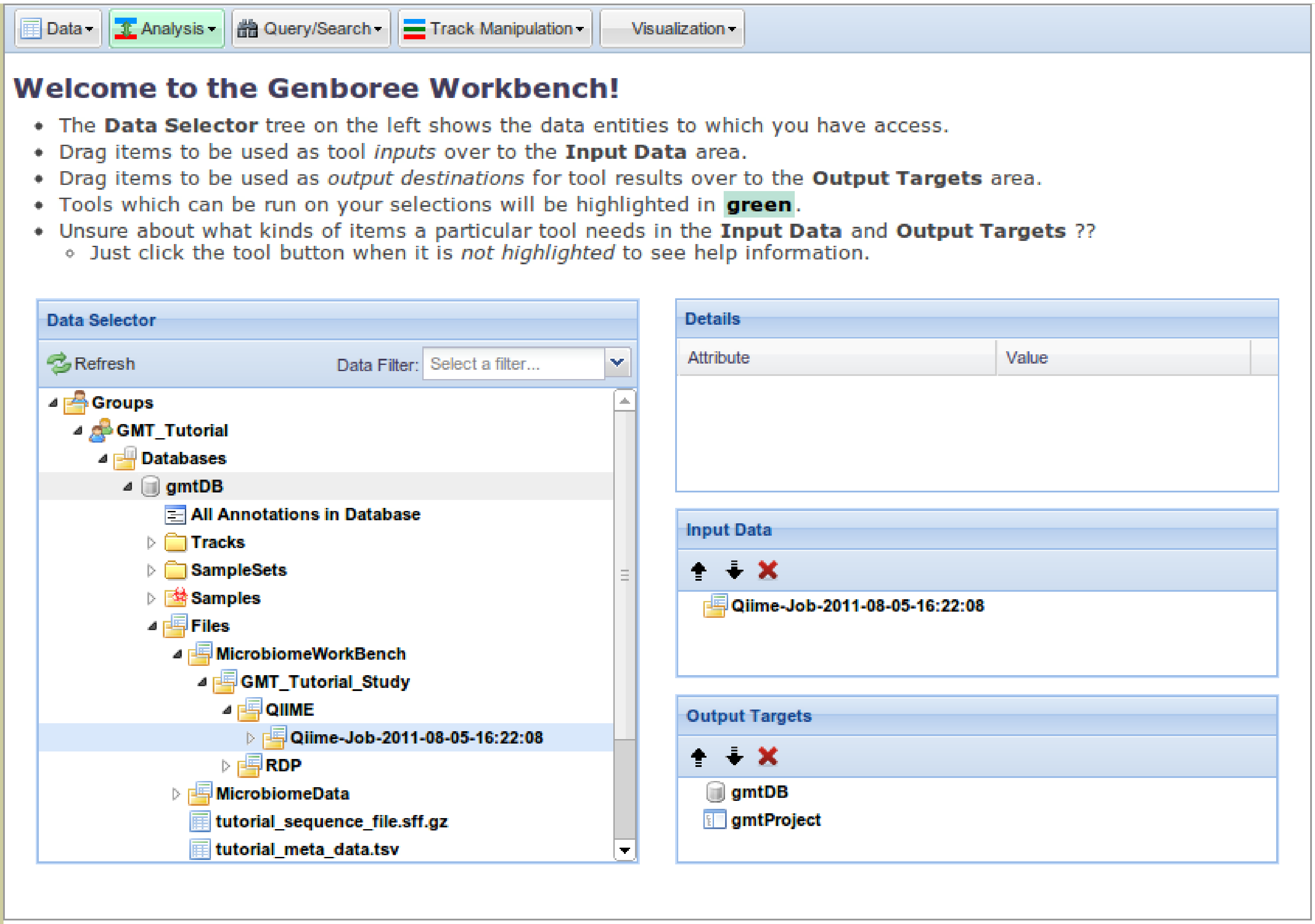

Data
Analysis
Query/Search
Track Manipulation
Visualization
Welcome to the Genboree Workbench!
The Data Selector tree on the left shows the data entities to which you have access.
Drag items to be used as tool inputs over to the Input Data area.
Drag items to be used as output destinations for tool results over to the Output Targets area.
Tools which can be run on your selections will be highlighted in green.
Unsure about what kinds of items a particular tool needs in the Input Data and Output Targets ??
Just click the tool button when it is not highlighted to see help information.
Data Selector
Refresh
Data Filter:
Select a filter...
Groups
GMT_Tutorial
Databases
gmtDB
All Annotations in Database
Tracks
SampleSets
Samples
Files
MicrobiomeWorkBench
GMT_Tutorial_Study
QIIME
Qiime-Job-2011-08-05-16:22:08
RDP
MicrobiomeData
tutorial_sequence_file.sff.gz
tutorial_meta_data.tsv
Details
Attribute
Value
Input Data
Qiime-Job-2011-08-05-16:22:08
Output Targets
gmtDB
gmtProject

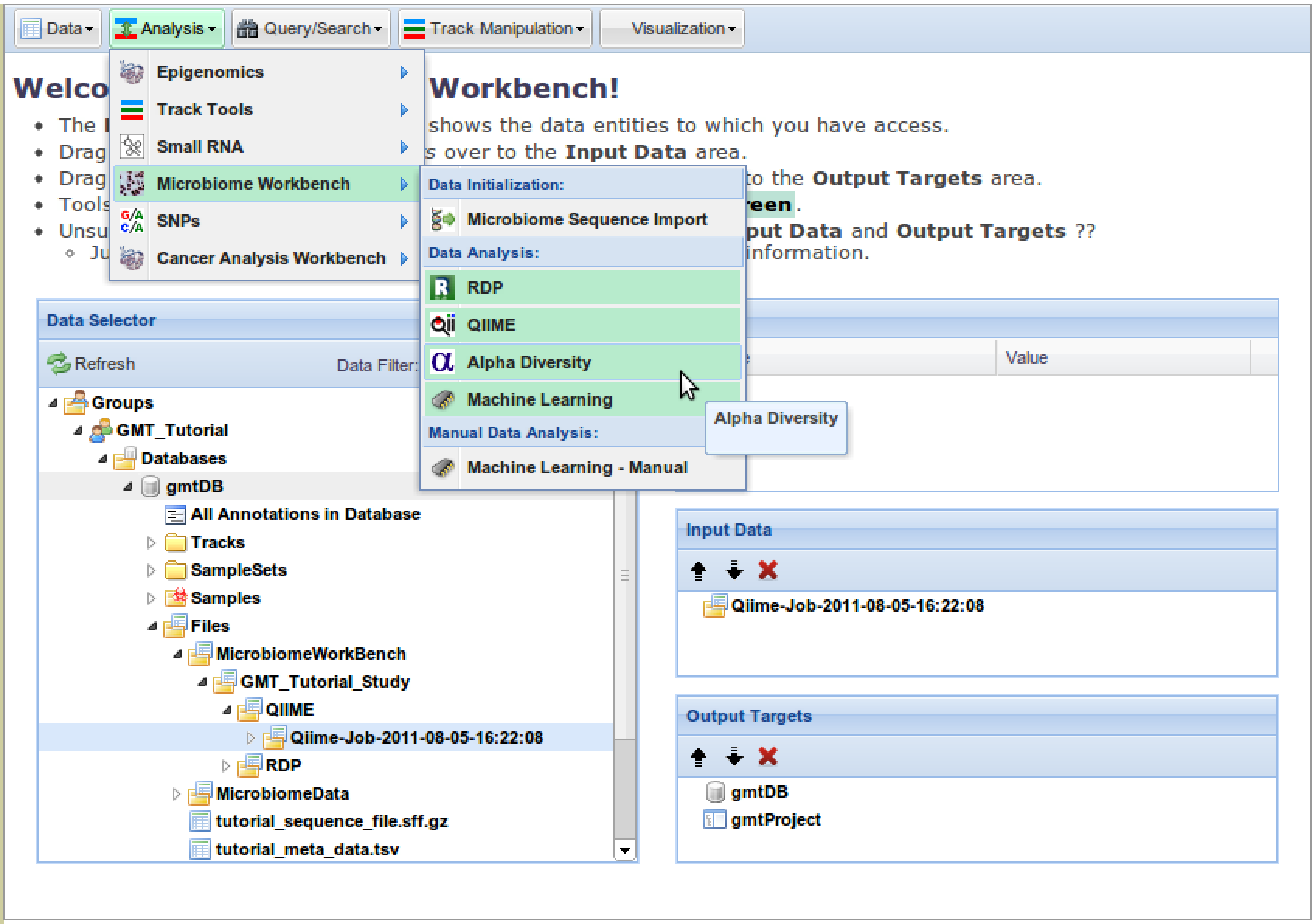
Data
Analysis
Query/Search
Track Manipulation
Visualization
Welco
Workbench!
The
shows the data entities to which you have access.
Drag
s over to the Input Data area.
Drag
to the Output Targets area.
Tools
een.
Unsu
put Data and Output Targets ??
Ju
information.
Epigenomics
Track Tools
Small RNA
Microbiome Workbench
SNPs
Cancer Analysis Workbench
Data Initialization:
Microbiome Sequence Import
Data Analysis:
RDP
QIIME
Alpha Diversity
Machine Learning
Manual Data Analysis:
Machine Learning - Manual
Alpha Diversity
Data Selector
Refresh
Data Filter:
Value
Groups
GMT_Tutorial
Databases
gmtDB
All Annotations in Database
Tracks
SampleSets
Samples
Files
MicrobiomeWorkBench
GMT_Tutorial_Study
QIIME
Qiime-Job-2011-08-05-16:22:08
RDP
MicrobiomeData
tutorial_sequence_file.sff.gz
tutorial_meta_data.tsv
Input Data
Qiime-Job-2011-08-05-16:22:08
Output Targets
gmtDB
gmtProject

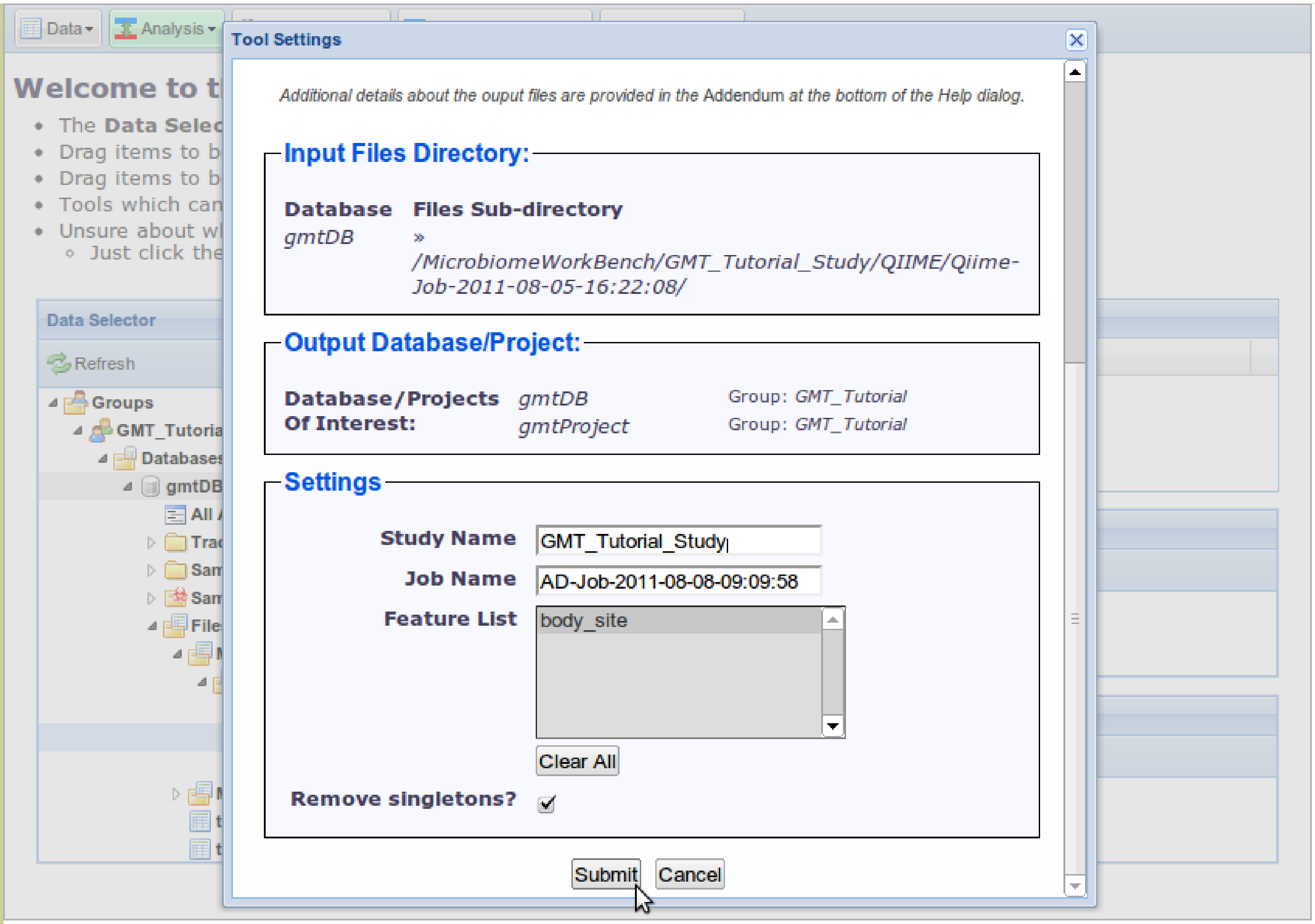


```
Hello Tutorial Imt

Your Alpha Diversity job is complete successfully.

Job Summary:
  JobID                    : wbJob-alphadiversity-1312812652_756847
  Study Name               : GMT_Tutorial_Study
  Job Name                 : AD-Job-2011-08-08-09_09_58

Result File Location in the Genboree Workbench:
  Group : GMT_Tutorial
  DataBase : gmtDB
  Path to File:
     Files
     * MicrobiomeData
        * GMT_Tutorial_Study
           *AlphaDiversity
              *AD-Job-2011-08-08-09:09:58

Plots URL (click or paste in browser to access file):
   Prj: gmtProject
   URL:
http://genboree.org/java-bin/project.jsp?projectName=gmtProject

The Genboree Team
```

Alpha Diversity Results

- Click the *Refresh* button in the *Data Selector* window
- Expand `Groups` -> `GMT_Tutorial` -> `Databases` -> `gmtDB` -> `Files` -> `MicrobiomeWorkBench` -> `Tutorial_Study` -> `AlphaDiversity` -> `AD-Job-2011-07-18-10:48:32`
  - rankAbundancePlots.result.tar.gz
    - Rank abundance plots for all meta data features selected
  - renyiProfilePlots.result.tar.gz
    - Renyi profile plots for all meta data features selected
  - sample.mapping.txt
  - settings.json

- raw.result.tar.gz
  - Full output data set including R scripts used to generate plots
- richnessPlots.result.tar.gz
  - Richness plots for all meta data features selected
- jobFile.json

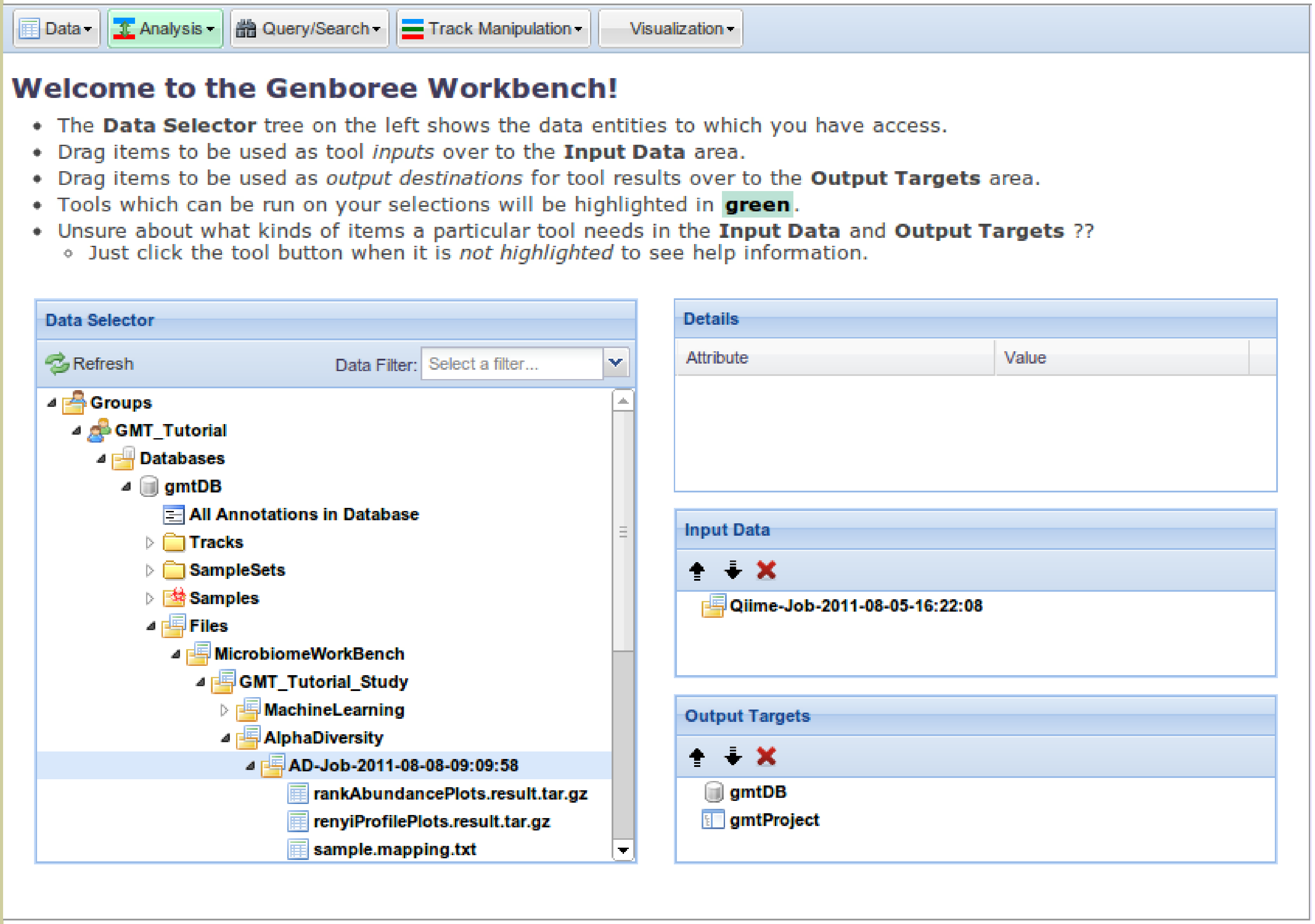

(This is a recently added feature. Report issues to **Genboree Admin**.)

Edit Mode

gmtProject

[[Put description for the project 'gmtProject' here]]

Project News:

2011/8/8: Tutorial Imt ran a Alpha Diversity job (AD-Job-2011-08-08-09:09:58) and the results are available at the link below.

- **Study Name**: GMT_Tutorial_Study
- **Job Name**: AD-Job-2011-08-08-09:09:58
- **Link to result plots**

2011/8/8: Tutorial Imt ran a Machine Learning job (ML-Job-2011-08-08-09:13:04) and the results are available at the link below.

- **Study Name**: GMT_Tutorial_Study
- **Job Name**: ML-Job-2011-08-08-09:13:04
- **Link to result plots**

2011/8/5: Tutorial Imt ran a QIIME job (Qiime-Job-2011-08-05-16:22:08) and the results are available at the links below.

- **Study Name**: GMT_Tutorial_Study
- **Job Name**: Qiime-Job-2011-08-05-16:22:08
- **Link to cdhit results**
- **Link to cdhit-normalized results**
- **Link to phylogenetic tree results**

2011/8/5: Tutorial Imt ran a RDP job (RDP-Job-2011-08-05-16:19:52) and the results are available at the link below.

- **Study Name**: GMT_Tutorial_Study
- **Job Name**: RDP-Job-2011-08-05-16:19:52
- **Link to result plots**

Table of Content: Alpha Diversity Results

Study Name: GMT_Tutorial_Study
Job Name: AD-Job-2011-08-08-09:09:58
User: Tutorial Imt
Date: 2011/08/08 09:11 CDT

Alpha Diversity Plots

By Plot Type	By Feature Type
• Plot: Richness<br>◦ Subplot: Coleman<br>▪ Feature: body_site<br>◦ Subplot: Collector<br>▪ Feature: body_site<br>◦ Subplot: Exact<br>▪ Feature: body_site<br>◦ Subplot: Random<br>▪ Feature: body_site<br>◦ Subplot: Rarefaction<br>▪ Feature: body_site<br>• Plot: Renyiprofile<br>◦ Subplot: Renyi<br>▪ Feature: body_site<br>◦ Subplot: Evenness-renyi<br>▪ Feature: body_site<br>• Plot: Rankabundance<br>◦ Subplot: Abundance<br>▪ Feature: body_site	• Feature: body_site<br>◦ Plot: Richness<br>▪ Subplot: coleman<br>▪ Subplot: collector<br>▪ Subplot: exact<br>▪ Subplot: random<br>▪ Subplot: rarefaction<br>◦ Plot: Renyiprofile<br>▪ Subplot: renyi<br>▪ Subplot: evenness-renyi<br>◦ Plot: Rankabundance<br>▪ Subplot: abundance<br>▪ Subplot: proportion<br>▪ Subplot: logabun<br>▪ Subplot: accumfreq

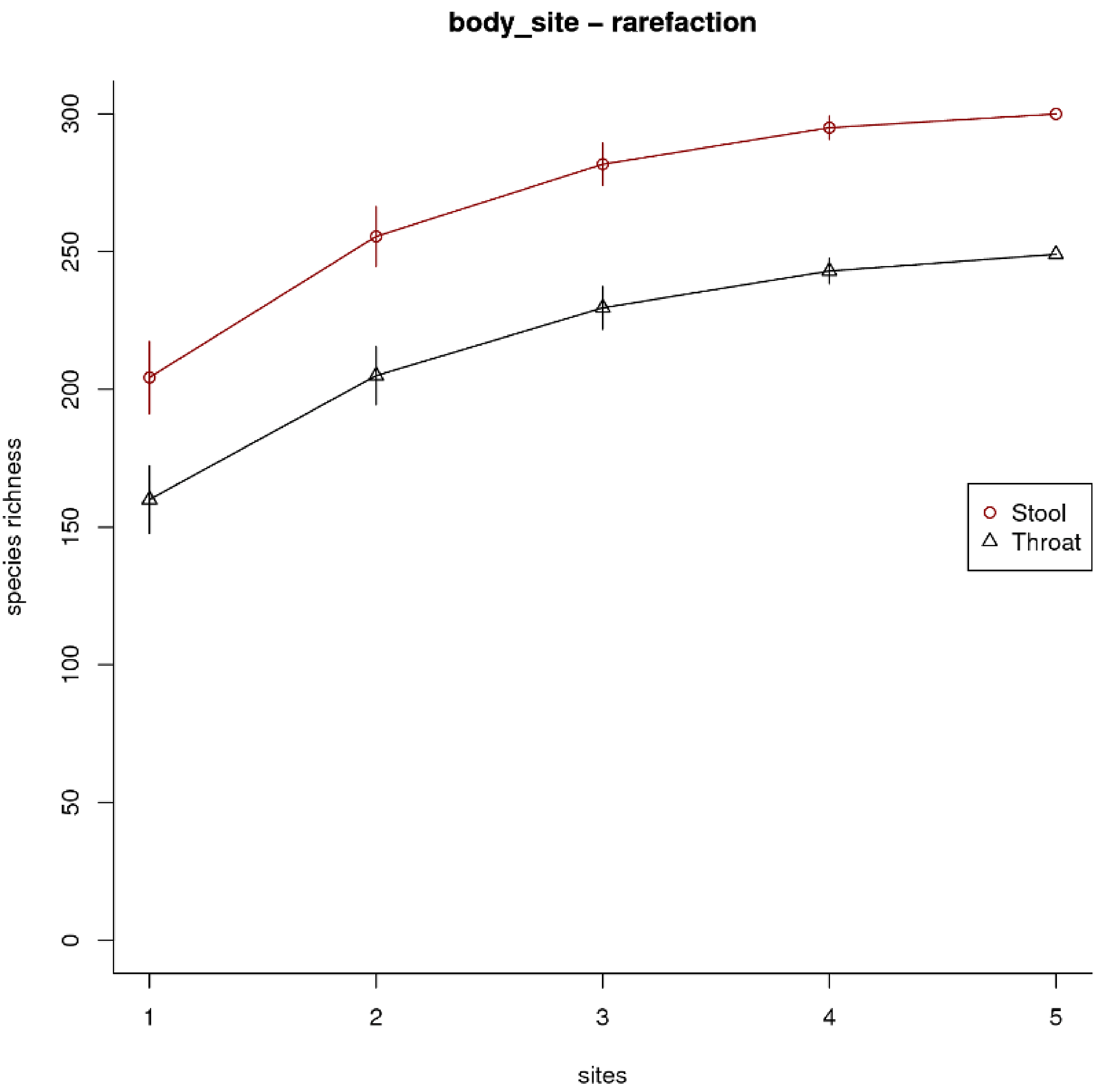

Machine Learning

- Drag `Qiime-Job-2011-08-05-16:22:08` into the *Input Data* window
  - Accessible via `Groups` -> `Databases` -> `gmtDB` -> `Files` -> `MicrobiomeWorkBench` -> `Tutorial_Study` -> `QIIME` -> `Qiime-Job-2011-08-05-16:22:08`
- Drag over the `gmtDB` into the *Output Targets* window
- Click the *Analysis* tab, followed by the *Microbiome Workbench* tab, followed by the *Machine Learning* tab
- Optionally fill in a 'Study Name', here we'll use 'Tutorial_Study'
- Select one or many feature lists, which was accessible via the user provided sample meta data
- Click 'Submit'
- Wait for confirmation email

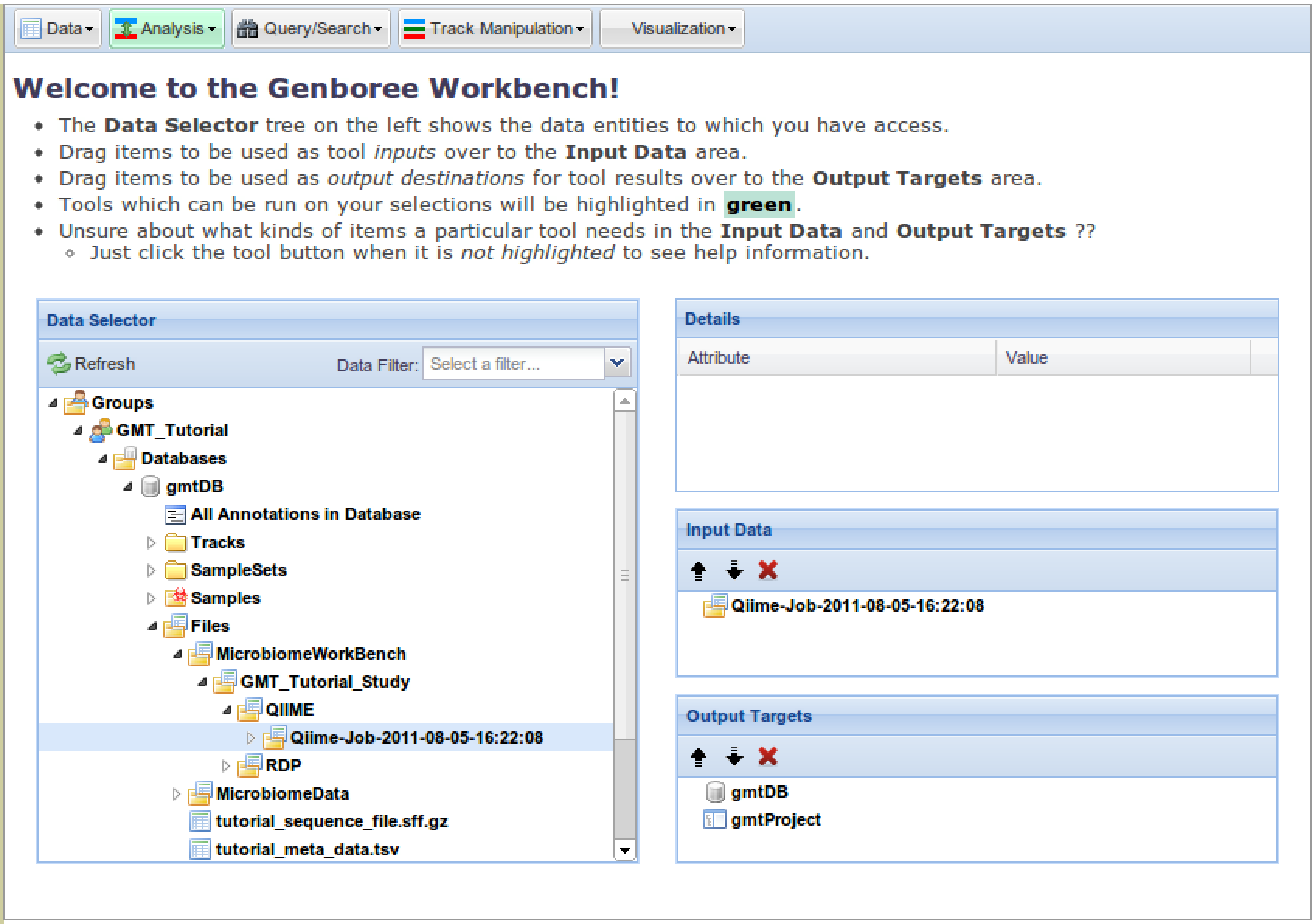
Data
Analysis
Query/Search
Track Manipulation
Visualization
Welcome to the Genboree Workbench!
The Data Selector tree on the left shows the data entities to which you have access.
Drag items to be used as tool inputs over to the Input Data area.
Drag items to be used as output destinations for tool results over to the Output Targets area.
Tools which can be run on your selections will be highlighted in green.
Unsure about what kinds of items a particular tool needs in the Input Data and Output Targets ??
Just click the tool button when it is not highlighted to see help information.
Data Selector
Refresh
Data Filter:
Select a filter...
Groups
GMT_Tutorial
Databases
gmtDB
All Annotations in Database
Tracks
SampleSets
Samples
Files
MicrobiomeWorkBench
GMT_Tutorial_Study
QIIME
Qiime-Job-2011-08-05-16:22:08
RDP
MicrobiomeData
tutorial_sequence_file.sff.gz
tutorial_meta_data.tsv
Details
Attribute
Value
Input Data
Qiime-Job-2011-08-05-16:22:08
Output Targets
gmtDB
gmtProject

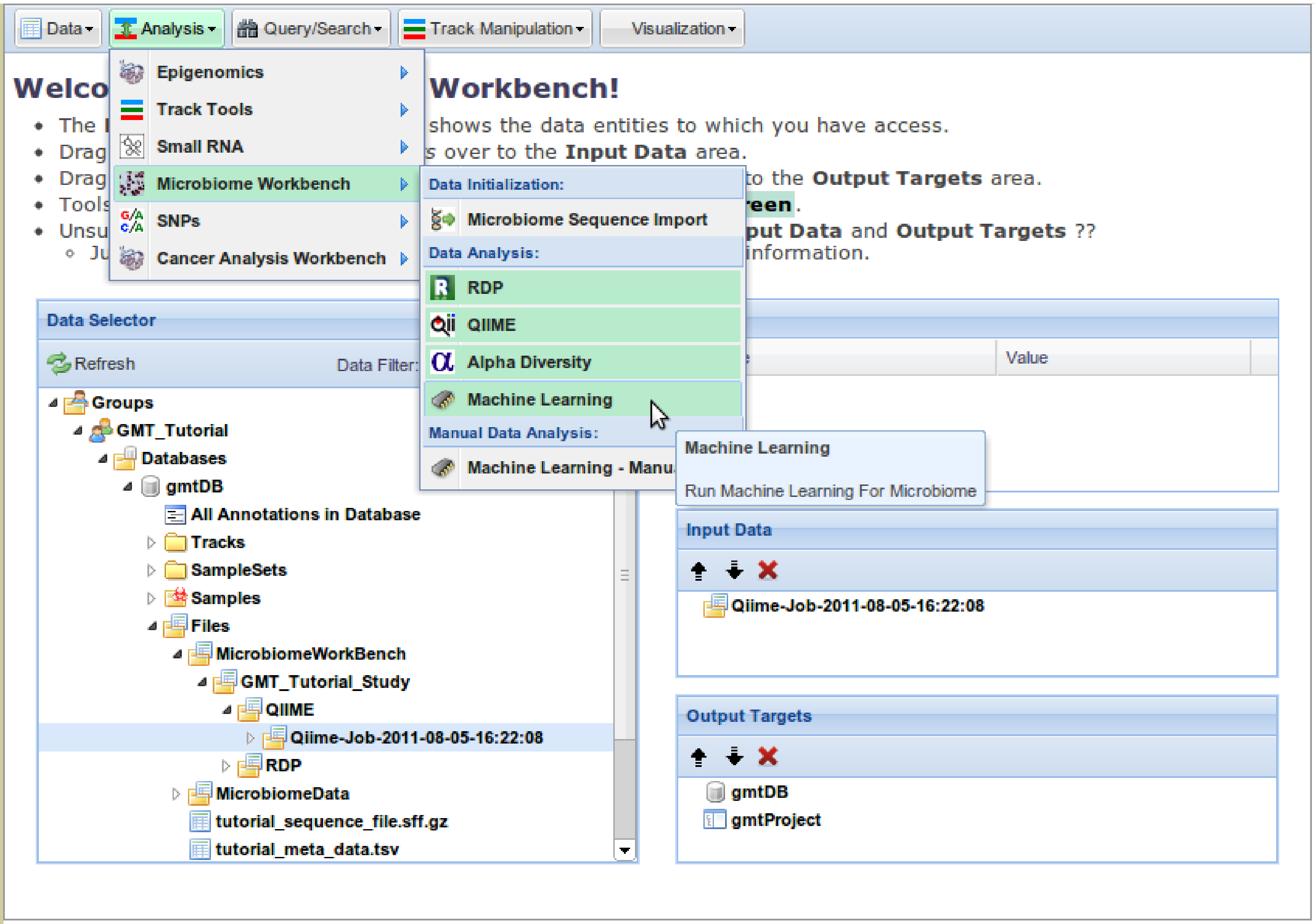

Data
Analysis
Query/Search
Track Manipulation
Visualization
Epigenomics
Track Tools
Small RNA
Microbiome Workbench
SNPs
Cancer Analysis Workbench
Data Initialization:
Microbiome Sequence Import
Data Analysis:
RDP
QIIME
Alpha Diversity
Machine Learning
Manual Data Analysis:
Machine Learning - Manu
Machine Learning
Run Machine Learning For Microbiome
Workbench!
shows the data entities to which you have access.
s over to the Input Data area.
to the Output Targets area.
reen.
put Data and Output Targets ??
information.
Data Selector
Refresh
Data Filter:
Value
Groups
GMT_Tutorial
Databases
gmtDB
All Annotations in Database
Tracks
SampleSets
Samples
Files
MicrobiomeWorkBench
GMT_Tutorial_Study
QIIME
Qiime-Job-2011-08-05-16:22:08
RDP
MicrobiomeData
tutorial_sequence_file.sff.gz
tutorial_meta_data.tsv
Input Data
Qiime-Job-2011-08-05-16:22:08
Output Targets
gmtDB
gmtProject

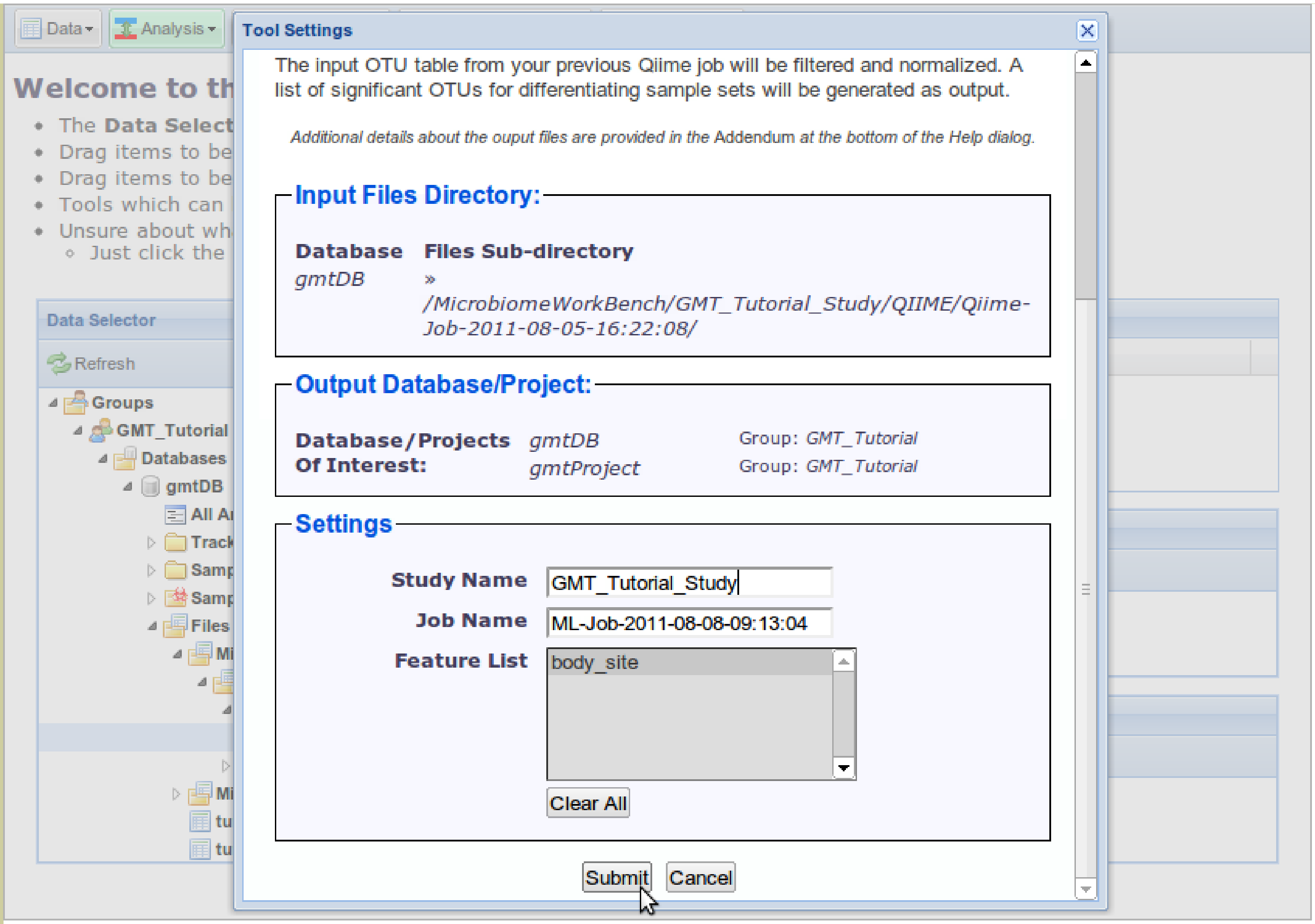


```
Hello Tutorial Imt

Your Machine Learning job is complete successfully.

Job Summary:
  JobID                    : wbJob-machinelearning-1312812804_274522
  Study Name               : GMT_Tutorial_Study
  Job Name                 : ML-Job-2011-08-08-09_13_04

Result File Location in the Genboree Workbench:
  Group : GMT_Tutorial
  DataBase : gmtDB
  Path to File:
     Files
     * MicrobiomeData
        * GMT_Tutorial_Study
           *MachineLearning
              *ML-Job-2011-08-08-09:13:04

Plots URL (click or paste in browser to access file):
   Prj: gmtProject
   URL:
http://genboree.org/java-bin/project.jsp?projectName=gmtProject

The Genboree Team
```

Machine Learning Results

- Click the *Refresh* button in the *Data Selector* window
- Expand `Groups` -> `GMT_Tutorial` -> `Databases` -> `gmtDB` -> `Files` -> `MicrobiomeWorkBench` -> `Tutorial_Study` -> `MachineLearning` -> `ML-Job-2011-08-08-09_13_04`
  - jobFile.json
  - sample.mapping.txt
  - settings.json
  - otu_abundance_cutoff_(5/25/100/500).result.tar.gz
    - (5/25/100/500)_bag.txt
      - randomForest classification result

- (5/25/100/500)_sortedImportance.txt
  - randomForest imMportance sorted by 'MeanDecreaseGini'
- raw.result.tar.gz
  - Full results from machine learning pipeline
  - Summary reports exist within `raw.result` -> `RF_Boruta` -> `body_site` -> `RandomForest` -> (5/25/100/500)_sortedImportanceforcombine.gini_trends_3sorted
  - Or you can take advantage of the summary xls sheet which summarizes the OOB error estimate `RF_Summary.xls`

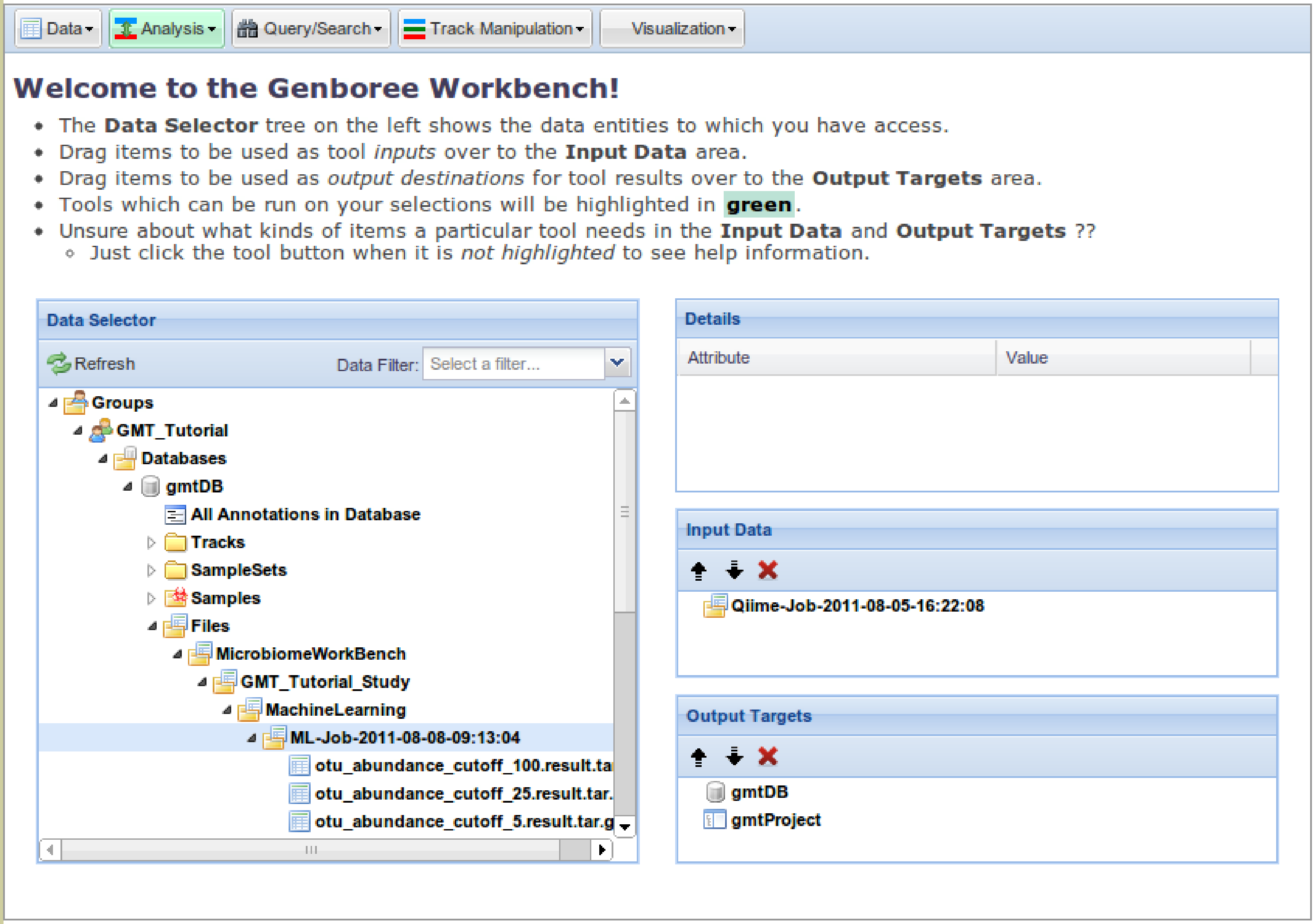

Table of Content: RDP Results

Study Name: GMT_Tutorial_Study
Job Name: ML-Job-2011-08-08-09:13:04
User: Tutorial Imt
Date: 2011/08/08 09:15 CDT

body_site

Body_site-25
Body_site-500
Body_site-5
Body_site-100

	5	25	100	500
body_site	0.0	0.0	0.0	0.0
